# Excess weight by degree and duration and cancer risk (ABACus2 consortium): a cohort study and individual participant data meta-analysis

**DOI:** 10.1016/j.eclinm.2024.102921

**Published:** 2024-11-19

**Authors:** Nadin K. Hawwash, Matthew Sperrin, Glen P. Martin, Rashmi Sinha, Charles E. Matthews, Fulvio Ricceri, Anne Tjønneland, Alicia K. Heath, Marian L. Neuhouser, Corinne E. Joshu, Elizabeth A. Platz, Heinz Freisling, Marc J. Gunter, Andrew G. Renehan

**Affiliations:** a Division of Cancer Sciences, School of Medical Sciences, Faculty of Biology, Medicine and Health, University of Manchester, Manchester, United Kingdom; b Cancer Research UK Manchester Cancer Research Centre, Manchester, United Kingdom; c Centre for Health Informatics, Division of Informatics, Imaging and Data Sciences, School of Health Sciences, Faculty of Biology, Medicine and Health, University of Manchester, Manchester, United Kingdom; d Metabolic Epidemiology Branch, Division of Cancer Epidemiology and Genetics, National Cancer Institute, Shady Grove, USA; e Centre for Biostatistics, Epidemiology, and Public Health (C-BEPH), Department of Clinical and Biological Sciences, University of Turin, Regione Gonzole 10, Orbassano (TO), Italy; f Danish Cancer Institute, Strandboulevarden 49, 2100 Copenhagen O, Denmark; g Cancer Epidemiology and Prevention Research Unit, School of Public Health, Imperial College London, London, W2 1PG, United Kingdom; h Cancer Prevention Program, Division of Public Health Sciences, Fred Hutchinson Cancer Center, Seattle, WA, USA; i Department of Epidemiology, Johns Hopkins Bloomberg School of Public Health, Baltimore, MD, USA; j Nutrition and Metabolism Branch, International Agency for Research on Cancer (IARC-WHO), Lyon, France; k National Institute for Health Research (NIHR) Manchester Biomedical Research Centre, Manchester, United Kingdom

**Keywords:** Obesity, Life course, Cancer

## Abstract

**Background:**

Elevated body mass index (BMI) ≥25 kg/m^2^ is a major preventable cause of cancer. A single BMI measure does not capture the degree and duration of exposure to excess BMI. We investigate associations between adulthood overweight-years, incorporating exposure time to BMI ≥25 kg/m^2,^ and cancer incidence, and compare this with single BMI.

**Methods:**

In this cohort study and individual participant data meta-analysis, we obtained data from the ABACus 2 Consortium, consisting of four US cohorts: Atherosclerosis Risk in Communities (ARIC) study (1987–2015), Women's Health Initiative (WHI; 1991 to 2005 [main study], to 2010 [Extension 1], and to 2020 [Extension 2]), Prostate, Lung, Colorectal, Ovarian Cancer Screening (PLCO) Trial (1993–2009), NIH-AARP Diet and Health Study (1996–2011), and one European cohort, the European Prospective Investigation into Cancer and Nutrition (EPIC; participants enrolled in 1990 and administrative censoring was centre-specific). Participants with at least 3 BMI measurements and complete cancer follow-up data were included. We calculated overweight-years: degree of overweight (BMI ≥25 kg/m^2^) multiplied by the duration of overweight (years). Using random effects two-stage individual participant data meta-analyses, associations between cancer and overweight-years, single BMI, cumulative overweight degree and duration, measured at the same time and captured over a median of 41 years in men and 39 years in women, were evaluated with Cox proportional hazards models. Models were age-adjusted or multivariable (MV) adjusted for baseline age, ethnicity, alcohol, smoking and hormone replacement therapy (HRT). Harrell's C-statistic of metrics were compared. This study is registered at PROSPERO, CRD42021238270.

**Findings:**

720,210 participants, including 312,132 men and 408,078 women, were followed up for cancer incidence over a median 9.85 years (interquartile range (IQR) 8.03, 11.67) in men and 10.80 years (IQR 6.05, 15.55) in women. 12,959 men (4.15%) and 36,509 women (8.95%) were diagnosed with obesity-related cancer. Hazard ratios for obesity-related cancers in men, per 1 standard deviation (SD) overweight-years were 1.15 (95% CI: 1.14, 1.16, I^2^: 0) age-adjusted and 1.15 (95% CI: 1.13, 1.17, I^2^: 0%) MV-adjusted and per 1SD increment in single BMI were 1.17 (95% CI: 1.16, 1.18, I^2^: 0) age-adjusted and 1.16 (95% CI: 1.15, 1.18, I^2^: 0%) MV-adjusted. The HR for overweight-years in women per 1 SD increment was 1.08 (95% CI: 1.04, 1.13, I^2^: 82%) age-adjusted and 1.08 (95% CI: 1.04, 1.13, I^2^: 83%) MV-adjusted and per 1SD increment in single BMI was 1.10 (95% CI: 1.07, 1.14, I^2^: 72%) age-adjusted and 1.11 (95% CI: 1.07, 1.15, I^2^: 79%) MV-adjusted. C-statistics for overweight-years and single BMI for obesity-related cancers were 0.612 (95% CI: 0.578, 0.646) and 0.611 (95% CI: 0.578, 0.644) respectively for men and 0.566 (95% CI: 0.534, 0.598) and 0.573 (95% CI: 0.546, 0.600) for women.

**Interpretation:**

Adulthood degree and duration of excess BMI were associated with cancer risk. Both factors should be considered in cancer prevention strategies and policies. This study only focused on adulthood exposure to excess BMI, so the minimal differences in the predictive performance between adiposity metrics may be due to underestimation of cumulative excess BMI exposure.

**Funding:**

10.13039/501100000289Cancer Research UK, the 10.13039/100014653Manchester NIHR Biomedical Research Centre, the 10.13039/100000054National Cancer Institute, the 10.13039/100000050National Heart, Lung, and Blood Institute, National Institutes of Health, Department of Health and Human Services, 10.13039/100000016U.S. Department of Health and Human Services, the Intramural Research Program of the National Cancer Institute, the 10.13039/100008700International Agency for Research on Cancer, Imperial College London, European Commission (DG-SANCO), the 10.13039/100008363Danish Cancer Society, 10.13039/501100004099Ligue Contre le Cancer, 10.13039/501100008017Institut Gustave-Roussy, 10.13039/501100008018Mutuelle Générale de l'Education Nationale, 10.13039/501100001677Institut National de la Santé et de la Recherche Médicale, 10.13039/501100005972Deutsche Krebshilfe, 10.13039/100008658Deutsches Krebsforschungszentrum, 10.13039/501100002347German Federal Ministry of Education and Research, the 10.13039/501100018706Hellenic Health Foundation, Associazione Italiana per la Ricerca sul Cancro-AIRC-Italy and National Research Council, Dutch Ministry of Public Health, Welfare, and Sports, Netherlands Cancer Registry, LK Research Funds, Dutch Prevention Funds, Dutch Zorg Onderzoek Nederland, 10.13039/501100000321World Cancer Research Fund, Statistics Netherlands, Health Research Fund, 10.13039/501100004587Instituto de Salud Carlos III, regional Spanish governments of Andalucía, Asturias, Basque Country, Murcia, and Navarra, the Catalan Institute of Oncology, 10.13039/501100002794Swedish Cancer Society, Swedish Scientific Council, and Region Skåne and Region Västerbotten, and the 10.13039/501100000265Medical Research Council.

**Research in context**

**Evidence before this study**

We searched PubMed to identify the relevant studies published until September 2023 using the terms “life course”, “overweight-years”, “obese-years”, and “obesity-related cancer”. References from identified papers were searched. At least 13 cancers are linked to excess BMI; however, evidence on the cumulative degree and duration of excess BMI on cancer risk combined using overweight-years and analysed separately is scarce. Current studies often have small sample sizes, focus on one cancer type or country or rely on biased electronic health record data, overrepresenting those with repeated BMI and comorbidities. Our analysis across 10 countries and cancer types will highlight the importance of cancer policy and prevention strategies focusing on minimising cumulative exposure to excess adiposity.

**Added value of this study**

We have shown that both the degree and duration of excess BMI exposure are associated with cancer risk. Positive associations were found between overweight-years and colorectal, pancreatic, kidney and bladder cancer in men and colorectal, kidney and endometrial cancers in women. The duration of overweight outperformed the degree of overweight in predicting lung cancer risk in men and kidney cancer in women. Overweight-years did not outperform a single BMI measurement in predicting cancer risk.

**Implications of all the available evidence**

The degree and duration of exposure to excess BMI in adulthood should be considered in cancer prevention strategies and policies. Single BMI can continue to be used to predict obesity-related cancer risk given its simplicity and predictive performance. However, analysis across the whole life course and other populations is needed to identify whether findings are generalisable to populations beyond those explored.

## Introduction

A public health concern is the rapidly rising prevalence of obesity, with worldwide mean body mass index (BMI) increases of 2.5 kg/m^2^ in men and 2.1 kg/m^2^ in women between 1974 and 2014.^1^ BMI is determined by dividing an individual's weight in kilograms by their height in meters squared. Thirteen cancer sites are related to overweight (BMI ≥25 kg/m^2^) and obesity (BMI ≥30 kg/m^2^) as stated in the 2016 International Agency for Research on Cancer (IARC) report, which highlighted sufficient evidence indicating causal relationships between adiposity and cancer, primarily measured using BMI assessed at a single timepoint.^2^ Effective obesity-related cancer prevention is required. Here, we postulate that a single BMI measurement does not sufficiently capture relevant adiposity exposure. The accumulated time spent at a level of excess adiposity may help inform the underlying biological mechanisms involved. Thus, defining the degree and duration of excess BMI exposure may be most etiologically relevant to cancer aetiology.

The overweight-years metric quantifies cumulative excess BMI exposure by including the degree (number of BMI units ≥25 kg/m^2^) and duration of overweight (in years), comparable to tobacco smoking pack-years (a cumulative measure of smoking exposure).^3^, ^4^, ^5^, ^6^, ^7^, ^8^ Similar metrics include obese-years incorporating exposure time to BMI ≥30 kg/m^2^. A recent study analysed the adiposity-related cancer risk over adulthood using electronic health records (EHRs) of >2.6 million adults in Catalonia, Spain and found a longer duration and a greater degree of excess BMI were positively associated with 18 cancers.^9^ However, there is a potential selection bias with the correlation previously found between those with repeated BMI measurements in EHRs and co-morbidities.^10^ Additionally, cancer-reported data was used which has been previously shown to vary from cancer registry data, especially for rarer cancer sites.^11^^,^^12^ Our study will analyse the cumulative degree and duration of exposure to excess adiposity over adulthood using several populations and ancestries through recall and prospective cohorts where repeated BMI missingness numbers were random, and cancer ascertainment was through cancer registries. In cancer epidemiology, research is limited on whether overweight-years has increased predictive performance, i.e., the ability to distinguish participants according to their cancer risk, instead of—or alongside—single BMI measurement.^5^^,^^6^

The study objectives were to 1) analyse associations between overweight-years, single BMI and cancer incidence, including the metric component parts (cumulative degree and duration of excess BMI), and 2) compare predictive performances of overweight-years with single BMI, both measured at the same time.

## Methods

### Study population and data

We assembled the ABACus 2 Consortium of over 1.4 million participants, including the 1990 European Prospective Investigation into Cancer and Nutrition (EPIC) study^13^ and four US studies, the 1987 Atherosclerosis Risk in Communities study (ARIC),^14^ 1991 Women's Health Initiative (WHI),^15^ 1993 Prostate, Lung, Colorectal, Ovarian Cancer Screening Trial (PLCO)^16^ and 1996 NIH-AARP Diet and Health Study (NIH-AARP).^17^ Cohorts were selected based on the availability of repeated BMI measurements and cancer follow-up (Table S1). The analyses were performed stratified by sex.

Given the use of secondary cohort data, no ethical approval was required for the current analysis. Anonymous data was handled as per data management plan and data transfer agreements.^18^ We included in the analysis only those ARIC participants who consented to the use of their data more broadly than just for cardiovascular research. The Institutional Review Boards at each study site approved the ARIC study protocol. The NIH-AARP Diet and Health Study was reviewed and approved by the Special Studies Institutional Review Board of the US National Cancer Institute, and all participants gave written informed consent by virtue of completing and returning the questionnaire.

### Eligibility criteria

We excluded participants who at baseline: 1) were over 80 years of age given weight decline beyond 80 years due to skeletal muscle loss, which can lead to lower BMI for the same adiposity; 2) had prior cancers; 3) had <3 BMI measurements and 4) had missing cancer follow-up data. Extreme BMI measurements (≤15 kg/m^2^ and ≥60 kg/m^2^) were excluded.

### Exposure

Within the 5 cohorts, as our main analysis, we derived a design of 3 BMI measurements as the exposure. Follow-up started from the third measure, is referred to as the index date (Figure S1). The primary exposure was BMI, quantified at baseline: (i) as a single BMI measurement, and (ii) overweight-years. Overweight-years were quantified using predicted BMI measurements (see 2.6 Statistical analysis) from ages 18 in WHI and NIH-AARP, age 20 in PLCO up to and including the cohort entry BMI measurement (In Figure S1, these are referred to as ‘recall’ studies). Mean periods of exposure were 46 years in WHI, 42 years for men and 39 years for women in NIH-AARP, 43 years for men and women in PLCO. The ARIC study contained one recall BMI only, at age 25, such that Visit 2 was taken at the index date.^19^ In EPIC, cancer follow-up started 5 years after study entry so the start of follow-up was landmarked to this time (to avoid immortal person-time).^20^ In Figure S1, these are referred to as ‘prospective’ studies). Mean periods of exposure were 40 years for men, 37 for women in ARIC and 35 years for men and 32 years for women in EPIC. In the ‘prospective’ studies, the exclusion criteria were applied by the index date.

We calculated overweight-years using yearly predicted BMI, by multiplying prior overweight degree (BMI units ≥25 kg/m^2^; <25 kg/m^2^ = 0) by the duration of that overweight degree–the time in years between the prior and current observation (Table S2). BMI was subtracted by 24.9 and not 25 to include BMI readings at 25 kg/m^2^. Total cumulative overweight-years was the sum of prior overweight-years. Overweight-years assumes cumulative degree and duration of overweight contribute equally to cancer risk; therefore, degree and duration were analysed separately to identify their independent contributions to cancer risk. Total cumulative overweight degree and duration were the sum of prior overweight degree and duration, respectively.

### Outcomes

The primary outcome was total cancer incidence, subdivided into obesity-related and non-obesity-related cancers. End of follow-up was defined as the first primary cancer diagnosis, end of cancer follow-up, or death, whichever occurred first (Figure S1). Cause-specific compelling risks analysis was completed in this study, with participants being censored at the time of death if it occurred prior to the cancer event. Obesity-related cancers were: colorectal, gastric, oesophageal, thyroid, kidney, liver, pancreatic, multiple myeloma, gallbladder, meningioma, postmenopausal breast, ovarian and endometrial cancers. Non-obesity-related cancers were total cancers minus obesity-related cancers, except for EPIC, where non-melanoma skin cancers were excluded. In further analyses, associations with non-obesity-related cancers less lung and prostate cancers were performed. Cancer sites with ≥10 events per candidate predictor parameter were studied separately.^21^ Colorectal, pancreatic, kidney, bladder, lung and prostate cancers in men and colorectal, postmenopausal breast, endometrial, ovarian, kidney, lung and pancreatic cancer in women were thus analysed. A breast cancer diagnosis at or above age 55 was defined as postmenopausal breast cancer.

### Covariates

Covariates harmonised and included were race categorised into ‘White’, ‘Black’ and ‘Other’; smoking categorised into “ever smokers” and “never smokers”; hormone replacement therapy (HRT) categorised as “ever HRT users” and “never HRT users” and alcohol (units/week).

### Statistical analysis

Descriptive characteristics were calculated using the mean and standard deviation (SD) of continuous variables, and prevalences for categorical variables. We conducted a random effect two-stage IPD meta-analysis. First, we analysed cohorts separately. We imputed missing covariate data listed in Section 2.5 using multiple imputation with predictive mean matching which resulted in 10 imputed datasets per cohort.^22^ Variables in the predictor matrix were: “race”, “smoking”, “education”, “HRT”, “alcohol”, “ever diagnosed with heart disease”, “ever diagnosed with diabetes”, “age of cancer diagnosis”, “cancer incidence”. Imputed datasets were checked for convergence and subsequent analyses were performed on each imputed dataset before pooling results using Rubin's rules.^23^ BMI was then predicted yearly over adulthood.

Second, for each cohort, linear prediction models were used to predict BMI each year over adulthood. In order to choose the appropriate prediction model, we compared linear prediction models with an interaction between sex and age. We compared models with i) a random intercept, ii) with a random intercept and a random slope, iii) model (ii) with a spline on age and, iv) model (iii) with varying numbers and positions of knots set for the restricted cubic spline. A linear prediction model with a random intercept, random slope and spline on age was used given it had the lowest Akaike information criterion, hence the greatest model fit. Following BMI prediction per year across adulthood, overweight-years was subsequently calculated. The covariates listed in Section 2.5 were included as fixed effects and estimated within each cohort individually.

Associations between overweight BMI metrics and cancer incidence were estimated and reported per one standard deviation increase in exposure by fitting Cox proportional hazards models, from baseline (median age 60.9 years (IQR 2.9) in men and 63.0 years (IQR 3.8) in women) to cancer incidence. In these models, overweight-years was a continuous, time-fixed variable adjusted for age at baseline separately in an age-adjusted model, and adjusted for age at baseline, race, alcohol, smoking and HRT (in women) in a multivariable-adjusted model given participants entered the study at different ages and race, alcohol, smoking and HRT are potential confounders of the association between excess adiposity and cancer. We calculated HRs of cancer per 1 SD increment in cumulative overweight degree and duration separately. Cox proportional hazards assumptions were tested with Schoenfeld residuals. The categorical variable, smoking, which violated the Cox proportional hazards assumption was stratified and for the continuous variable, baseline age, which violated the assumption, time-varying coefficients were included. HRs per 100 overweight-years (kg-years/m^2^), per 5 kg/m^2^ single BMI, per 10 kg/m^2^ cumulative overweight degree and per 10-year cumulative overweight duration were calculated to allow comparison with prior literature that used such measures. Multivariable-adjusted HRs are reported.

Following cohort analysis, we derived summary effects for each meta-analysis and I^2^ measures to quantify the total variability due to between-study heterogeneity.^24^ First, we expressed associations per unit SD of each metric to standardise the values and analyse associations relative to the average and range. Second, we calculated C-statistics of overweight-years, single BMI, overweight-years adjusted for single BMI, cumulative overweight degree and duration, and adjusted for in-sample optimism with bootstrapping 100 times.^25^ Harrell's C-statistic measured the discriminatory predictive performance of metrics and C-statistic differences showed variations in model discrimination.

### Sensitivity analysis

Analyses were repeated using obese-years (degree of obesity (BMI ≥30 kg/m^2^) multiplied by the duration (years) of obesity) (Table S3). The main analyses were repeated i) using measured and not predicted BMI to calculate overweight-years and ii) using BMI predicted from participants in each cohort with ≥1 BMI measurement (Figure S2).

High-Performance Computing clusters and R 4.1.2 (RRID:SCR_001905) and the following packages lme 4,^26^ survival,^27^ rms,^28^ ggplot 2,^29^ tidyverse,^30^ purrr,^31^ gtsummary,^32^ splines,^33^ and Hmisc^34^ were used. The study was reported according to PRISMA-IPD guidelines. This study is registered at PROSPERO (CRD42021238270).

### Role of the funding source

The funder of the study had no role in study design, data collection, data analysis, data interpretation, or writing of the report. All authors had full access to their cohort data in the study and had final responsibility for the decision to submit for publication.

## Results

### Baseline characteristics

**Fig. 1:**
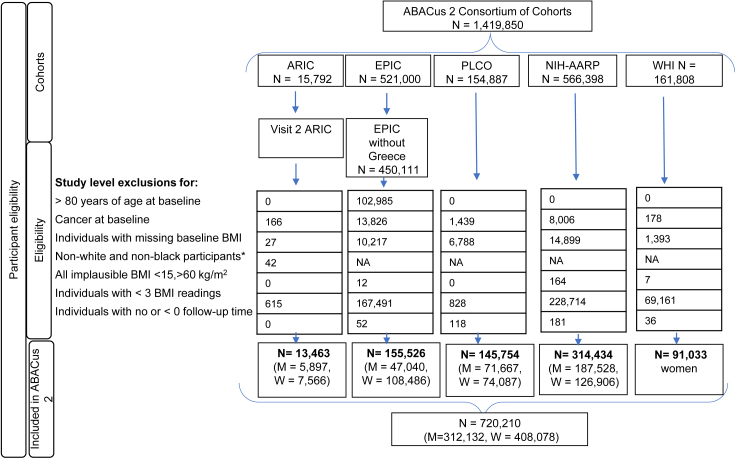
**ABACus 2 Consortium Participant Flow Diagram.** BMI-related exclusion criteria were observational level exclusions but resulted in individual exclusions if none of the BMI readings were within the clinically plausible range. Abbreviations: ARIC, Atherosclerosis Risk in Communities (Study); EPIC, European Prospective Investigation into Cancer and Nutrition (Study); PLCO, Prostate, Lung, Colorectal and Ovarian Cancer (Screening Trial); NIH-AARP, NIH-AARP Diet and Health Study; WHI, Women's Health Initiative; BMI, body mass index; N, number of participants; M, men; W, women; HRT, hormone replacement therapy.

720,210 participants (57% women) out of 1,491,850 in the ABACus 2 consortium were included (Fig. 1, Table 1). 85,341 men and 63,732 women were diagnosed with cancer over median follow-up periods across studies of 9.85 years (interquartile range (IQR) 8.03, 11.67) and 10.80 years (IQR 6.05, 15.55), respectively (Table S4). A detailed breakdown of incident cancer cases over the follow-up period in the included cohort is provided in Table S4. At baseline, men had a median age of 60.90 years (IQR 59.45, 62.35) and a mean BMI of 27.18 kg/m^2^ and women had a median age of 63.0 years (IQR 61.10, 64.90) and a mean BMI of 26.92 kg/m^2^. In studies that recorded race, proportion of Black participants ranged across cohorts between 2% and 20% for men and 5%–28% for women. The proportion of ever smokers ranged between 64% and 74% for men and 44%–55% for women. Between 28% and 67% of women were ever HRT users (Table 1). The magnitude of a one SD increase in overweight-years ranged across cohorts in the ABACus 2 consortium from 65 to 81 kg-years/m^2^ for men and 61 and 132 kg-years/m^2^ for women; for cumulative overweight degree, the SD ranged between 67 and 84 kg/m^2^ for men and 63 and 104 kg/m^2^ for women and the SD ranged between 12 and 16 years for men and 13 and 18 years for women across the cohorts for cumulative overweight duration. The mean and standard deviation of each exposure metric specific to each cohort in the ABACus 2 consortium is shown in Table S4. All-cause and cancer mortality of cohorts overall and subgroups included in this study is shown in Table S5. Few variations in characteristics were seen in participants of the ABACus 2 consortium excluded from the main analysis of this study shown in Table S6 aside from the relatively higher percentage of never-HRT users in women that were excluded and the relatively greater proportion of Black men excluded from the ARIC study of 38% compared with the 20% included in our study. Few variations in participants' characteristics were also found between those with ≥1 BMI and those with ≥3 BMI measurements (Table S7).

**Table 1:** Characteristics^a^ of the analytic cohorts.

Characteristic	Men				Women				
	ARIC N = 5897	PLCO N = 71,667	NIH-AARP N = 187,528	EPIC N = 47,040	ARIC N = 7566	PLCO N = 74,087	NIH-AARP N = 126,906	EPIC N = 108,486	WHI N = 91,033
Age^a^, years, mean (SD)	57.0 (6.0)	63.0 (5.0)	62.0 (5.3)	60.0 (8.0)	57.0 (6.0)	63.0 (5.0)	62.0 (5.0)	57.0 (10.0)	64.0 (7.0)
Height^a^, meters, mean (SD)	1.76 (0.07)	1.78 (0.08)	1.78 (0.07)	1.76 (0.07)	1.62 (0.06)	1.63 (0.02)	1.63 (0.06)	1.64 (0.06)	1.62 (0.07)
BMI^a^, kg/m^2^ mean (SD)	27.7 (4.3)	27.5 (4.2)	27.2 (4.2)	26.3 (3.5)	28.2 (6.1)	27.1 (5.5)	26.8 (5.8)	25.3 (4.2)	27.2 (5.8)
Ethnicity, n (%)									
White	4707 (80)	63,452 (89)	176,095 (95)	NA	5475 (72)	65,856 (89)	116,400 (93)	NA	76,213 (84)
Black	1190 (20)	3163 (4)	3961 (2)		2091 (28)	4076 (6)	5582 (5)		10,287 (11)
Other	NA	5012 (7)	5670 (3)		NA	4129 (6)	3443 (3)		4281 (5)
Missing	0 (0)	40 (0)	0 (0)		0 (0)	26 (0)	0 (0)		252 (0)
Smoking, n (%)									
Ever	4349 (74)	45,514 (64)	125,537 (69)	31,224 (67)	3745 (50)	32,856 (44)	67,396 (55)	50,190 (50)	44,146 (49)
Never	1533 (26)	26,141 (37)	55,489 (31)	15,357 (33)	3810 (50)	41,226 (56)	55,789 (45)	50,293 (50)	42,927 (47)
Missing	15 (0)	12 (0)	6502 (3)	459 (1)	11 (0)	5 (0)	3721 (3)	8003 (7)	1219 (1)
Alcohol consumption, units of alcohol per week^a^	4 (9)	9 (26)	8 (20)	15 (19)	1 (4)	9 (26)	3 (8)	7 (9)	2 (5)
Missing	20 (0)	16,463 (22)	0 (0)	20,688 (43)	11 (0)	16,463 (22)	0 (0)	31,496 (29)	47 (0)
HRT, n (%)									
Ever					4109 (54)	49,058 (67)	77,513 (62)	25,884 (28)	54,296 (60)
Never					2242 (35)	24,578 (33)	47,797 (38)	66,873 (72)	36,659 (40)
Missing					1215 (16)	356 (0)	1596 (1)	15,729 (14)	78 (0)

Values in parentheses are percentages unless otherwise stated.

**Abbreviations**: N, number of participants; BMI, body mass index; HRT, hormone replacement therapy; US, United States; UK, United Kingdom; EPIC, European Prospective Investigation into Cancer and Nutrition^b^; NIH-AARP, Diet and Health Study; PLCO, Prostate, Lung, Colorectal, Ovarian Cancer Screening Trial; WHI, Women's Health Initiative; ARIC, Atherosclerosis Risk in Communities study; NA, not applicable.

a These values are based on the index date.

b Denmark, France, Germany, Italy, the Netherlands, Norway, Spain, Sweden and the United Kingdom.

### Associations between overweight years and cancer

Per SD overweight-years, HRs for obesity-related and non-obesity-related cancers in men were 1.15 (95% CI: 1.13, 1.17, I^2^: 0%) and 0.98 (95% CI: 0.95,1.01, I^2^: 70%) respectively. Without lung and prostate cancers, the HR for non-obesity-related cancers in men was 1.04 (95% CI: 1.01, 1.08, I^2^: 34%)] (Table 2). In men, positive associations across overweight-years and single BMI were found for colorectal, pancreatic, kidney and bladder cancer and inverse associations were found for lung cancers (Table 2). In women, HRs per SD overweight-years were 1.08 (95% CI: 1.04, 1.13, I^2^: 83%) for obesity-related cancers and 0.96 (95% CI: 0.92, 1.01, I^2^: 73%) for non-obesity-related cancers; however, without lung cancer, the HR for non-obesity-related cancers was 1.02 (95% CI: 1.00, 1.03, I^2^: 0%). In women, positive associations were found between overweight-years and colorectal, kidney and endometrial cancers. An inverse association was found for lung cancer (Table 2).

**Table 2:** Hazard ratio of cancers per standard deviation of overweight-years and BMI.

Outcomes	Number of cancer events	Overweight-years (per SD)				BMI (per SD)			
		Age-adjusted HR (95% CI)	I^2^	MV-adjusted HR (95% CI)	I^2^	Age-adjusted HR (95% CI)	I^2^	MV-adjusted HR (95% CI)	I^2^
**Men**									
Total Cancers^a^	85,341	1.02 (0.99, 1.05)	0.69	1.01 (0.98, 1.05)	0.78	1.01 (0.99, 1.03)	0.34	1.01 (0.98, 1.04)	0.66
OBR-cancers	12,959	1.15 (1.14, 1.16)	0.00	1.15 (1.13, 1.17)	0.00	1.17 (1.16, 1.18)	0.00	1.16 (1.15, 1.18)	0.00
NOR-cancers	64,743	0.98 (0.97, 1.00)	0.35	0.98 (0.95, 1.01)	0.70	0.98 (0.97, 0.99)	0.00	0.97 (0.96, 0.99)	0.35
NOR-cancers excluding lung and prostate	26,178	1.05 (1.02, 1.07)	0.18	1.04 (1.01, 1.08)	0.34	1.04 (1.02, 1.05)	0.00	1.03 (1.00, 1.07)	0.25
**Specific cancer sites**									
Colorectal	6037	1.14 (1.02, 1.28)	0.66	1.14 (1.02, 1.28)	0.66	1.14 (1.10, 1.18)	0.15	1.14 (1.09, 1.18)	0.21
Pancreas	1957	1.08 (1.04, 1.13)	0.00	1.08 (1.04, 1.12)	0.00	1.07 (1.02, 1.12)	0.00	1.08 (1.02, 1.14)	0.00
Kidney	1967	1.20 (1.08, 1.34)	0.31	1.19 (1.06, 1.33)	0.33	1.25 (1.15, 1.36)	0.19	1.25 (1.15, 1.35)	0.14
Bladder	4018	1.07 (1.02, 1.13)	0.00	1.07 (1.02, 1.13)	0.00	1.08 (1.03, 1.13)	0.00	1.06 (1.01, 1.12)	0.00
Lung	8559	0.94 (0.90, 0.99)	0.37	0.93 (0.87, 0.98)	0.55	0.87 (0.77, 0.98)	0.83	0.85 (0.77, 0.93)	0.82
Prostate	30,006	0.96 (0.90, 1.03)	0.80	0.96 (0.91, 1.02)	0.74	0.97 (0.91, 1.04)	0.85	0.97 (0.92, 1.03)	0.78
**Women**									
Total Cancers^a^	63,732	1.03 (1.01, 1.06)	0.61	1.04 (1.02, 1.06)	0.60	1.04 (1.00, 1.09)	0.83	1.06 (1.01, 1.11)	0.85
OBR-cancers	36,509	1.08 (1.04, 1.13)	0.82	1.08 (1.04, 1.13)	0.83	1.10 (1.07, 1.14)	0.72	1.11 (1.07, 1.15)	0.79
NOR-cancers	24,499	0.94 (0.90, 0.99)	0.75	0.96 (0.92, 1.01)	0.73	0.91 (0.83, 1.01)	0.91	0.94 (0.84, 1.04)	0.90
NOR-cancers excluding lung	16,352	1.00 (0.98, 1.02)	0.00	1.02 (1.00, 1.03)	0.00	0.99 (0.92, 1.05)	0.69	1.01 (0.93, 1.09)	0.65
**Specific cancer sites**									
Colorectal	6251	1.08 (1.05, 1.12)	0.00	1.07 (1.04, 1.10)	0.00	1.09 (1.03, 1.14)	0.47	1.07 (1.03, 1.12)	0.28
Pancreas	2019	1.05 (0.89, 1.24)	0.67	1.03 (0.95, 1.12)	0.47	1.02 (0.95, 1.09)	0.13	1.02 (0.98, 1.06)	0.00
Kidney	1270	1.22 (1.15, 1.29)	0.00	1.20 (1.13, 1.28)	0.00	1.33 (1.13, 1.56)	0.87	1.31 (1.11, 1.54)	0.86
Lung	8114	0.88 (0.84, 0.92)	0.36	0.91 (0.88, 0.94)	0.00	0.85 (0.79, 0.91)	0.67	0.87 (0.81, 0.93)	0.61
Endometrial	3931	1.27 (1.16, 1.39)	0.89	1.28 (1.17, 1.39)	0.80	1.35 (1.18, 1.55)	0.92	1.38 (1.17, 1.64)	0.94
Ovarian	2717	1.00 (0.93, 1.09)	0.52	1.02 (0.94, 1.10)	0.48	0.99 (0.88, 1.11)	0.74	1.00 (0.90, 1.11)	0.67
Post-menopausal breast cancer	17,582	1.00 (0.97, 1.04)	0.46	1.02 (0.98, 1.06)	0.55	1.05 (1.01, 1.10)	0.58	1.07 (1.02, 1.12)	0.58

Multivariable adjusted models: baseline age, ethnicity, alcohol, smoking, HRT.

**Abbreviations**: OBR, obesity-related; NOR, non-obesity-related; CI, confidence interval; HR, hazard ratio; BMI, body mass index; MV, multivariable; SD, standard deviation.

a The sum of OBR and NOR cancer does not equal total cancers as non-melanoma skin cancers were excluded in the EPIC cohort analyses.

**Fig. 2:**
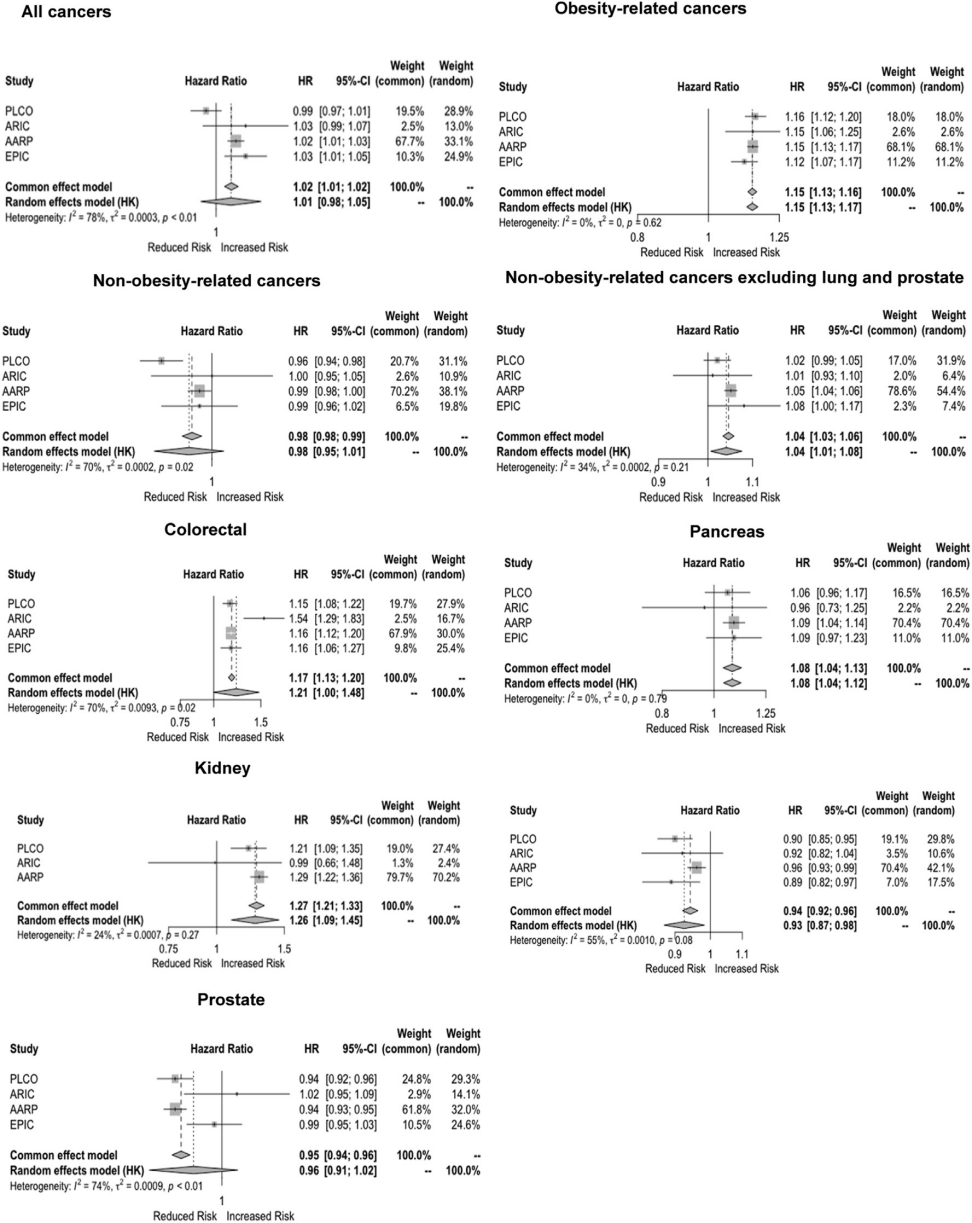
**Forest plots of hazard ratios of cancers per standard deviation overweight-years by cohort in the ABACus 2 Consortium for men.** For each cancer type, there is a separate plot displaying the hazard ratio and 95% confidence interval per standard deviation of overweight years for each cohort, along with the common effects and random effects models from the IPD meta-analysis across the cohorts. Heterogeneity, I^2^ shows the percentage of total variability due to between-study heterogeneity, with values > 50% indicating a high level of heterogeneity. Abbreviations: EPIC, European Prospective Investigation into Cancer and Nutrition; NIH-AARP, NIH-AARP Diet and Health Study; PLCO, Prostate, Lung, Colorectal, Ovarian Cancer Screening Trial; WHI, Women's Health Initiative; ARIC, Atherosclerosis Risk in Communities study. ∗Multivariable adjusted models: baseline age, ethnicity, alcohol, smoking, HRT.

**Fig. 3:**
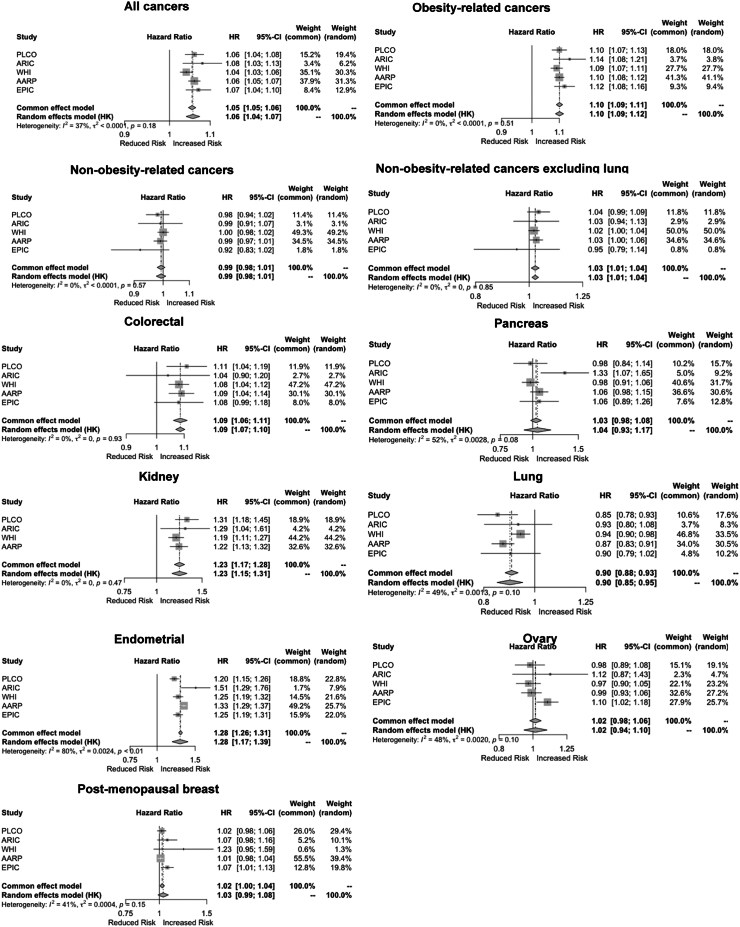
**Forest plots of hazard ratios of cancers per standard deviation overweight-years by cohort in the ABACus 2 Consortium for women**. For each cancer type, each plot displays the hazard ratio and 95% confidence interval per standard deviation of overweight years for each cohort. The common effects and random effects models from the IPD meta-analysis across the cohorts are also shown and the heterogeneity, I^2^, with values >50% indicates a high level of total variability due to between-study differences. Abbreviations: EPIC, European Prospective Investigation into Cancer and Nutrition; NIH-AARP, NIH-AARP Diet and Health Study; PLCO, Prostate, Lung, Colorectal, Ovarian Cancer Screening Trial; WHI, Women's Health Initiative; ARIC, Atherosclerosis Risk in Communities study. ∗Multivariable adjusted models: baseline age, ethnicity, alcohol, smoking, HRT.

Cohort summary associations per 1 SD overweight-years are shown in Fig. 2, Fig. 3. High heterogeneity (I^2^ >50%) across cohorts was found for all cancers, colorectal; lung and prostate cancers in men; and endometrial cancers in women (Figs. 2 and 3). HRs per 100 overweight-years (kg-years/m^2^) and per 5 kg/m^2^ BMI are shown in Table S8.

### Associations between cumulative degree and duration of overweight and cancer

In men, per 1 SD cumulative degree and duration of overweight, HRs for obesity-related cancers were 1.11 (95% CI: 1.04, 1.19, I^2^:81%) and 1.06 (95% CI: 0.94, 1.19, I^2^: 0%) respectively. In men, positive associations were identified for cumulative degree of overweight for bladder cancer and suggestive positive associations for colorectal and pancreatic cancers (Table 3). In women, positive associations were found per SD overweight degree [HR 1.06 (95% CI: 1.03, 1.09, I^2^: 68%)] and duration [HR 1.05 (95% CI: 1.02,1.10, I^2^: 83%)] for obesity-related cancers combined, whereas no associations were found for non-obesity-related cancers. In women, positive associations were found per cumulative overweight degree and/or duration for colorectal, endometrial and ovarian cancers but no evidence of an association was found for postmenopausal breast cancer (Table 3). HRs per 10 unit increase in cumulative overweight degree (kg/m^2^) and per 10-year duration are shown in Table S9.

**Table 3:** Hazard ratios of cancers per standard deviation overweight degree and duration.

Outcomes	Number of cancer events	Degree of Overweight (per SD)				Duration of Overweight (per SD)			
		Age-adjusted HR (95% CI)	I^2^	MV-adjusted HR (95% CI)	I^2^	Age-adjusted HR (95% CI)	I^2^	MV-adjusted HR (95% CI)	I^2^
**Men**									
Total Cancers^a^	85,341	1.02 (0.99, 1.05)	0.74	1.02 (0.98, 1.05)	0.81	1.01 (1.00, 1.02)	0.00	1.00 (0.99, 1.02)	0.00
OBR-cancers	12,959	1.11 (1.04, 1.18)	0.78	1.11 (1.04, 1.19)	0.81	1.08 (0.98, 1.20)	0.81	1.06 (0.94, 1.19)	0.00
NOR-cancers	64,743	0.99 (0.96, 1.03)	0.83	0.99 (0.95, 1.04)	0.88	0.99 (0.96, 1.02)	0.69	0.99 (0.97, 1.01)	0.00
NOR-cancers excluding lung and prostate	26,178	1.04 (1.01, 1.08)	0.33	1.04 (0.99, 1.09)	0.43	1.04 (1.01, 1.07)	0.25	1.01 (0.93, 1.09)	0.00
**Specific cancer sites**									
Colorectal	6037	1.10 (0.98, 1.25)	0.74	1.10 (0.99, 1.23)	0.69	1.06 (0.99, 1.14)	0.64	1.07 (0.99, 1.15)	0.00
Pancreas	1957	1.08 (0.98, 1.17)	0.00	1.07 (0.98, 1.16)	0.00	1.08 (1.02, 1.15)	0.00	1.04 (0.94, 1.15)	0.00
Kidney	1967	1.10 (0.92, 1.31)	0.47	1.10 (0.92, 1.32)	0.48	1.11 (0.69, 1.78)	0.69	0.96 (0.51, 1.78)	0.00
Bladder	4018	1.08 (1.05, 1.11)	0.00	1.09 (1.06, 1.11)	0.00	1.05 (0.97, 1.14)	0.00	1.04 (0.94, 1.14)	0.00
Lung	8559	0.98 (0.85, 1.13)	0.85	0.99 (0.82, 1.19)	0.93	0.93 (0.85, 1.02)	0.73	0.92 (0.81, 1.06)	0.00
Prostate	30,006	0.97 (0.93, 1.01)	0.57	0.96 (0.92, 1.00)	0.53	0.98 (0.95, 1.01)	0.46	0.99 (0.94, 1.06)	0.00
**Women**									
Total Cancers^a^	63,732	1.04 (1.03, 1.05)	0.00	1.04 (1.03, 1.06)	0.02	1.03 (1.00, 1.06)	0.71	1.03 (1.00, 1.07)	0.76
OBR-cancers	36,509	1.05 (1.03, 1.08)	0.50	1.06 (1.03, 1.09)	0.68	1.05 (1.02, 1.08)	0.71	1.05 (1.02, 1.10)	0.83
NOR-cancers	24,499	1.00 (0.92, 1.08)	0.87	1.01 (0.93, 1.10)	0.86	0.99 (0.91, 1.07)	0.86	1.00 (0.93, 1.07)	0.78
NOR-cancers excluding lung	16,352	1.03 (0.95, 1.12)	0.73	1.04 (0.95, 1.14)	0.74	1.04 (0.98, 1.10)	0.61	1.04 (0.99, 1.09)	0.48
**Specific cancer sites**									
Colorectal	6251	1.07 (1.03, 1.11)	0.03	1.07 (1.03, 1.10)	0.00	1.08 (1.02, 1.15)	0.53	1.07 (1.02, 1.14)	0.45
Pancreas	2019	1.03 (0.92, 1.16)	0.52	1.03 (0.92, 1.16)	0.52	1.10 (0.97, 1.24)	0.56	1.10 (0.97, 1.23)	0.55
Kidney	1270	1.13 (0.96, 1.32)	0.67	1.12 (0.95, 1.31)	0.67	1.24 (1.03, 1.50)	0.59	1.22 (1.01, 1.47)	0.59
Lung	8114	0.93 (0.85, 1.00)	0.65	0.95 (0.87, 1.03)	0.68	0.94 (0.85, 1.03)	0.81	0.95 (0.87, 1.05)	0.83
Endometrial	3931	1.21 (1.09, 1.34)	0.76	1.21 (1.10, 1.33)	0.72	1.20 (0.99, 1.46)	0.93	1.19 (0.98, 1.45)	0.93
Ovarian	2717	1.07 (1.02, 1.11)	0.00	1.08 (1.04, 1.12)	0.00	1.02 (0.97, 1.08)	0.00	1.02 (0.97, 1.07)	0.00
Post-menopausal breast cancer	17,582	0.99 (0.94, 1.04)	0.74	1.00 (0.95, 1.06)	0.80	0.99 (0.93, 1.04)	0.82	1.00 (0.95, 1.06)	0.85

Multivariable adjusted models: baseline age, ethnicity, alcohol, smoking, HRT.

Degree of overweight is the cumulative sum of the number of BMI units >25 kg/m^2^.

Duration of overweight is the cumulative sum of the duration overweight (BMI >25 kg/m^2^).

**Abbreviations**: OBR, obesity-related; CI, confidence interval; HR, hazard ratio; BMI, body mass index; MV, multivariable.

a The sum of OBR and NOR cancer does not equal total cancers as non-melanoma skin cancers were excluded in the EPIC cohort analyses.

### Model performance characteristics

Minimal differences in predictive performances across overweight-years, single BMI and combinations of the metrics were found for colorectal, pancreatic, bladder and prostate cancers in men (Table 4). There was no difference in the C-statistic for overweight-years and single BMI for obesity-related cancers in men; however, overweight-years with single BMI both in one model [C-statistic: 0.612 (95% CI: 0.577, 0.646)] marginally outperformed single BMI [0.611 (95% CI: 0.578, 0.644)] (Table S10). Single BMI marginally outperformed overweight-years for kidney and lung cancers in men (Table S10). Cumulative degree and duration of overweight had similar predictive performances across cancer sites except for lung cancer in men where duration [C-statistic: 0.724 (95% CI: 0.696, 0.749)] outperformed the degree of overweight [C-statistic: 0.722 (95% CI: 0.695, 0.748)] (Table 4).

**Table 4:** Comparison of the overweight-years metric and BMI using Harrell's C-statistic.

Characteristic	Harrell's C-statistic (95% CI)					
	Overweight-years	BMI	Difference in c-statistic between BMI and overweight-years	Degree of overweight	Duration of overweight	Difference in c-statistic between duration and degree of overweight
**Men**						
Total Cancers^a^	0.598 (0.565, 0.630)	0.598 (0.564, 0.631)	−0.001 (−0.003, 0.001)	0.599 (0.566, 0.631)	0.598 (0.563, 0.631)	−0.001 (−0.003, 0.001)
OBR-cancers	0.612 (0.578, 0.646)	0.611 (0.578, 0.644)	−0.001 (−0.002, 0.001)	0.613 (0.578, 0.646)	0.609 (0.574, 0.642)	−0.001 (−0.003, 0.001)
NOR-cancers	0.599 (0.563, 0.633)	0.589 (0.563, 0.615)	0.001 (−0.001, 0.002)	0.599 (0.564, 0.634)	0.601 (0.565, 0.636)	0.001 (−0.001, 0.002)
NOR-cancers excluding lung and prostate	0.610 (0.537, 0.677)	0.610 (0.537, 0.677)	0.000 (−0.001, 0.001)	0.609 (0.536, 0.678)	0.609 (0.537, 0.677)	0.000 (−0.001, 0.001)
**Specific cancer sites**						
Colorectal	0.626 (0.583, 0.668)	0.623 (0.587, 0.657)	−0.001 (−0.002, 0.001)	0.626 (0.585, 0.666)	0.621 (0.585, 0.656)	−0.001 (−0.003, 0.001)
Pancreas	0.606 (0.544, 0.666)	0.606 (0.552, 0.659)	−0.001 (−0.003, 0.002)	0.606 (0.541, 0.667)	0.605 (0.549, 0.659)	0.001 (−0.002, 0.004)
Kidney	0.594 (0.568, 0.618)	0.601 (0.579, 0.623)	**0.006 (0.002, 0.011)**	0.594 (0.568, 0.620)	0.599 (0.579, 0.619)	0.005 (−0.000, 0.009)
Bladder	0.677 (0.611, 0.737)	0.678 (0.611, 0.739)	0.000 (−0.005, 0.006)	0.677 (0.611, 0.737)	0.672 (0.608, 0.731)	0.000 (−0.005, 0.006)
Lung	0.722 (0.693, 0.748)	0.726 (0.698, 0.752)	**0.003 (0.002, 0.005)**	0.722 (0.695, 0.748)	0.724 (0.696, 0.749)	**0.003 (0.001, 0.005)**
Prostate	0.597 (0.571, 0.622)	0.596 (0.570, 0.622)	−0.000 (−0.002, 0.001)	0.598 (0.573, 0.622)	0.600 (0.581, 0.619)	−0.001 (−0.002, 0.000)
**Women**						
Total Cancers^a^	0.580 (0.559, 0.601)	0.582 (0.561, 0.602)	0.002 (−0.005, 0.008)	0.577 (0.557, 0.596)	0.577 (0.562, 0.592)	−0.002 (−0.008, 0.004)
OBR-cancers	0.566 (0.534, 0.598)	0.573 (0.546, 0.600)	0.003 (−0.003, 0.009)	0.564 (0.535, 0.593)	0.558 (0.537, 0.579)	−0.001 (−0.006, 0.004)
NOR-cancers	0.639 (0.579, 0.695)	0.641 (0.578, 0.699)	0.002 (−0.001, 0.005)	0.638 (0.579, 0.693)	0.639 (0.580, 0.695)	−0.000 (−0.002, 0.002)
NOR-cancers excluding lung	0.589 (0.542, 0.634)	0.590 (0.542, 0.635)	0.001 (−0.002, 0.003)	0.589 (0.542, 0.634)	0.588 (0.542, 0.633)	−0.001 (−0.003, 0.000)
**Specific cancer sites**						
Colorectal	0.627 (0.584, 0.668)	0.629 (0.590, 0.666)	−0.001 (−0.002, 0.000)	0.628 (0.585, 0.669)	0.629 (0.589, 0.667)	−0.000 (−0.002, 0.001)
Pancreas	0.634 (0.582, 0.682)	0.633 (0.581, 0.682)	0.001 (−0.001, 0.003)	0.634 (0.584, 0.682)	0.632 (0.584, 0.678)	−0.001 (−0.004, 0.003)
Kidney	0.607 (0.586, 0.627)	0.626 (0.606, 0.645)	**0.014 (0.002, 0.026)**	0.608 (0.588, 0.628)	0.622 (0.599, 0.644)	**0.015 (0.004, 0.025)**
Lung	0.743 (0.697, 0.784)	0.743 (0.697, 0.784)	0.002 (−0.002, 0.005)	0.743 (0.694, 0.787)	0.743 (0.699, 0.781)	−0.001 (−0.003, 0.002)
Endometrial	0.613 (0.563, 0.661)	0.625 (0.584, 0.663)	0.011 (−0.001, 0.023)	0.617 (0.572, 0.659)	0.611 (0.566, 0.654)	−0.004 (−0.012, 0.004)
Ovarian	0.583 (0.540, 0.624)	0.582 (0.541, 0.622)	−0.001 (−0.005, 0.003)	0.583 (0.541, 0.625)	0.584 (0.545, 0.623)	−0.002 (−0.005, 0.001)
Post-menopausal breast cancer	0.598 (0.511, 0.678)	0.603 (0.509, 0.690)	0.002 (−0.000, 0.005)	0.596 (0.514, 0.672)	0.595 (0.513, 0.672)	−0.001 (−0.003, 0.001)

Key: Bold—significant difference in C-statistic.

All models were multivariable adjusted, including baseline age, ethnicity, alcohol, smoking, HRT.

**Abbreviations**: SE, standard error; OBR, obesity-related; BMI, body mass index; CI, confidence interval; MV, multivariable-adjusted.

a The sum of OBR and NOR cancer does not equal total cancers as non-melanoma skin cancers were excluded in the EPIC cohort analyses.

In women, no notable differences in C-statistics across overweight-years, single BMI and combinations of the metrics were found for combined obesity-related cancers combined, and colorectal, pancreatic, lung, endometrial, ovarian and postmenopausal breast cancers (Table 4). Single BMI [C-statistic: 0.626 (95% CI: 0.606, 0.646)] and overweight-years with single BMI [C-statistic: 0.626 (95% CI: 0.600, 0.650)] both outperformed overweight-years [C-statistic: 0.607 (95% CI: 0.586, 0.627)] for kidney cancers in women (Table 4, Table S10). No significant differences were found between cumulative overweight degree and duration in women except for kidney cancer where duration of overweight [C-statistic:0.622 (95% CI: 0.599, 0.644)] outperformed the degree of overweight [C-statistic: 0.608 (95% CI: 0.588, 0.628)].

Obese-years had similar findings as overweight-years (Tables S11–S15) except single BMI had a higher C-statistic than obese-years for lung cancer in men and obesity-related cancers, kidney, endometrial and postmenopausal breast cancers in women (Table S15).

### Sensitivity analysis

Findings from analyses only including participants with ≥3 observed BMI records (Tables S16–S25) were primarily in line with main analyses confirming the reliability of BMI predictions. However, C-statistics for overweight-years adjusted for single BMI were higher than overweight-years for obesity-related and non-obesity-related cancers, colorectal and lung cancers in men and obesity-related cancers combined, kidney, endometrial and postmenopausal breast cancers in women (Table S20). Single BMI had a higher C-statistic than overweight-years for obesity-related cancers in men and kidney, endometrial and postmenopausal breast cancers in women (Table S20). Analysis of BMI predictions with the subgroup with ≥1 BMI measurement confirmed our main analysis indicating the lack of selection bias towards healthier individuals (Table S26, Table S35).

## Discussion

In this IPD meta-analysis, longer duration and higher degree of overweight quantified using overweight-years were associated with increased risk of colorectal, kidney, bladder and pancreatic cancers in men and colorectal, kidney, and endometrial cancer in women. Overweight-years was positively associated with non-obesity-related cancers after excluding cancers of the lung and prostate (among men). Associations were present for cumulative degree and duration of overweight separately. This may suggest that longer duration/degree of adiposity could be related to more cancers than anticipated. However, it is important to consider the possibility of residual confounding by other factors such as smoking or socioeconomic status.^35^ This study focused on improving obesity-related cancer prediction. Our hypothesis was not upheld; overweight-years made little improvement in the predictive ability of cancer incidence than single BMI.

Recalde et al. (2023), analysed six longitudinal exposures (i) duration of excess BMI (≥25 kg/m^2^), (ii) cumulative exposure to excess BMI (≥25 kg/m^2^), (iii) age of onset of excess BMI ≥ 25 kg/m^2^, and mutually adjusted models for (i) and (iii).^9^ Whilst we acknowledge that other metrics are available in the literature beyond those listed, our pre-specified research question was centred on the overweight-years metric as described by Abdullah et al., in 2014 in the cardiovascular literature.^4^ Arnold et al. (2016) analysed overweight-years and cancer risk in 329,576 participants for obesity-related cancers, colorectal and breast cancers whereas we analysed obesity-related and non-obesity-related cancers and cancer sites separately where the sample size allowed.^5^ Our study provides further evidence that overweight duration is positively associated with obesity-related cancers.^5^ Degree and duration of excess BMI over adulthood showed strong positive associations with cancer incidence and is in line with other studies that analysed overweight- and obese-years.^5^^,^^6^

In men, associations between cumulative degree of overweight for bladder cancer, and suggestive positive associations for colorectal and pancreatic cancers. A case–control study of 5635 participants in Germany found HRs of higher magnitude between per SD overweight-years and colorectal cancer than BMI whereas our study formally compared and found no difference in the predictive performance across metrics.^8^ For pancreatic cancer, a Canadian case–control study suggested a life course approach to disease risk given associations between early adulthood overweight trajectories and pancreatic cancer risk.^36^ For adiposity-related bladder cancer risk the literature remains inconsistent.^37^^,^^38^ We found positive associations between overweight-years and cumulative degree of overweight for bladder cancer in men. We cannot rule out whether associations were due to residual confounding by smoking which is a risk factor for bladder cancer. Analysis of 23,378,895 individuals in Korea found BMI had a positive association with bladder cancer independent of smoking.^39^ A prior EPIC study found positive associations between BMI and bladder cancer among men but not women; however, positive associations were not found among never smokers thus indicating residual confounding by smoking.^35^ A Mendelian randomisation study supports positive associations between BMI and bladder cancer.^40^ However, there may be ascertainment bias given prior routine healthcare urine checks so findings may be incidental.^41^

In women, we found positive associations between cumulative degree and/or duration of overweight and colorectal, kidney, endometrial, and ovarian cancer. In terms of endometrial cancer, our study findings confirm a study in Icelandic women which found associations between obese-years and endometrial and postmenopausal breast cancers.^7^ However, no evidence of an association was found in our study for postmenopausal breast cancer. Although the link between excess BMI and these cancers are known,^2^ understanding of associations between cumulative overweight degree and duration separately is limited. Positive associations found per SD cumulative overweight degree and duration could be explained by underlying biological mechanisms such as chronic inflammation, oestrogen levels and oxidative DNA damage.^42^ Cumulative adiposity exposure could have posed a greater risk of insulin resistance which is a plausible biological explanation for the increased risk of cancer.^43^ On a public health level, the message regarding risks associated with the duration of living with overweight is not as clear-cut. Focus on cancer epidemiology has primarily been on risks associated with the degree of overweight using single BMI. Our findings emphasise the importance of also minimising overweight duration.

To our knowledge, this is the largest IPD meta-analysis on cumulative excess BMI and cancer risk which covers large-scale populations from the US and 9 European countries. This increased the statistical power thus validity and precision of summary estimates. Random effects IPD meta-analysis accounted for between-study variation on a cohort level and participant-level variation.^44^ BMI variability by sex was considered.^45^ Another strength was the relative novelty in the application of overweight- and obese-years in cancer literature. A large number of participants was investigated given the number of cohorts recruited, and multiple imputation handled missing data; both of these increased the statistical power, thus validity and precision of summary estimates. Most observational time-to-event studies in oncology lack of use of multiple imputation methods.^46^ More cancer sites were analysed separately for associations with cumulative degree and duration of overweight which helped identify whether there are cancer site-specific variations in underlying biological mechanisms. Another strength was that cumulative degree and duration were analysed separately as overweight- and obese-years are a composite measure and do not necessarily demonstrate the same magnitude and direction of associations with overweight degree and duration.

49% of the ABACus 2 consortium were excluded from this study which is a limitation of the study: with large numbers being excluded due to the requirement for at least 3 BMI measurements and the lack of availability of data on 70,889 EPIC participants from Greece. However, a sensitivity analysis using participants with at least 1 BMI measurement demonstrated that for the characteristics examined, there were no striking differences in the sensitivity findings compared with the main analysis. Duration of excess BMI may be underestimated given excess BMI exposure in childhood, adolescence and beyond the start of follow-up were not accounted for due to i) the lack of BMI records during such ages and ii) the nature of analysis comparing metrics with single BMI assessed at the same time. Additionally, there was a potential underestimation of the degree of overweight over time in our study taking into consideration prior studies.^47^^,^^48^ Some cohorts had self-reported weight which may have introduced measurement error which increased with age as height was assumed to have remained constant across all ages.^49^ Marginal differences were apparent when comparing the performance characteristic of overweight- and obese-years with once-only BMI. There are several potential reasons for such findings including (i) the likely underestimation of the degree and duration of cumulative exposure to excess BMI; (ii) cumulative degree and duration of excess adiposity may not be of equal weighting unlike that assumed in the overweight- and obese-years metrics, or lastly (iii) our hypothesis may simply not be true–longitudinal changes in BMI may not be predictive of adiposity-related cancer risk compared with a single BMI measure. BMI measurements collected from each cohort (except ARIC) were far apart so local changes in weight (intentional or unintentional) were not included in the BMI prediction model. Consequently, the findings of the ARIC study alone have been published separately.^50^ Generally, it is important to acknowledge the potential competing risk of death as a limitation of this study as well as the potential residual confounding by smoking.

Future work on other populations will identify whether findings regarding the cancer risks associated with cumulative over-weight years and whether the similarities in the performance characteristics of overweight-years and single BMI are similar. There is limited cancer research in African, Asian and Middle Eastern cohorts despite the increase in prevalence of obesity over time, and the differences in the percentage of body fat for a given BMI among persons of different ancestries.^51^ Although the included cohorts used had long durations of follow-up and an array of covariates, analysing more recent studies may be representative of adiposity in current populations. Given sample size requirements, not all cancer sites were analysed but could be using a one-stage IPD meta-analysis. There are metrics beyond overweight-years that explore associations with cancer risk such as the age of onset of excess BMI recently explored by Recalde et al. (2023)^9^ which require further exploration in populations beyond those previously explored. Additionally, the timing of excess BMI across all ages may be relevant in cancer development which will be further explored in the ABACus 2 project.^52^ This study focuses on late adulthood, but findings may differ if a single BMI in early adulthood is used to predict cancer risk before age 30.^53^ BMI has limited generalisability across race and sex^54^; therefore, exploring metrics like waist circumference, waist-to-hip ratio, body fatness percentage and magnetic resonance determined adipose measures may be useful.^55^ Future work could explore underlying mechanisms and causal links between duration and degree of excess BMI and cancer. Additionally, future analysis may include the influence of weight loss interventions after particular durations or degrees of adiposity exposure to identify whether adiposity-related cancer risks can be reversed.

Overall, overweight-years had similar cancer-predictive characteristics to single BMI. Higher duration and degree of overweight were associated with some cancers. Minimising adiposity, including the degree and duration, should be considered in cancer policy and prevention strategies.

## Contributors

NKH, GPM, MS and AGR designed the research study (conceptualisation, methodology, project administration, funding acquisition). NKH conducted the formal analysis and GPM and MS accessed and verified the data. NH wrote the original draft, which was then critically reviewed by all authors (MS, GPM, RS, CEM, FR, AT, AKH, MLN, CEJ, EAP, HF, MJG, AGR). All authors agreed on the submission of the manuscript.

## Data sharing statement

Data availability will be shared subject to proposal approval, with a signed data access agreement and individual cohort review committee approval. Applications to access data from each cohort can be made via the following links:

- ARIC: https://sites.cscc.unc.edu/aric/distribution-agreements.

- EPIC: https://epic.iarc.fr.

- PLCO: https://cdas.cancer.gov/plco/.

- WHI: https://www.whi.org/md/working-with-whi-data.

- NIH-AARP: https://www.nihaarpstars.com/Default.aspx?projectid=098b1a48-4822-4126-8d09-562e7d3b3659.

## Declaration of interests

NH, MS, GPM, RS, CEM, FR, AT, AKH, HF, MJG, and AGR declare no competing interests.

EAP received a National Institutes of Health research grant; an award made to Johns Hopkins University for the manuscript.

MN received funding for the paper from the National Heart Lung and Blood Institute, National Institutes of Health to the Institution (Fred Hutchinson Cancer Center). MN receives funding as the Deputy Editor of, Journal of Nutrition.

CJ received the American Cancer Society's support for the present manuscript and the following grants or contracts to the institution, Johns Hopkins University, from the National Institutes of Health, Prostate Cancer Foundation, The Ralph Lauren Corporate Foundation, Department of Defense.
