# Supplementary material for: Excess weight by degree and duration and cancer risk (ABACus2 consortium): a cohort study and individual participant data meta-analysis
Source: eClinicalMedicine. 2024 Nov 19;78:102921. doi: 10.1016/j.eclinm.2024.102921 (PMC11617392; doi:10.1016/j.eclinm.2024.102921)
Supplement: Supplementary Figure and Tables [file mmc1.pdf]

Supplementary

(a) Recall study

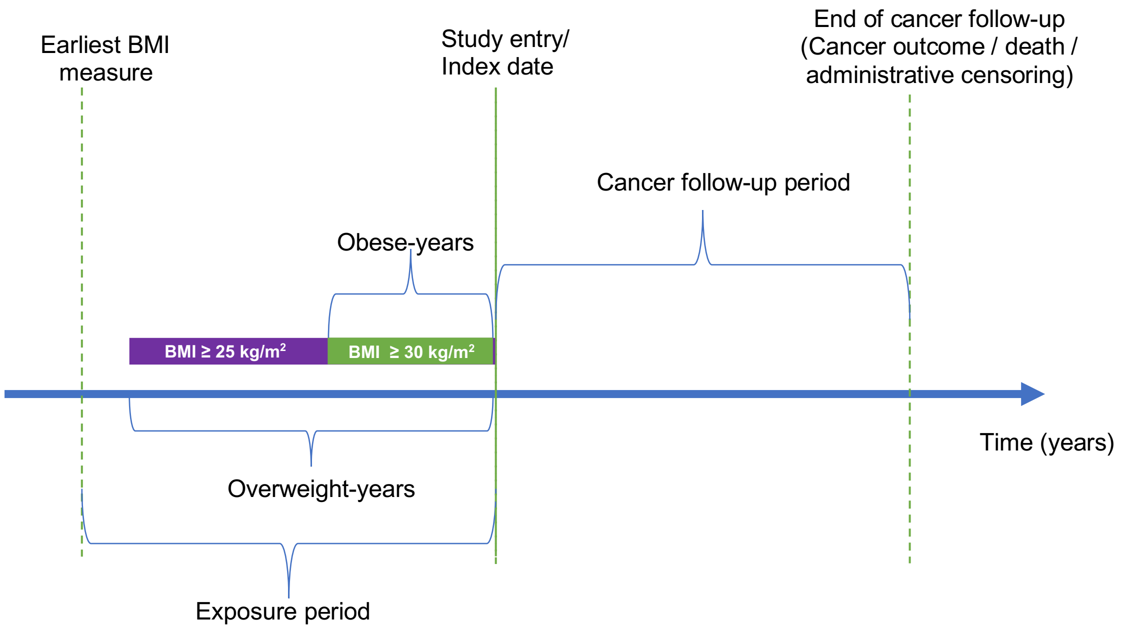

(b) Prospective study

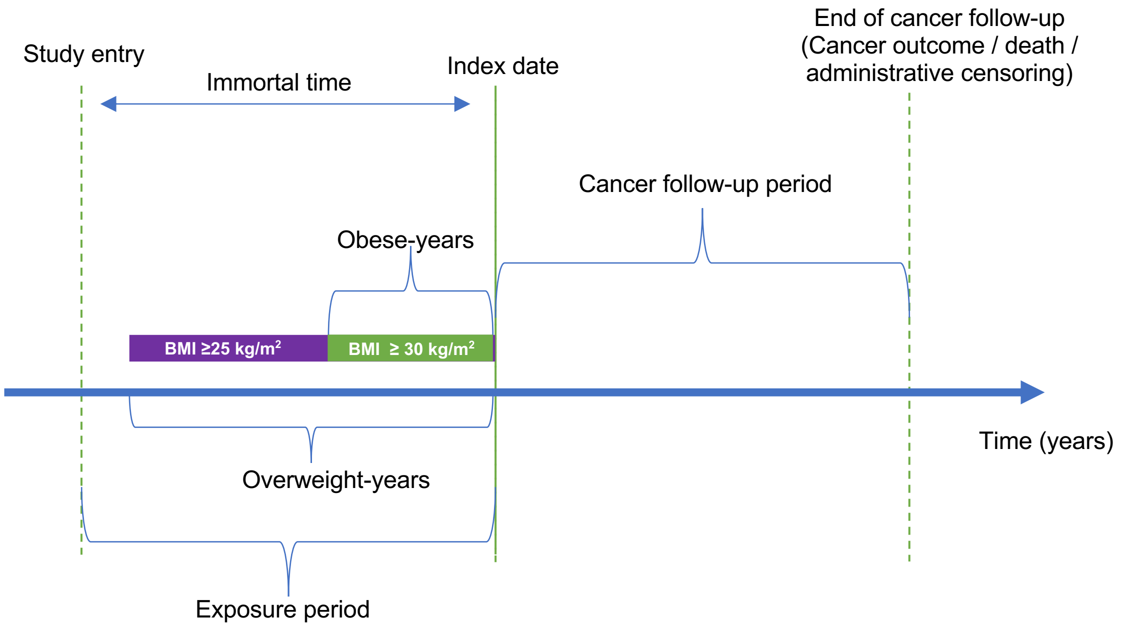

Figure S1: Diagram of the exposure and follow-up period for a (a) recall and (b) prospective study. *In the exposure period, overweight-years were calculated for any BMI readings above 24.9 kg/m*^{2} *including those over 30 kg/m*^{2}*. Obese-years were calculated for any BMI readings above 29.9 kg/m*^{2}*. The sum of the overweight- or obese-year exposures before and at the index date and the single BMI measure at the index date were used as excess adiposity exposures. The follow-up period in this study ended with a cancer diagnosis, death or administrative censoring.*

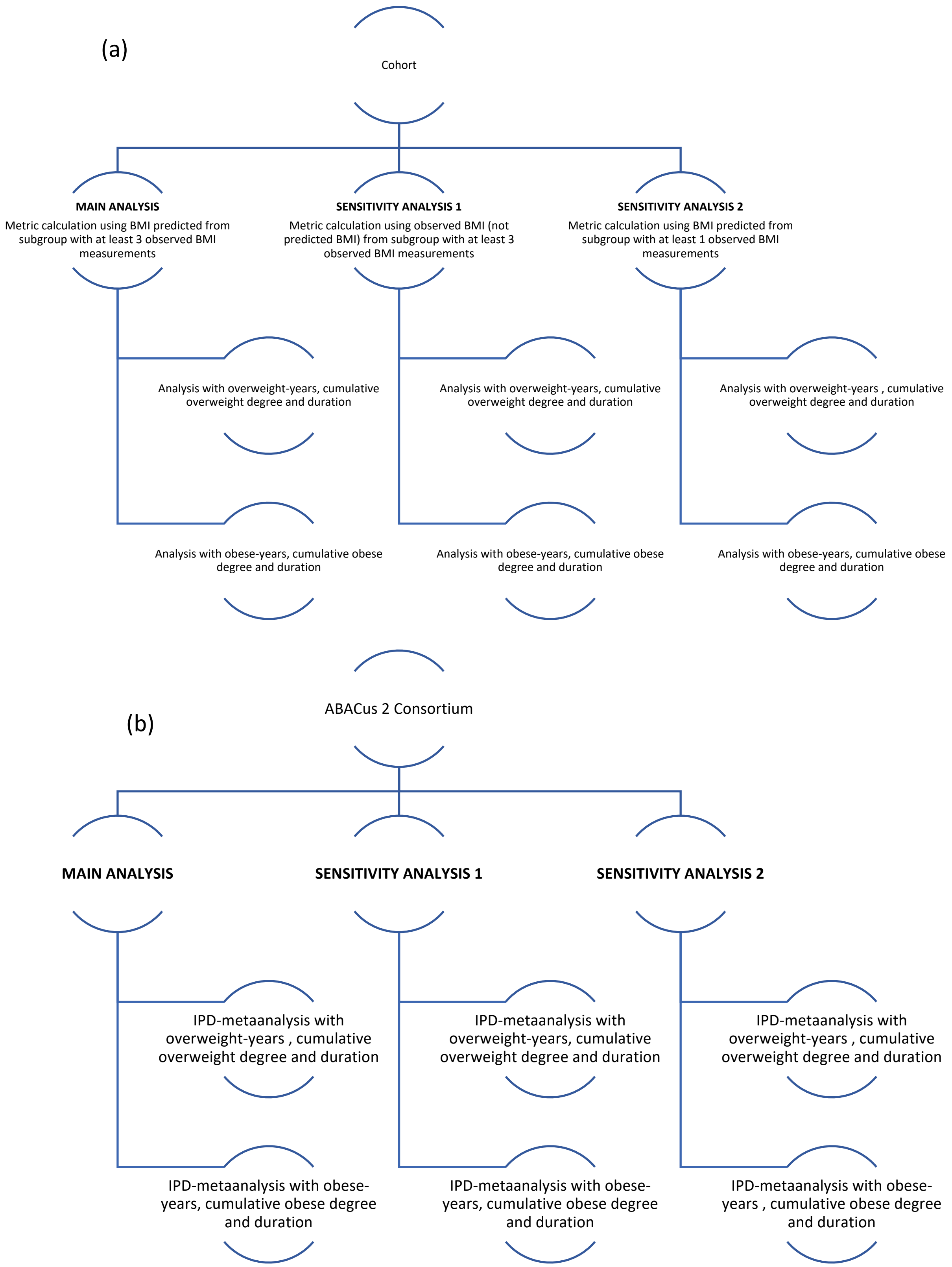

Figure S2: Diagram of steps within 2-stage IPD metanalysis: a) individual study analysis undertaken and b) random effects IPD-metanalysis of the ABACus 2 Consortium.

Table S1: Datasets included in the ABACus 2 Consortium.

Study	Country	Study entry date	N	BMI and WC data collection	Disease outcomes	Method of Number of cancer events follow-up
**ARIC (1)**	US	1987	15,792	Prospective weight and height collection by trained personnel using standardized protocols across centers. Weight, height and WC were collected at Visit 1-4 each 3 years apart and then 15 years later, measures were collected more frequently at Visit 5-8 (1). BMI at age 25 was recalled.	Cancer incidence. Administrative censoring on December 31, 2015.	Linkage to 4 state cancer registries (ICD-O3 codes) supplemented with hospital discharge summaries and medical records.
**EPIC (2)**	(10 European countries*)	1990	521,000	BMI and WC data were collected at study entry and BMI at age 20 was recalled. 5 years post study entry, BMI and WC were collected by a questionnaire. Details on data collection by country have been described in-depth elsewhere (3).	Cancer Incidence. Administrative censoring was center specific (22 collaborating centres).	Registry linkage (ICD-10 cancer codes) except in Germany and France, where cancer pathology registries, active-follow-up and health insurance records were used.
**WHI (3) Women only**	US	1991	161,808	At baseline, recall self-reports of BMI at ages 18, 35 and 50 were collected. Height and weight measures were also collected at baseline (5).	Cancer incidence. Administrative censoring for main study 2005, Extension 1 in 2010 and Extension 2 in 2020.	Annual mailed follow-up questionnaires. Cancer (ICD-10 codes).
**PLCO (4)**	US	1993	154,887	Recall BMI at ages 20 and 50 and self-reported BMI at study entry (mean age 63 years) were collected (7).	Cancer incidence. Administrative censoring in 2009.	Self-reported annual follow-up questionnaires. Cancer codes (ICD-0-3 codes).
**NIH-AARP (5)**	US	1996	566,398	Recall BMI at ages 18, 35 and 50 were collected by a questionnaire and BMI at study entry (mean age 61) was self-reported.	Cancer incidence. Administrative censoring in 2011.	Cancer registry linkage (ICD-0-3 codes) in the original 8 states of recruitment.

Absolute criterion: over three measures of BMI over time

*** Denmark, France, Germany, Italy, the Netherlands, Norway, Spain, Sweden and the United Kingdom.**

Abbreviations: N, number of participants; BMI, body mass index; WC, waist circumference; US, United States; UK, United Kingdom; EPIC, European Prospective Investigation into Cancer and Nutrition; NIH-AARP, NIH-AARP Diet and Health Study; PLCO, Prostate, Lung, Colorectal, Ovarian Cancer Screening Trial; WHI, Women's Health Initiative; ARIC, Atherosclerosis Risk in Communities study.

1. Joshu CE, Barber JR, Coresh J, Couper DJ, Mosley TH, Vitolins MZ, et al. Enhancing the infrastructure of the atherosclerosis risk in Communities (ARIC) study for cancer epidemiology research: Aric cancer. Cancer Epidemiol Biomarkers Prev. 2018 Mar 1;27(3):295–305.
2. Riboli E, Kaaks R. The EPIC Project: Rationale and study design. Int J Epidemiol. 1997;26(SUPPL. 1).
3. Anderson G, Cummings S, Freedman LS, Furberg C, Henderson M, Johnson SR, et al. Design of the Women's Health Initiative clinical trial and observational study. Control Clin Trials. 1998;19(1):61–109.
4. Black A, Huang W-Y, Wright P, Riley T, Mabie J, Mathew S, et al. PLCO: Evolution of an Epidemiologic Resource and Opportunities for Future Studies. Rev Recent Clin Trials. 2015 Oct 7;10(3):238–45.
5. Schatzkin A, Subar AF, Thompson FE, Harlan LC, Tangrea J, Hollenbeck AR, et al. Design and serendipity in establishing a large cohort with wide dietary intake distributions: The National Institutes of Health-American Association of retired persons diet and health study. Am J Epidemiol. 2001 Dec 15;154(12):1119–25.

Table S2: Overweight-years calculation example from an individual in the PLCO cohort.

Examination	Interval	Age (Years)	BMI	Degree of overweight	Duration of overweight	Overweight-years	Cumulative overweight-years	Cumulative excess overweight degree	Cumulative excess overweight duration
1	NA	54	24.20	0.00	0.00	0.00	0.00	0.00	0.00
2	2	56	26.40	1.50	0.00	0.00	0.00	1.50	0.00
3	3	59	26.00	1.10	3.00	4.50	4.50	2.60	3.00
4	5	64	28.50	3.60	5.00	5.50	10.00	6.20	8.00

Example calculation of overweight-years of examination 3 = prior degree of overweight (1.5) x duration of overweight for examination 3 (3) = (1.5 x 3) = 4.5 overweight-years.

Abbreviation: BMI, body mass index.

NB: This is only a simplified example calculation of the metric. .

Table S3: Obese-years calculation example from an individual in the PLCO cohort.

Examination	Interval	Age (Years)	BMI	Degree of obesity	Duration of obesity	Obese-years	Cumulative obese-years	Cumulative excess obesity degree	Cumulative excess obesity duration
1	NA	20	29.80	0.00	0.00	0.00	0.00	0.00	0.00
2	1	21	30.47	0.57	0.00	0.36	0.36	0.57	0.00
3	1	22	30.68	0.78	1.00	0.57	0.94	1.35	1.00
4	1	23	30.89	0.99	1.00	0.78	1.72	2.34	2.00

Example calculation of obese-years of examination 3 = prior degree of obesity (0.57) x duration of obesity (1) = (0.57x1) = 0.57 obese-years.

Abbreviation: BMI, body mass index.

NB: This is only a simplified example calculation of the metric. .

Table S4: Mean exposure to excess BMI at baseline and cancer follow-up.

Characteristic	ARIC	PLCO	NIH-AARP	EPIC	WHI
	Men				
**Baseline BMI, kg/m^2**	27.70 (4.30)	27.50 (4.20)	27.20 (4.20)	26.30 (3.50)	
**End of cancer follow up, years**	18.00 (8.00)	10.40 (4.00)	6.70 (4.70)	9.30 (3.50)	
**Incident cancers, N**	2,072	16,790	58,840	7,639	
**Total cumulative overweight years**	50.00 (65.00)	57.00 (81.00)	52.00 (77.00)	44.00 (65.00)	
**Total cumulative overweight degree**	53.00 (67.00)	60.00 (84.00)	55.00 (80.00)	46.00 (67.00)	
**Total cumulative overweight duration**	16.00 (12.00)	20.00 (16.00)	19.00 (16.00)	19.00 (16.00)	
**Total cumulative obese-years**	8.00 (26.00)	8.00 (32.00)	7.00 (30.00)	4.00 (20.00)	
**Total cumulative obese-degree**	9.00 (27.00)	9.00 (33.00)	8.00 (32.00)	4.00 (21.00)	
**Total cumulative obese-duration**	3.00 (7.00)	3.00 (8.00)	3.00 (8.00)	2.00 (6.40)	
	Women				
**Baseline BMI, kg/m^2**	28.20 (6.10)	27.10 (5.50)	26.80 (5.80)	25.30 (4.20)	27.20 (5.80)
**End of cancer follow up, years**	20.00 (7.00)	10.80 (3.40)	6.40 (4.40)	8.70 (3.50)	18.20 (5.90)
**Incident cancers**	1,804	10,917	29,836	10,129	11,046
**Total cumulative overweight years**	84.00 (100.00)	42.00 (83.00)	41.00 (83.00)	28.00 (61.00)	103.00 (132.00)
**Total cumulative overweight degree**	89.00 (104.00)	44.00 (87.00)	44.00 (87.00)	29.00 (63.00)	107.00 (136.00)
**Total cumulative overweight duration**	19.00 (13.00)	12.00 (14.00)	12.00 (14.00)	10.00 (14.00)	28.00 (18.00)
**Total cumulative obese-years**	24.00 (55.00)	10.00 (40.00)	10.00 (40.00)	4.00 (23.00)	25.00 (72.00)
**Total cumulative obese-degree**	26.00 (57.00)	11.00 (43.00)	11.00 (43.00)	5.00 (25.00)	26.00 (75.00)
**Total cumulative obese-duration**	7.00 (10.00)	3.00 (7.00)	3.00 (7.00)	1.60 (5.70)	7.00 (13.00)

Mean (SD) at baseline.

Abbreviations: N = number of participants; SD, standard deviation; BMI, body mass index

Table S5: All-cause and cancer mortality in the original cohorts, main study and sensitivity analyses.

Cohorts	Mortality			Cancer Mortality		
	Original cohort	≥ 3BMI	≥ 1BMI	Original cohort	≥ 3BMI	≥ 1BMI
	**Men**					
**ARIC**	4,281 (61)	3,475 (59)	3,648 (58)	1,079 (15)	866 (15)	910 (15)
**PLCO**	27,115 (34)	25,432 (35)	25,643 (36)	8,270 (11)	7,752 (11)	7,806 (11)
**NIH-AARP**	194,765 (57)	105,150(56)	186,079 (57)	26,588 (8)	14,519 (8)	25,049 (8)
**EPIC**	21,424 (16)	6,784 (14)	10,739 (11)	1,979 (2)	629 (1)	944 (1)
	**Women**					
**ARIC**	4,077 (47)	3,407 (45)	3,469 (45)	889 (10)	714 (9)	728 (9)
**PLCO**	17,600 (23)	16,520 (22)	16,743 (22)	5,591 (8)	5,258 (7)	5,319 (7)
**NIH-AARP**	106,872 (47)	57,283 (45)	100,597 (47)	14,996 (7)	8,226 (6)	13,925 (6)
**EPIC**	25,212 (8)	6,239 (6)	10,364 (5)	2,161 (1)	520 (1)	779 (1)
**WHI**	66,068 (41)	38,261 (42)	65,277 (41)	9,655 (6)	5,680 (6)	9,529 (5)

Values in parentheses are percentages of the subgroup listed unless stated otherwise.
Abbreviations: N, number of participants; BMI, body mass index; US, United States; UK, United Kingdom; EPIC, European Prospective Investigation into Cancer and Nutrition[1]; NIH-AARP, Diet and Health Study; PLCO, Prostate, Lung, Colorectal, Ovarian Cancer Screening Trial; WHI, Women's Health Initiative; ARIC, Atherosclerosis Risk in Communities study; NA, not applicable. [1]Denmark, France, Germany, Italy, Netherlands, Norway, Spain, Sweden and the United Kingdom.

Table S6: Characteristics of those included and excluded from this study.

Characteristic	Men							
	ARIC		PLCO		NIH-AARP		EPIC	
	Included N = 5,897	Excluded N = 1,173	Included N=71,667	Excluded N = 4,197	Included N = 187,528	Excluded N=152,138	Included N = 47,040	Excluded N = 84,385
**Age *, years, mean (SD)**	57.0 (6.0)	57.2 (5.8)	63.0 (5.0)	64.0 (6.0)	62.0 (5.3)	62.0 (5.4)	60.0 (8.0)	57.0 (9)
**Missing**	0	678	0	2,572	0	0	0	0
**Height*, meters, mean (SD)**	1.76 (0.07)	1.77 (0.07)	1.78 (0.08)	1.78 (0.08)	1.78 (0.07)	1.78 (0.08)	1.76 (0.07)	1.74 (0.07)
**Missing**	0	10	0	3,289	0	3,842	0	32,386
**BMI at baseline*, kg/m^2 mean (SD)**	27.7 (4.3)	27.4 (4.4)	27.5 (4.2)	27.0 (5.0)	27.2 (4.2)	27.4 (4.5)	26.3 (3.5)	27.0 (3.6)
**Missing**	0	693	0	3,768	0	6,580	0	32,386
**Race**								
**White**	4,707 (80)	710 (61)	63,452 (89)	1,269 (78)	176,095 (95)	138,213 (91)	NA	
**Black**	1,190 (20)	440 (38)	3,163(4)	153 (9)	3,961 (2)	5,343 (4)		
**Other**	NA	23 (2)	5,012(7)	199 (5)	5,670 (3)	6,251(4)		
**Missing**	0	0	40	2,576	0	2,331		
**Smoking**								
**Ever**	4,349 (74)	351 (30)	45,514 (64)	998 (62)	125,537 (69)	102,629(7)	31,224 (67)	31,468(64)
**Never**	1,533 (26)	141 (12)	26,141 (36)	618 (15)	55,489 (31)	42,410(28)	15,357 (33)	17,359(36)
**Missing**	15	681	12	2,581	6,502	7,099	459	35,558
**Alcohol consumption, units per week***	4 (9)	4 (8)	9 (26)	10 (13)	8 (20)	2 (5)	15 (19)	21 (22)
**Missing**	20	683	16,463	1,989	0	0	20,688	43,122

	Women									
	ARIC		PLCO		NIH-AARP		EPIC		WHI	
	Included N = 7,566	Excluded N = 1,121	Included N=74,087	Excluded N = 3,497	Included N = 126,906	Excluded N = 99,826	Included N=108,486	Excluded N =210,200	Included N=91,033	Excluded N =70,752
**Age *, years, mean (SD)**	57.0 (6.0)	57.0 (6.0)	63.0 (5.0)	64.0 (6.0)	62.0 (5.0)	62.0 (5.0)	57.0 (10.0)	60.0 (10.0)	64.0 (7.0)	63.0 (7.0)
**Missing**	0	766	0	1,893	0	0	0	0	0	3
**Height*, meters, mean (SD)**	1.62 (0.06)	1.62 (0.06)	1.63 (0.02)	1.63 (0.08)	1.63 (0.06)	1.63 (0.07)	1.64 (0.06)	1.62 (0.07)	1.62 (0.07)	1.61 (0.07)
**Missing**	0	9	0	2,385	0	2,416	0	0	0	891
**BMI at baseline*, kg/m^2 mean (SD)**	28.2 (6.1)	28.9 (7.0)	27.1 (5.5)	29.0 (9.0)	26.8 (5.8)	27.0 (6.5)	25.3 (4.2)	25.2 (4.4)	27.2 (5.8)	28.9 (5.9)
**Missing**	0	779	0	2,980	0	7,364	0	79,274	0	1,393
**Race**										
**White**	5,475 (72)	558 (50)	65,856 (89)	1,197 (75)	116,400 (93)	86,206(86)	NA		76,213 (84)	57,305(81)
**Black**	2,091 (28)	538 (48)	4,076(6)	222 (6)	5,582(5)	7,109 (7)			10,287 (11)	9,234 (13)
**Other**	NA	25 (2.2)	4,129 (6)	184(5)	3,443 (3)	4,335 (4)			4,281 (5)	4,052 (6)
**Missing**	0	0	26	1,894	0	2,176			252	161
**Smoking**										
**Ever**	3,745 (50)	169 (15)	32,856 (44)	705 (44)	67,396 (55)	52,547(53)	50,190 (50)	47,195(40)	44,146 (49)	34,093(49)
**Never**	3,810 (50)	182 (16)	41,226 (56)	895 (26)	55,789 (45)	42,802(43)	50,293 (50)	71,351 (60)	42,927 (47)	35,752 (51)
**Missing**	11	770	5	1,897	3,721	4,477	8,003	91,654	1,219	907
**Alcohol consumption, units per week***	1 (4)	1 (3)	9 (26)	3 (12)	3 (8)	1 (2)	7 (9)	8 (12)	2 (5)	2 (4)
**Missing**	11	771	16,463	1,543	0	0	31,496	93,273	47	87
**HRT**										
**Ever**	4,109 (54)	81 (7)	49,058 (67)	818 (54)	77,513 (62)	5,549 (50)	25,884 (28)	21,517(18)	54,296 (60)	36,480(52)
**Never**	2,242 (35)	206 (18)	24,578 (33)	704 (20)	47,797 (38)	4,749 (43)	66,873 (72)	99,592(82)	36,659 (40)	34,212(48)
**Missing**	1,215	834	356	1,975	1,596	852	15,729	89,091	78	60

Values in parentheses are percentages unless otherwise stated.
*Baseline refers to the index date.
Abbreviations: N, number of participants; BMI, body mass index; WC, waist circumference; HRT, hormone replacement therapy; US, United States; UK, United Kingdom; EPIC, European Prospective Investigation into Cancer and Nutrition[1]; NIH-AARP, Diet and Health Study; PLCO, Prostate, Lung, Colorectal, Ovarian Cancer Screening Trial; WHI, Women's Health Initiative; ARIC, Atherosclerosis Risk in Communities study; NA, not applicable.
[1]Denmark, France, Germany, Italy, the Netherlands, Norway, Spain, Sweden and the United Kingdom

Table S7: Characteristics* of the analytic cohorts of participants with at least 1 BMI reading.

Characteristic	Men				Women				
	**ARIC N = 6,240**	**PLCO N = 72,016**	**NIH-AARP N = 327,022**	**EPIC N = 94,600**	**ARIC N = 7,701**	**PLCO N = 74,565**	**NIH-AARP N = 216,030**	**EPIC N = 225,704**	**WHI N = 160,142**
**Age *, years, mean (SD)**	57.0 (6.0)	63.0 (5.0)	62.0 (5.0)	58.0 (9.0)	57.0 (6.0)	63.0 (5.0)	61.0 (5.0)	58.0 (10.0)	63.0 (7.0)
**Height*, meters, mean (SD)**	1.8 (0.1)	1.8 (0.1)	1.8 (0.1)	1.8 (0.1)	1.6 (0.1)	1.6 (0.1)	1.6 (0.1)	1.6 (0.1)	1.6 (0.1)
**Missing**	0	0	0	0	0	0	0	0	659
**BMI *, kg/m^2**	27.7 (4.3)	27.5 (4.2)	27.3 (4.2)	26.7 (3.6)	28.2 (6.0)	27.1 (5.5)	26.9 (5.8)	25.3 (4.4)	28.0 (5.9)
**Ethnicity**									
**White**	4,941 (79)	63,669 (88)	303,064 (94)		5,535 (72)	66,169 (89)	193,957 (91)		132,185 (83)
**Black**	1,299 (21)	3,223 (5)	8,737 (3)		2,166 (28)	4,173 (5)	11,595 (5)		19,316 (12)
**Other**	NA	5,082 (7)	11,422 (4)		NA	4,197 (6)	7,215 (3)		8,235 (5)
**Missing**	NA	42	3,799		NA	26	3,263		406
**Smoking**									
**Ever**	4,598 (74)	45,714 (63)	219,703 (70)	60,276 (65)	3,808 (50)	33,055 (44)	114,551 (55)	92,524 (44)	77,403 (49)
**Never**	1,626 (26)	26,289 (37)	94,481 (29)	31,754 (34)	3,882 (50)	41,505 (56)	93,976 (44)	155,532 (52)	80,638 (50)
**Missing**	16	13	12,838	2,570	11	5	7,503	17,648	2,101
**Alcohol consumption, units of alcohol per week***	4 (9)	9 (26)	8 (21)	10 (11)	1 (4)	3 (9)	3 (8)	4 (6)	2 (5)
**Missing**	22	16,636	0	29,760	12	15,005	0	42,259	134
**HRT**									
**Ever**					4187 (54)	49,280 (67)	80,575 (62)	45,636 (24)	89,947 (56)
**Never**					2,272 (35)	24,823 (33)	50,310 (23)	145,960 (65)	70,056 (44)
**Missing**					1,242	462	85,145	34,108	139

* Mean (SD); n ()

[1] Values in parentheses are percentages unless otherwise stated.

Abbreviations: N, number of participants; BMI, body mass index; WC, waist circumference; HRT, hormone replacement therapy; US, United States; UK, United Kingdom; EPIC, European Prospective Investigation into Cancer and Nutrition[1]; NIH-AARP, Diet and Health Study; PLCO, Prostate, Lung, Colorectal, Ovarian Cancer Screening Trial; WHI, Women's Health Initiative; ARIC, Atherosclerosis Risk in Communities study.

[1]Denmark, France, Germany, Italy, the Netherlands, Norway, Spain, Sweden and the United Kingdom.

Findings from analysis using predicted BMI from participants with at least 3 measured BMI readings

a) Analysis of overweight-years exposure

Table S8: Hazard ratio of specific cancers per 100 overweight-years and per 5-unit baseline BMI, ABACus 2 Consortium.

Outcomes	Number of cancer events	Overweight-years (per 100 kg-years/m^2)				BMI (per 5 units)			
		Age-adjusted HR (95% CI)	I^2	MV-adjusted HR (95% CI)	I^2	Age-adjusted HR (95% CI)	I^2	MV-adjusted HR (95% CI)	I^2
**Men**									
†Total Cancers	85,341	1.02 (0.98,1.07)	0.69	1.02 (0.98,1.06)	0.74	1.01 (0.99,1.03)	0.35	1.01 (0.98,1.05)	0.61
OBR-cancers	12,959	1.20 (1.19,1.21)	0.00	1.20 (1.19,1.21)	0.00	1.20 (1.18,1.22)	0.00	1.20 (1.19,1.21)	0.00
NOR-cancers	64,743	0.97 (0.96,0.99)	0.05	0.97 (0.94,1.00)	0.54	0.97 (0.96,0.98)	0.00	0.96 (0.94,0.99)	0.17
NOR-cancers excluding lung and prostate	26,178	1.06 (1.03,1.08)	0.02	1.05 (1.02,1.09)	0.05	1.05 (1.03,1.07)	0.00	1.04 (1.01,1.08)	0.00
**Specific cancer sites**									
Colorectal	6,037	1.22 (1.00,1.49)	0.72	1.21 (1.00,1.48)	0.70	1.17 (1.12,1.23)	0.18	1.17 (1.12,1.23)	0.08
Pancreas	1,957	1.11 (1.02,1.21)	0.00	1.11 (1.02,1.2)	0.00	1.09 (0.97,1.21)	0.00	1.09 (0.98,1.21)	0.00
Kidney	1,967	1.27 (1.10,1.46)	0.23	1.26 (1.09,1.45)	0.24	1.31 (1.18,1.45)	0.22	1.30 (1.17,1.44)	0.22
Bladder	4,018	1.10 (1.03,1.18)	0.00	1.09 (1.01,1.18)	0.00	1.09 (1.03,1.16)	0.00	1.07 (1.00,1.15)	0.00
Lung	8,559	0.92 (0.85,0.99)	0.44	0.90 (0.83,0.98)	0.55	0.84 (0.72,0.98)	0.84	0.81 (0.71,0.93)	0.83
Prostate	30,006	0.95 (0.88,1.03)	0.69	0.95 (0.88,1.02)	0.66	0.97 (0.89,1.05)	0.82	0.97 (0.90,1.03)	0.73
**Women**									
†Total Cancers	63,732	1.05 (1.02,1.07)	0.69	1.06 (1.04,1.07)	0.37	1.04 (1.00,1.08)	0.85	1.05 (1.02,1.09)	0.83
OBR-cancers	36,509	1.09 (1.08,1.11)	0.08	1.10 (1.09,1.12)	0.00	1.10 (1.07,1.12)	0.74	1.10 (1.07,1.14)	0.82
NOR-cancers	24,499	0.97 (0.96,0.99)	0.00	0.99 (0.98,1.01)	0.00	0.93 (0.85,1.03)	0.81	0.95 (0.85,1.06)	0.84
NOR-cancers excluding lung	16,352	1.01 (0.98,1.04)	0.36	1.03 (1.01,1.04)	0.00	0.99 (0.94,1.04)	0.67	1.00 (0.95,1.05)	0.62
**Specific cancer sites**									
Colorectal	6,251	1.10 (1.08,1.11)	0.00	1.09 (1.07,1.10)	0.00	1.08 (1.04,1.12)	0.33	1.07 (1.04,1.11)	0.10
Pancreas	2,019	1.06 (0.90,1.26)	0.69	1.04 (0.93,1.17)	0.52	1.02 (0.96,1.08)	0.16	1.02 (0.98,1.06)	0.00
Kidney	1,270	1.25 (1.16,1.34)	0.00	1.23 (1.15,1.31)	0.00	1.28 (1.10,1.49)	0.88	1.27 (1.09,1.47)	0.87
Lung	8,114	0.86 (0.80,0.93)	0.77	0.90 (0.85,0.95)	0.49	0.86 (0.79,0.94)	0.75	0.88 (0.81,0.96)	0.73
Endometrial	3,931	1.36 (1.20,1.55)	0.95	1.37 (1.21,1.54)	0.92	1.33 (1.16,1.52)	0.94	1.36 (1.16,1.59)	0.95
Ovarian	2,717	1.02 (0.93,1.12)	0.55	1.03 (0.94,1.13)	0.48	0.99 (0.89,1.10)	0.70	1.01 (0.91,1.12)	0.69
Post-menopausal breast cancer	17,582	1.01 (0.98,1.04)	0.28	1.03 (0.99,1.08)	0.41	1.05 (1.00,1.10)	0.61	1.07 (1.01,1.13)	0.72

*Multivariable adjusted models: baseline age, ethnicity, alcohol, smoking, HRT.
† The sum of OBR and NOR cancer does not equal total cancers as non-melanoma skin cancers were excluded in the EPIC cohort analyses.
Abbreviations: OBR, obesity-related; NOR, non-obesity-related; CI, confidence interval; HR, hazard ratio; BMI, body mass index; MV, multivariable; SD, standard deviation.

Table S9: Comparison of the overweight degree and duration per 10 units and per 10 years respectively, ABACus 2 Consortium.

Outcomes	Number of cancer events	Degree of Overweight (per 10 units)				Duration of Overweight (per 10 years)			
		Age-adjusted HR (95% CI)	I^2	MV-adjusted HR (95% CI)	I^2	Age-adjusted HR (95% CI)	I^2	MV-adjusted HR (95% CI)	I^2
**Men**									
†Total Cancers	85,341	1.00 (0.99,1.01)	0.91	1.00 (0.99,1.01)	0.91	1.01 (0.99,1.02)	0.29	1.01 (1.00,1.02)	0.13
OBR-cancers	12,959	1.02 (1.01,1.02)	0.80	1.02 (1.01,1.02)	0.79	1.06 (1,10.11)	0.76	1.06 (1.00,1.11)	0.76
NOR-cancers	64,743	1.00 (1.00,1.00)	0.00	1.00 (1.00,1.00)	0.00	0.99 (0.99,1.00)	0.00	1.00 (0.98,1.02)	0.71
NOR-cancers excluding lung and prostate	26,178	1.00 (0.99,1.02)	0.86	1.00 (0.99,1.02)	0.86	1.03 (1.01,1.05)	0.07	1.03 (1.01,1.05)	0.16
**Specific cancer sites**									
Colorectal	6,037	1.01 (0.99,1.04)	0.86	1.01 (1.00,1.02)	0.47	1.04 (0.98,1.09)	0.72	1.04 (0.99,1.09)	0.61
Pancreas	1,957	1.01 (1.00,1.02)	0.08	1.01 (1.00,1.02)	0.00	1.05 (1.02,1.09)	0.00	1.05 (1.02,1.09)	0.00
Kidney	1,967	1.01 (0.99,1.03)	0.29	1.01 (0.99,1.03)	0.29	1.10 (0.94,1.28)	0.63	1.09 (0.90,1.33)	0.66
Bladder	4,018	1.01 (1.00,1.02)	0.00	1.01 (1.00,1.02)	0.00	1.03 (0.98,1.07)	0.00	1.02 (0.98,1.07)	0.00
Lung	8,559	1.00 (0.98,1.01)	0.62	1.00 (0.97,1.02)	0.91	0.95 (0.90,1.01)	0.75	0.95 (0.88,1.03)	0.86
Prostate	30,006	1.00 (0.99,1.01)	0.88	0.99 (0.98,1.00)	0.76	0.99 (0.97,1.01)	0.22	0.99 (0.96,1.02)	0.68
**Women**									
†Total Cancers	63,732	1.00 (1.00,1.01)	0.90	1.01 (1.00,1.01)	0.94	1.02 (1.00,1.04)	0.71	1.02 (1.00,1.04)	0.67
OBR-cancers	36,509	1.00 (1.00,1.01)	0.92	1.01 (1.00,1.01)	0.92	1.03 (1.01,1.06)	0.78	1.04 (1.01,1.07)	0.84
NOR-cancers	24,499	1.00 (0.99,1.01)	0.87	1.00 (0.99,1.01)	0.87	0.99 (0.93,1.05)	0.85	1.00 (0.95,1.05)	0.79
NOR-cancers excluding lung	16,352	1.00 (0.99,1.01)	0.80	1.00 (0.99,1.01)	0.83	1.03 (1.00,1.06)	0.46	1.03 (1.01,1.05)	0.21
**Specific cancer sites**									
Colorectal	6,251	1.01 (1.00,1.01)	0.00	1.01 (1.00,1.01)	0.00	1.05 (1.01,1.09)	0.45	1.05 (1.01,1.09)	0.35
Pancreas	2,019	1.00 (0.99,1.02)	0.46	1.01 (0.99,1.02)	0.46	1.06 (0.98,1.15)	0.53	1.07 (0.98,1.15)	0.49
Kidney	1,270	1.01 (0.99,1.03)	0.84	1.01 (0.99,1.03)	0.84	1.16 (1.00,1.35)	0.73	1.15 (0.99,1.33)	0.72
Lung	8,114	0.99 (0.98,1.00)	0.70	0.99 (0.98,1.01)	0.78	0.95 (0.89,1.02)	0.82	0.97 (0.9,1.04)	0.84
Endometrial	3,931	1.02 (1.01,1.04)	0.91	1.02 (1.00,1.04)	0.91	1.15 (1.00,1.32)	0.95	1.14 (0.99,1.31)	0.95
Ovarian	2,717	1.01 (1.00,1.01)	0.00	1.01 (1.00,1.02)	0.35	1.02 (0.98,1.05)	0.00	1.01 (0.98,1.05)	0.00
Post-menopausal breast cancer	17,582	1.00 (0.99, 1.00)	0.80	1.00 (0.99,1.01)	0.91	0.99 (0.95,1.03)	0.83	1.00 (0.96,1.04)	0.83

*Multivariable adjusted models: baseline age, ethnicity, alcohol, smoking, HRT.

† The sum of OBR and NOR cancer does not equal total cancers as non-melanoma skin cancers were excluded in the EPIC cohort analyses.

Degree of overweight is the cumulative sum of the number of BMI units ≥25 kg/m^2

Duration of overweight is the cumulative sum of the duration overweight (BMI ≥25 kg/m^2).

Abbreviations: OBR, obesity-related; NOR, non-obesity-related;CI, confidence interval; HR, hazard ratio; BMI, body mass index; MV, multivariable.

Table S10: Comparison of the overweight-years metric and BMI using Harrell's C-statistic.

Characteristic	C-statistic overweight - years with BMI (95% CI)	Difference in c-statistic between overweight-years with BMI combined compared with overweight-years (95% CI)	Difference in c-statistic between overweight-years with BMI combined and BMI (95% CI)
**Men**			
Total Cancers	0.599 (0.564,0.633)	-0.001 (-0.002,0.001)	-0.001 (-0.002,0.001)
OBR-cancers	0.612 (0.577,0.646)	-0.000 (-0.002,0.001)	0.001 (0.000,0.001)
NOR-cancers	0.595 (0.544,0.645)	0.001 (-0.001,0.002)	0.001 (-0.001,0.003)
NOR-cancers excluding lung and prostate	0.604 (0.522,0.680)	-0.000 (-0.001,0.000)	0.000 (-0.001,0.001)
**Specific cancer sites**			
Colorectal	0.641 (0.629,0.654)	-0.001 (-0.002,0.001)	0.000 (-0.000,0.001)
Pancreas	0.610 (0.563,0.656)	-0.000 (-0.002,0.002)	0.003 (-0.001,0.006)
Kidney	0.601 (0.580,0.622)	0.006 (0.001,0.011)	0.001 (-0.001,0.003)
Bladder	0.680 (0.613,0.740)	0.000 (-0.005,0.006)	0.000 (-0.005,0.006)
Lung	0.727 (0.693,0.758)	0.004 (0.002,0.005)	0.003 (0.002,0.005)
Prostate	0.607 (0.588,0.626)	-0.001 (-0.002,0.001)	-0.001 (-0.002,0.000)
**Women**			
Total Cancers	0.582 (0.559,0.604)	0.001 (-0.003,0.004)	0.001 (-0.004,0.005)
OBR-cancers	0.576 (0.549,0.603)	0.004 (-0.001,0.009)	0.001 (-0.003,0.005)
NOR-cancers	0.641 (0.576,0.701)	0.002 (-0.001,0.005)	0.001 (-0.002,0.003)
NOR-cancers excluding lung	0.598 (0.553,0.642)	0.001 (-0.001,0.004)	0.001 (-0.002,0.003)
**Specific cancer sites**			
Colorectal	0.627 (0.585,0.668)	0.000 (-0.000,0.001)	0.001 (-0.001,0.002)
Pancreas	0.638 (0.587,0.685)	0.001 (-0.001,0.004)	0.000 (-0.001,0.001)
Kidney	0.626 (0.600,0.650)	0.013 (0.000,0.026)	-0.001 (-0.003,0.001)
Lung	0.746 (0.701,0.785)	0.001 (-0.002,0.004)	0.000 (-0.001,0.002)
Endometrial	0.626 (0.579,0.671)	0.010 (0.001,0.018)	-0.000 (-0.004,0.004)
Ovarian	0.585 (0.543,0.626)	0.000 (-0.000,0.000)	0.001 (-0.002,0.004)
Post-menopausal breast cancer	0.606 (0.506,0.698)	0.003 (-0.001,0.006)	-0.000 (-0.003,0.002)

Key: Green – significant difference in C-statistic.
*All models were multivariable adjusted, including baseline age, ethnicity, alcohol, smoking, HRT.
Abbreviations: SE, standard error; OBR, obesity-related; NOR, non-obesity-related;BMI, body mass index; CI, confidence interval; MV, multivariable-adjusted.

a) Analysis of obese-years exposure

Table S11: Hazard ratio of specific cancers per standard deviation obese-years and BMI, ABACus 2 Consortium.

Outcomes	Number of cancer events	Obese-years (per SD)				BMI (per SD)			
		Age-adjusted HR (95% CI)	I^2	MV-adjusted HR (95% CI)	I^2	Age-adjusted HR (95% CI)	I^2	MV-adjusted HR (95% CI)	I^2
**Men**									
†Total Cancers	85,341	1.01 (0.98,1.05)	0.75	1.01 (0.98,1.05)	0.74	1.01 (0.99,1.03)	0.34	1.01 (0.98,1.04)	0.66
OBR-cancers	12,959	1.09 (1.07,1.11)	0.00	1.09 (1.07,1.11)	0.00	1.17 (1.16,1.18)	0.00	1.16 (1.15,1.18)	0.00
NOR-cancers	64,743	0.99 (0.97,1.01)	0.33	0.98 (0.95,1.01)	0.67	0.98 (0.97,0.99)	0.00	0.97 (0.96,0.99)	0.35
NOR-cancers excluding lung and prostate	26,178	1.03 (0.98,1.09)	0.61	1.03 (0.98,1.09)	0.60	1.04 (1.02,1.05)	0.00	1.03 (1,1.07)	0.25
**Specific cancer sites**									
Colorectal	6,037	1.09 (0.99,1.20)	0.69	1.09 (0.99,1.20)	0.69	1.14 (1.10,1.18)	0.15	1.14 (1.09,1.18)	0.21
Pancreas	1,957	1.04 (0.99,1.10)	0.00	1.04 (0.99,1.10)	0.00	1.07 (1.02,1.12)	0.00	1.08 (1.02,1.14)	0.00
Kidney	1,967	1.11 (1.07,1.16)	0.00	1.10 (1.06,1.15)	0.00	1.25 (1.15,1.36)	0.19	1.25 (1.15,1.35)	0.14
Bladder	4,018	1.05 (0.96,1.14)	0.56	1.05 (0.97,1.14)	0.47	1.08 (1.03,1.13)	0.00	1.06 (1.01,1.12)	0.00
Lung	8,559	0.97 (0.94,1.00)	0.00	0.97 (0.93,1.01)	0.00	0.87 (0.77,0.98)	0.83	0.85 (0.77,0.93)	0.82
Prostate	30,006	0.96 (0.93,0.98)	0.33	0.96 (0.93,0.98)	0.18	0.97 (0.91,1.04)	0.85	0.97 (0.92,1.03)	0.78
**Women**									
†Total Cancers	63,732	1.03 (1.00,1.05)	0.7	1.04 (1.02,1.05)	0.48	1.04 (1.00,1.09)	0.83	1.06 (1.01,1.11)	0.85
OBR-cancers	36,509	1.06 (1.02,1.09)	0.74	1.06 (1.03,1.10)	0.75	1.10 (1.07,1.14)	0.72	1.11 (1.07,1.15)	0.79
NOR-cancers	24,499	0.96 (0.91,1.00)	0.66	0.97 (0.93,1.02)	0.68	0.91 (0.83,1.01)	0.91	0.94 (0.84,1.04)	0.90
NOR-cancers excluding lung	16,352	1.00 (0.98,1.02)	0.00	1.02 (0.99,1.04)	0.00	0.99 (0.92,1.05)	0.69	1.01 (0.93,1.09)	0.65
**Specific cancer sites**									
Colorectal	6,251	1.06 (1.04,1.08)	0.00	1.06 (1.04,1.08)	0.00	1.09 (1.03,1.14)	0.47	1.07 (1.03,1.12)	0.28
Pancreas	2,019	1.02 (0.89,1.18)	0.65	1.01 (0.91,1.13)	0.51	1.02 (0.95,1.09)	0.13	1.02 (0.98,1.06)	0.00
Kidney	1,270	1.14 (1.08,1.21)	0.00	1.13 (1.07,1.19)	0.00	1.33 (1.13,1.56)	0.87	1.31 (1.11,1.54)	0.86
Lung	8,114	0.90 (0.85,0.96)	0.45	0.93 (0.88,0.97)	0.22	0.85 (0.79,0.91)	0.67	0.87 (0.81,0.93)	0.61
Endometrial	3,931	1.18 (1.11,1.26)	0.85	1.18 (1.10,1.27)	0.80	1.35 (1.18,1.55)	0.92	1.38 (1.17,1.64)	0.94
Ovarian	2,717	1.01 (0.96,1.06)	0.00	1.02 (0.97,1.06)	0.00	0.99 (0.88,1.11)	0.74	1.00 (0.90,1.11)	0.67
Post-menopausal breast cancer	17,582	1.01 (0.96,1.05)	0.60	1.02 (0.97,1.06)	0.61	1.05 (1.01,1.10)	0.58	1.07 (1.02,1.12)	0.58

*Multivariable adjusted models: baseline age, ethnicity, alcohol, smoking, HRT.
† The sum of OBR and NOR cancer does not equal total cancers as non-melanoma skin cancers were excluded in the EPIC cohort analyses.
Abbreviations: OBR, obesity-related; NOR, non-obesity-related;CI, confidence interval; HR, hazard ratio; BMI, body mass index; MV, multivariable; SD, standard deviation.

Table S12: Hazard ratio of specific cancers per 100 obese-years and per 5-unit BMI, ABACus 2 Consortium.

Outcomes	Number of cancer events	Obese-years (per 100 kg-years/m^2)				BMI (per 5 units)			
		Age-adjusted HR (95% CI)	I^2	MV-adjusted HR (95% CI)	I^2	Age-adjusted HR (95% CI)	I^2	MV-adjusted HR (95% CI)	I^2
**Men**									
†Total Cancers	85,341	1.07 (0.93,1.22)	0.75	1.06 (0.92,1.23)	0.78	1.01 (0.99,1.03)	0.35	1.01 (0.98,1.05)	0.61
OBR-cancers	12,959	1.34 (1.23,1.46)	0.34	1.35 (1.25,1.45)	0.20	1.20 (1.18,1.22)	0.00	1.20 (1.19,1.21)	0.00
NOR-cancers	64,743	0.95 (0.88,1.03)	0.39	0.95 (0.87,1.03)	0.52	0.97 (0.96,0.98)	0.00	0.96 (0.94,0.99)	0.17
NOR-cancers excluding lung and prostate	26,178	1.13 (0.86,1.48)	0.63	1.13 (0.86,1.49)	0.65	1.05 (1.03,1.07)	0.00	1.04 (1.01,1.08)	0.00
**Specific cancer sites**									
Colorectal	6,037	1.42 (0.96,2.08)	0.70	1.41 (0.97,2.06)	0.69	1.17 (1.12,1.23)	0.18	1.17 (1.12,1.23)	0.08
Pancreas	1,957	1.11 (0.89,1.39)	0.00	1.11 (0.89,1.39)	0.00	1.09 (0.97,1.21)	0.00	1.09 (0.98,1.21)	0.00
Kidney	1,967	1.43 (1.24,1.65)	0.00	1.41 (1.21,1.64)	0.00	1.31 (1.18,1.45)	0.22	1.30 (1.17,1.44)	0.22
Bladder	4,018	1.22 (0.80,1.86)	0.66	1.23 (0.79,1.90)	0.66	1.09 (1.03,1.16)	0.00	1.07 (1.00,1.15)	0.00
Lung	8,559	0.90 (0.81,1.00)	0.00	0.89 (0.78,1.02)	0.00	0.84 (0.72,0.98)	0.84	0.81 (0.71,0.93)	0.83
Prostate	30,006	0.84 (0.78,0.91)	0.03	0.84 (0.79,0.90)	0.00	0.97 (0.89,1.05)	0.82	0.97 (0.90,1.03)	0.73
**Women**									
†Total Cancers	63,732	1.08 (1.03,1.13)	0.75	1.10 (1.06,1.14)	0.55	1.04 (1.00,1.08)	0.85	1.05 (1.02,1.09)	0.83
OBR-cancers	36,509	1.15 (1.11,1.20)	0.42	1.16 (1.12,1.20)	0.25	1.10 (1.07,1.12)	0.74	1.10 (1.07,1.14)	0.82
NOR-cancers	24,499	0.96 (0.94,0.98)	0.00	1.00 (0.97,1.02)	0.00	0.93 (0.85,1.03)	0.81	0.95 (0.85,1.06)	0.84
NOR-cancers excluding lung	16,352	1.03 (0.97,1.11)	0.48	1.06 (1.01,1.11)	0.06	0.99 (0.94,1.04)	0.67	1.00 (0.95,1.05)	0.62
**Specific cancer sites**									
Colorectal	6,251	1.15 (1.08,1.22)	0.00	1.13 (1.07,1.19)	0.00	1.08 (1.04,1.12)	0.33	1.07 (1.04,1.11)	0.10
Pancreas	2,019	1.07 (0.79,1.45)	0.68	1.04 (0.81,1.35)	0.55	1.02 (0.96,1.08)	0.16	1.02 (0.98,1.06)	0.00
Kidney	1,270	1.32 (1.17,1.49)	0.00	1.29 (1.17,1.42)	0.00	1.28 (1.1,1.49)	0.88	1.27 (1.09,1.47)	0.87
Lung	8,114	0.78 (0.66,0.92)	0.71	0.82 (0.71,0.96)	0.59	0.86 (0.79,0.94)	0.75	0.88 (0.81,0.96)	0.73
Endometrial	3,931	1.55 (1.31,1.84)	0.93	1.56 (1.33,1.82)	0.89	1.33 (1.16,1.52)	0.94	1.36 (1.16,1.59)	0.95
Ovarian	2,717	0.99 (0.89,1.11)	0.00	1.02 (0.92,1.14)	0.00	0.99 (0.89,1.1)	0.70	1.01 (0.91,1.12)	0.69
Post-menopausal breast cancer	17,582	1.02 (0.93,1.13)	0.45	1.06 (0.95,1.18)	0.48	1.05 (1.00,1.10)	0.61	1.07 (1.01,1.13)	0.72

*Multivariable adjusted models: baseline age, ethnicity, alcohol, smoking, HRT.

† The sum of OBR and NOR cancer does not equal total cancers as non-melanoma skin cancers were excluded in the EPIC cohort analyses.

Abbreviations: OBR, obesity-related; NOR, non-obesity-related;CI, confidence interval; HR, hazard ratio; BMI, body mass index; MV, multivariable; SD, standard deviation.

Table S13: Comparison of the obesity degree and duration per unit standard deviation, ABACus 2 Consortium.

Outcomes	Number of cancer events	Degree of Obese (per SD)				Duration of Obese (per SD)			
		Age-adjusted HR (95% CI)	I^2	MV-adjusted HR (95% CI)	I^2	Age-adjusted HR (95% CI)	I^2	MV-adjusted HR (95% CI)	I^2
**Men**									
†Total Cancers	85,341	1.01 (0.98,1.05)	0.73	1.01 (0.98,1.05)	0.72	1.02 (0.98,1.05)	0.65	1.02 (0.98,1.05)	0.79
OBR-cancers	12,959	1.06 (0.99,1.15)	0.91	1.06 (1.00,1.14)	0.88	1.08 (1.01,1.16)	0.86	1.08 (1.01,1.16)	0.86
NOR-cancers	64,743	0.99 (0.96,1.02)	0.61	0.99 (0.95,1.03)	0.85	1.00 (0.96,1.04)	0.81	1.00 (0.96,1.04)	0.81
NOR-cancers excluding lung and prostate	26,178	1.02 (0.97,1.08)	0.55	1.02 (0.96,1.09)	0.58	1.02 (1.00,1.04)	0.00	1.02 (1.00,1.04)	0.00
**Specific cancer sites**									
Colorectal	6,037	1.06 (0.98,1.14)	0.68	1.05 (0.97,1.14)	0.66	1.08 (0.98,1.20)	0.74	1.08 (0.99,1.17)	0.65
Pancreas	1,957	1.02 (0.91,1.14)	0.40	1.02 (0.91,1.14)	0.40	1.06 (1.00,1.12)	0.00	1.06 (1.00,1.12)	0.00
Kidney	1,967	1.03 (0.92,1.16)	0.33	1.03 (0.92,1.16)	0.32	1.06 (0.92,1.23)	0.40	1.06 (0.93,1.20)	0.26
Bladder	4,018	1.02 (0.81,1.28)	0.83	1.03 (0.85,1.26)	0.77	1.04 (0.94,1.15)	0.00	1.04 (0.96,1.12)	0.00
Lung	8,559	1.00 (0.92,1.08)	0.57	1.01 (0.9,1.13)	0.81	1.01 (0.88,1.16)	0.81	1.01 (0.86,1.20)	0.91
Prostate	30,006	0.98 (0.94,1.01)	0.51	0.97 (0.94,1.00)	0.16	0.97 (0.94,1.01)	0.53	0.97 (0.94,1.01)	0.49
**Women**									
†Total Cancers	63,732	1.03 (1.02,1.04)	0.00	1.03 (1.02,1.04)	0.00	1.03 (1.02,1.04)	0.00	1.04 (1.03,1.05)	0.00
OBR-cancers	36,509	1.03 (1.02,1.05)	0.31	1.04 (1.02,1.07)	0.64	1.05 (1.02,1.07)	0.56	1.05 (1.02,1.08)	0.67
NOR-cancers	24,499	1.01 (0.96,1.06)	0.78	1.02 (0.97,1.06)	0.72	1.00 (0.94,1.05)	0.81	1.01 (0.96,1.06)	0.77
NOR-cancers excluding lung	16,352	1.03 (0.98,1.08)	0.55	1.04 (1.00,1.09)	0.46	1.02 (0.97,1.08)	0.67	1.03 (0.98,1.08)	0.57
**Specific cancer sites**									
Colorectal	6,251	1.05 (1.01,1.09)	0.23	1.04 (1.01,1.07)	0.00	1.05 (1.02,1.09)	0.00	1.05 (1.02,1.08)	0.00
Pancreas	2,019	0.99 (0.88,1.12)	0.50	1.00 (0.88,1.13)	0.52	1.00 (0.86,1.17)	0.60	1.01 (0.86,1.19)	0.62
Kidney	1,270	1.06 (0.95,1.19)	0.51	1.05 (0.94,1.18)	0.55	1.12 (0.95,1.31)	0.67	1.10 (0.94,1.29)	0.69
Lung	8,114	0.95 (0.88,1.03)	0.52	0.96 (0.90,1.03)	0.44	0.95 (0.88,1.03)	0.66	0.97 (0.89,1.05)	0.68
Endometrial	3,931	1.13 (1.06,1.21)	0.69	1.14 (1.07,1.21)	0.70	1.20 (1.07,1.35)	0.79	1.21 (1.09,1.34)	0.75
Ovarian	2,717	1.04 (1.01,1.07)	0.00	1.04 (1.01,1.08)	0.00	1.08 (1.03,1.12)	0.00	1.08 (1.04,1.13)	0.00
Post-menopausal breast cancer	17,582	0.99 (0.95,1.03)	0.68	1.00 (0.96,1.04)	0.69	1.00 (0.96,1.04)	0.60	1.01 (0.96,1.06)	0.76

*Multivariable adjusted models: baseline age, ethnicity, alcohol, smoking, HRT.
† The sum of OBR and NOR cancer does not equal total cancers as non-melanoma skin cancers were excluded in the EPIC cohort analyses.
Degree of overweight is the cumulative sum of the number of BMI units ≥ 30 kg/m^2
Duration of overweight is the cumulative sum of the duration overweight (BMI ≥ 30 kg/m^2).
Abbreviations: OBR, obesity-related; NOR, non-obesity-related; CI, confidence interval; HR, hazard ratio; BMI, body mass index; MV, multivariable.

Table S14: Comparison of the obesity degree and duration per 10 units and per 10 years respectively, ABACus 2 Consortium.

Outcomes	Number of cancer events	Degree of Obese (per 10 units)				Duration of Obese (per 10 years)			
		Age-adjusted HR (95% CI)	I^2	MV-adjusted HR (95% CI)	I^2	Age-adjusted HR (95% CI)	I^2	MV-adjusted HR (95% CI)	I^2
**Men**									
†Total Cancers	85,341	1.01 (0.99,1.03)	0.84	1.00 (1.00,1.01)	0.36	1.03 (0.98,1.08)	0.70	1.02 (0.97,1.08)	0.78
OBR-cancers	12,959	1.03 (0.99,1.06)	0.93	1.03 (0.99,1.06)	0.93	1.12 (1.02,1.23)	0.87	1.12 (1.03,1.23)	0.84
NOR-cancers	64,743	1.00 (0.99,1.01)	0.67	1.00 (0.99,1.01)	0.62	1.00 (0.94,1.06)	0.82	0.99 (0.94,1.05)	0.81
NOR-cancers excluding lung and prostate	26,178	1.01 (0.98,1.04)	0.60	1.01 (0.98,1.03)	0.59	1.02 (1.00,1.05)	0.00	1.03 (1.00,1.05)	0.00
**Specific cancer sites**									
Colorectal	6,037	1.02 (0.99,1.06)	0.77	1.02 (0.99,1.06)	0.77	1.11 (0.97,1.27)	0.71	1.11 (0.97,1.27)	0.71
Pancreas	1,957	1.00 (0.97,1.04)	0.37	1.00 (0.97,1.04)	0.36	1.08 (1.01,1.17)	0.00	1.08 (1.00,1.16)	0.00
Kidney	1,967	1.01 (0.98,1.04)	0.07	1.01 (0.98,1.04)	0.06	1.08 (0.91,1.29)	0.32	1.08 (0.92,1.26)	0.19
Bladder	4,018	1.01 (0.96,1.06)	0.79	1.01 (0.97,1.06)	0.72	1.04 (0.96,1.12)	0.00	1.05 (0.98,1.13)	0.00
Lung	8,559	1.00 (0.97,1.03)	0.68	1.00 (0.97,1.04)	0.80	1.01 (0.84,1.23)	0.82	1.02 (0.82,1.28)	0.90
Prostate	30,006	0.99 (0.97,1.01)	0.79	0.99 (0.98,1.00)	0.19	0.97 (0.92,1.02)	0.49	0.96 (0.91,1.01)	0.51
**Women**									
†Total Cancers	63,732	1.01 (1.00,1.01)	0.84	1.01 (1.01,1.01)	0.00	1.04 (1.01,1.07)	0.69	1.05 (1.02,1.07)	0.49
OBR-cancers	36,509	1.01 (1.00,1.02)	0.84	1.01 (1.01,1.02)	0.53	1.06 (1.01,1.11)	0.77	1.07 (1.01,1.13)	0.82
NOR-cancers	24,499	1.00 (0.99,1.01)	0.78	1.00 (1.00,1.01)	0.54	1.00 (0.92,1.07)	0.81	1.00 (0.93,1.08)	0.81
NOR-cancers excluding lung	16,352	1.00 (0.99,1.02)	0.85	1.01 (1.00,1.02)	0.67	1.03 (0.96,1.10)	0.73	1.03 (0.97,1.10)	0.61
**Specific cancer sites**									
Colorectal	6,251	1.01 (0.99,1.02)	0.65	1.01 (0.99,1.02)	0.64	1.06 (1.00,1.13)	0.35	1.06 (1.00,1.12)	0.27
Pancreas	2,019	1.00 (0.97,1.03)	0.51	1.00 (0.97,1.03)	0.52	1.01 (0.85,1.21)	0.61	1.02 (0.85,1.23)	0.62
Kidney	1,270	1.01 (0.99,1.03)	0.52	1.01 (0.99,1.03)	0.50	1.14 (0.91,1.42)	0.78	1.12 (0.90,1.39)	0.77
Lung	8,114	0.99 (0.97,1.01)	0.61	0.99 (0.98,1.01)	0.43	0.94 (0.83,1.06)	0.72	0.96 (0.85,1.07)	0.70
Endometrial	3,931	1.03 (1.00,1.05)	0.89	1.03 (1.00,1.05)	0.89	1.26 (1.02,1.56)	0.94	1.26 (1.02,1.56)	0.93
Ovarian	2,717	1.01 (1.00,1.02)	0.00	1.01 (1.00,1.02)	0.00	1.09 (1.02,1.17)	0.00	1.10 (1.03,1.18)	0.00
Post-menopausal breast cancer	17,582	1.00 (0.99,1.01)	0.60	1.00 (0.99,1.02)	0.80	1.00 (0.94,1.06)	0.66	1.02 (0.95,1.09)	0.73

*Multivariable adjusted models: baseline age, ethnicity, alcohol, smoking, HRT.
† The sum of OBR and NOR cancer does not equal total cancers as non-melanoma skin cancers were excluded in the EPIC cohort analyses.
Degree of overweight is the cumulative sum of the number of BMI units ≥30 kg/m^2
Duration of overweight is the cumulative sum of the duration overweight (BMI ≥ 30 kg/m^2).
Abbreviations: OBR, obesity-related; NOR, non-obesity-related; CI, confidence interval; HR, hazard ratio; BMI, body mass index; MV, multivariable.

Table S15: Comparison of Harrell's C-statistic of metrics, ABACus 2 Consortium.

Harrell's C-statistic(95% CI)									
**Characteristic**	**Obese-years**	**Baseline BMI**	**Difference in c-statistic between baseline BMI and obese-years**	**Obese - years with baseline BMI**	**Difference in c-statistic between obese-years with BMI combined compared with obese-years**	**Difference in c-statistic between obese-years with BMI combined and BMI**	**Degree of obesity**	**Duration of obesity**	**Difference in c-statistic between duration and degree of obesity**
**Men**									
Total Cancers	0.600 (0.566, 0.634)	0.589 (0.565, 0.613)	0.000 (-0.006, 0.007)	0.598 (0.562, 0.634)	-0.000 (-0.003, 0.002)	-0.001 (-0.006, 0.003)	0.598 (0.563, 0.633)	0.592 (0.564, 0.619)	-0.000 (-0.004, 0.003)
OR-cancers	0.608 (0.574, 0.640)	0.612 (0.578, 0.644)	0.001 (-0.003, 0.004)	0.620 (0.590, 0.648)	0.002 (-0.000, 0.005)	0.000 (-0.000, 0.001)	0.608 (0.575, 0.640)	0.611 (0.579, 0.642)	-0.001 (-0.004, 0.003)
NOR-cancers	0.592 (0.566, 0.618)	0.592 (0.570, 0.613)	0.000 (-0.001, 0.002)	0.597 (0.555, 0.637)	0.001 (-0.002, 0.003)	0.000 (-0.002, 0.002)	0.605 (0.580, 0.629)	0.591 (0.567, 0.614)	-0.001 (-0.003, 0.001)
NOR-cancers excluding lung and prostate	0.615 (0.546, 0.678)	0.615 (0.547, 0.678)	-0.000 (-0.003, 0.002)	0.619 (0.549, 0.684)	-0.000 (-0.001, 0.001)	0.000 (-0.002, 0.003)	0.615 (0.547, 0.678)	0.615 (0.547, 0.679)	0.000 (-0.001, 0.002)
**Specific cancer sites**									
Colorectal	0.624 (0.580, 0.665)	0.623 (0.589, 0.656)	0.001 (-0.002, 0.004)	0.637 (0.601, 0.672)	0.001 (-0.002, 0.005)	0.000 (-0.002, 0.002)	0.623 (0.577, 0.667)	0.626 (0.579, 0.671)	-0.000 (-0.003, 0.003)
Pancreas	0.604 (0.547, 0.658)	0.605 (0.549, 0.659)	-0.002 (-0.006, 0.002)	0.608 (0.562, 0.653)	0.001 (-0.001, 0.003)	0.003 (-0.002, 0.008)	0.604 (0.545, 0.659)	0.604 (0.545, 0.660)	0.000 (-0.003, 0.004)
Kidney	0.579 (0.550, 0.607)	0.595 (0.589, 0.601)	0.008 (-0.003, 0.018)	0.595 (0.584, 0.607)	0.008 (-0.003, 0.018)	0.001 (-0.004, 0.005)	0.580 (0.549, 0.610)	0.580 (0.557, 0.603)	0.006 (-0.005, 0.018)
Bladder	0.679 (0.613, 0.739)	0.678 (0.611, 0.739)	0.001 (-0.022, 0.025)	0.680 (0.613, 0.740)	0.003 (-0.007, 0.012)	0.002 (-0.015, 0.018)	0.679 (0.613, 0.739)	0.677 (0.611, 0.736)	-0.005 (-0.019, 0.009)
Lung	0.720 (0.694, 0.745)	0.726 (0.698, 0.753)	0.005 (0.000, 0.009)	0.727 (0.697, 0.755)	0.005 (0.001, 0.010)	0.001 (-0.001, 0.003)	0.720 (0.695, 0.744)	0.720 (0.695, 0.744)	0.001 (-0.002, 0.005)
Prostate	0.604 (0.590, 0.618)	0.603 (0.586, 0.619)	-0.001 (-0.004, 0.002)	0.609 (0.600, 0.619)	-0.000 (-0.003, 0.002)	0.000 (-0.001, 0.001)	0.604 (0.591, 0.618)	0.604 (0.590, 0.618)	-0.001 (-0.005, 0.003)
**Women**									
Total Cancers	0.570 (0.532, 0.607)	0.578 (0.556, 0.601)	0.000 (-0.000, 0.001)	0.579 (0.549, 0.607)	0.000 (-0.000, 0.001)	0.000 (-0.000, 0.000)	0.579 (0.558, 0.599)	0.579 (0.558, 0.598)	0.000 (-0.000, 0.001)
OR-cancers	0.560 (0.529, 0.590)	0.572 (0.545, 0.598)	0.003 (0.001, 0.005)	0.598 (0.535, 0.658)	0.003 (0.001, 0.005)	0.000 (-0.004, 0.005)	0.560 (0.530, 0.590)	0.568 (0.541, 0.595)	0.001 (0.000, 0.002)
NOR-cancers	0.639 (0.579, 0.695)	0.640 (0.577, 0.699)	0.002 (-0.001, 0.005)	0.653 (0.593, 0.709)	0.002 (-0.002, 0.006)	0.001 (-0.001, 0.002)	0.638 (0.577, 0.695)	0.639 (0.578, 0.695)	0.000 (-0.000, 0.001)
NOR-cancers excluding lung	0.587 (0.538, 0.635)	0.589 (0.540, 0.636)	0.001 (-0.002, 0.004)	0.590 (0.541, 0.637)	0.001 (-0.002, 0.004)	0.000 (-0.000, 0.001)	0.588 (0.539, 0.635)	0.588 (0.540, 0.634)	-0.001 (-0.002, 0.001)
**Specific cancer sites**									
Colorectal	0.625 (0.555, 0.689)	0.629 (0.572, 0.683)	-0.000 (-0.002, 0.001)	0.629 (0.569, 0.684)	-0.000 (-0.000, 0.000)	0.000 (-0.001, 0.001)	0.624 (0.555, 0.688)	0.624 (0.556, 0.688)	0.000 (-0.001, 0.002)
Pancreas	0.633 (0.582, 0.681)	0.632 (0.580, 0.681)	0.001 (-0.001, 0.004)	0.639 (0.589, 0.686)	0.001 (-0.000, 0.003)	-0.000 (-0.001, 0.001)	0.633 (0.582, 0.681)	0.633 (0.581, 0.683)	0.000 (-0.002, 0.003)
Kidney	0.619 (0.560, 0.674)	0.645 (0.595, 0.692)	0.011 (0.003, 0.019)	0.647 (0.594, 0.696)	0.011 (0.002, 0.019)	-0.001 (-0.003, 0.001)	0.619 (0.561, 0.674)	0.623 (0.568, 0.674)	0.007 (-0.001, 0.014)
Lung	0.744 (0.714, 0.772)	0.746 (0.713, 0.777)	0.002 (-0.002, 0.006)	0.741 (0.715, 0.766)	0.002 (-0.002, 0.006)	0.001 (-0.001, 0.002)	0.745 (0.715, 0.772)	0.744 (0.712, 0.773)	0.001 (-0.001, 0.002)
Endometrial	0.607 (0.570, 0.643)	0.625 (0.580, 0.668)	0.013 (0.002, 0.024)	0.618 (0.560, 0.673)	0.011 (0.003, 0.019)	0.000 (-0.002, 0.002)	0.607 (0.569, 0.643)	0.612 (0.575, 0.649)	-0.003 (-0.015, 0.010)
Ovarian	0.580 (0.536, 0.623)	0.582 (0.542, 0.622)	0.001 (-0.003, 0.004)	0.589 (0.546, 0.630)	0.000 (-0.003, 0.003)	0.001 (0.000, 0.001)	0.580 (0.536, 0.623)	0.581 (0.535, 0.625)	0.001 (-0.002, 0.004)
Post-menopausal breast cancer	0.575 (0.506, 0.642)	0.581 (0.511, 0.647)	0.003 (0.001, 0.005)	0.583 (0.511, 0.652)	0.003 (0.001, 0.006)	-0.000 (-0.000, 0.000)	0.576 (0.507, 0.641)	0.577 (0.508, 0.643)	0.000 (-0.001, 0.001)

Key: Green – significant difference in C-statistic.

*All models were multivariable adjusted, including baseline age, ethnicity, alcohol, smoking, HRT.

Abbreviations: SE, standard error; OR, obesity-related; NOR, non-obesity-related; BMI, body mass index.

a) **<u>Findings from analyses using measured and not predicted BMI from participants with at least 3 measured BMI readings</u>**

Analysis of overweight-years exposure

Table S16: Hazard ratio of cancers per standard deviation of overweight-years and BMI.

Outcomes	Number of cancer events	Overweight-years (per SD)				BMI (per SD)			
		Age-adjusted HR (95% CI)	I^2	MV-adjusted HR (95% CI)	I^2	Age-adjusted HR (95% CI)	I^2	MV-adjusted HR (95% CI)	I^2
**Men**									
†Total Cancers	85,341	1.01 (0.97,1.05)	0.87	1.01 (0.96,1.05)	0.91	1.01 (0.99,1.03)	0.34	1.01 (0.98,1.04)	0.67
OBR-cancers	12,959	1.10 (1.05,1.15)	0.72	1.09 (1.04,1.15)	0.77	1.16 (1.16,1.16)	0.00	1.16 (1.15,1.17)	0.00
NOR-cancers	64,743	0.98 (0.93,1.03)	0.84	0.98 (0.93,1.03)	0.84	0.96 (0.93,1.00)	0.79	0.96 (0.93,1.00)	0.71
NOR-cancers excluding lung and prostate	26,178	1.03 (0.93,1.13)	0.79	1.03 (0.94,1.12)	0.76	1.04 (1.01,1.07)	0.00	1.03 (0.99,1.07)	0.02
**Specific cancer sites**									
Colorectal	6,037	1.09 (1.02,1.17)	0.56	1.10 (1.02,1.17)	0.57	1.13 (1.09,1.18)	0.09	1.13 (1.09,1.18)	0.19
Pancreas	1,957	1.05 (0.94,1.18)	0.44	1.05 (0.94,1.17)	0.44	1.07 (1.02,1.12)	0.00	1.07 (1.02,1.12)	0.00
Kidney	1,967	1.13 (1.01,1.25)	0.53	1.12 (1.01,1.24)	0.50	1.25 (1.19,1.31)	0.00	1.24 (1.18,1.3)	0.00
Bladder	4,018	1.08 (1.06,1.11)	0.00	1.08 (1.05,1.11)	0.00	1.08 (1.02,1.15)	0.00	1.07 (1.01,1.14)	0.00
Lung	8,559	0.96 (0.87,1.06)	0.78	0.96 (0.86,1.06)	0.79	0.87 (0.78,0.97)	0.82	0.85 (0.77,0.93)	0.82
Prostate	30,006	0.97 (0.91,1.03)	0.83	0.96 (0.90,1.03)	0.84	0.97 (0.91,1.04)	0.85	0.97 (0.92,1.03)	0.78
**Women**									
†Total Cancers	63,732	1.03 (1.00,1.05)	0.58	1.03 (1.01,1.07)	0.65	1.05 (1.01,1.09)	0.84	1.06 (1.01,1.11)	0.86
OBR-cancers	36,509	1.06 (1.03,1.09)	0.50	1.06 (1.03,1.10)	0.67	1.11 (1.07,1.15)	0.74	1.12 (1.08,1.16)	0.81
NOR-cancers	24,499	0.96 (0.92,1.00)	0.61	0.98 (0.94,1.02)	0.68	0.91 (0.82,1.01)	0.91	0.93 (0.83,1.05)	0.91
NOR-cancers excluding lung	16,352	1.01 (0.97,1.04)	0.27	1.02 (0.96,1.08)	0.49	0.99 (0.92,1.06)	0.69	1.01 (0.93,1.09)	0.66
**Specific cancer sites**									
Colorectal	6,251	1.08 (1.05,1.11)	0.00	1.08 (1.05,1.11)	0.00	1.09 (1.04,1.14)	0.39	1.08 (1.03,1.12)	0.24
Pancreas	2,019	1.06 (0.93,1.21)	0.59	1.06 (0.94,1.21)	0.54	1.02 (0.95,1.09)	0.13	1.02 (0.97,1.07)	0.00
Kidney	1,270	1.18 (1.14,1.21)	0.00	1.16 (1.12,1.20)	0.00	1.33 (1.18,1.49)	0.83	1.31 (1.17,1.48)	0.83
Lung	8,114	0.90 (0.85,0.95)	0.33	0.92 (0.89,0.96)	0.00	0.85 (0.78,0.92)	0.72	0.87 (0.80,0.94)	0.67
Endometrial	3,931	1.21 (1.09,1.34)	0.81	1.21 (1.11,1.32)	0.65	1.37 (1.19,1.58)	0.93	1.40 (1.18,1.67)	0.95
Ovarian	2,717	1.01 (0.91,1.12)	0.46	1.01 (0.92,1.11)	0.43	0.99 (0.88,1.11)	0.73	1.01 (0.90,1.12)	0.67
Post-menopausal breast cancer	17,582	0.98 (0.96,0.99)	0.00	0.99 (0.97,1.01)	0.00	1.05 (1.01,1.10)	0.59	1.08 (1.02,1.14)	0.67

*Multivariable adjusted models: baseline age, ethnicity, alcohol, smoking, HRT.
† The sum of OBR and NOR cancer does not equal total cancers as non-melanoma skin cancers were excluded in the EPIC cohort analyses.
Abbreviations: OBR, obesity-related; NOR, non-obesity-related; CI, confidence interval; HR, hazard ratio; BMI, body mass index; MV, multivariable; SD, standard deviation.

Table S17: Hazard ratio of specific cancers per 100 overweight-years and per 5-unit baseline BMI, ABACus 2 Consortium.

Outcomes	Number of cancer events	Overweight-years (per 100 kg-years/m^2)				BMI (per 5 units)			
		Age-adjusted HR (95% CI)	I^2	MV-adjusted HR (95% CI)	I^2	Age-adjusted HR (95% CI)	I^2	MV-adjusted HR (95% CI)	I^2
**Men**									
†Total Cancers	85,341	1.02 (0.95,1.09)	0.90	1.01 (0.94,1.09)	0.92	1.01 (0.99,1.03)	0.35	1.01 (0.98,1.05)	0.61
OBR-cancers	12,959	1.16 (1.12,1.21)	0.04	1.16 (1.10,1.22)	0.37	1.20 (1.18,1.22)	0.00	1.20 (1.19,1.21)	0.00
NOR-cancers	64,743	0.96 (0.90,1.04)	0.68	0.97 (0.89,1.04)	0.74	0.96 (0.92,1.01)	0.80	0.95 (0.91,0.99)	0.61
NOR-cancers excluding lung and prostate	26,178	1.05 (0.89,1.23)	0.80	1.04 (0.89,1.22)	0.81	1.05 (1.02,1.09)	0.00	1.04 (1.00,1.08)	0.00
**Specific cancer sites**									
Colorectal	6,037	1.15 (1.09,1.21)	0.22	1.15 (1.09,1.21)	0.19	1.17 (1.12,1.23)	0.18	1.17 (1.12,1.23)	0.08
Pancreas	1,957	1.07 (0.89,1.28)	0.47	1.07 (0.90,1.28)	0.45	1.09 (1.02,1.16)	0.00	1.09 (1.02,1.16)	0.00
Kidney	1,967	1.20 (1.05,1.36)	0.34	1.19 (1.05,1.36)	0.29	1.31 (1.23,1.40)	0.00	1.30 (1.22,1.39)	0.00
Bladder	4,018	1.11 (1.01,1.21)	0.17	1.10 (1.01,1.21)	0.14	1.09 (1.03,1.16)	0.00	1.07 (1.00,1.15)	0.00
Lung	8,559	0.94 (0.82,1.08)	0.78	0.94 (0.81,1.09)	0.80	0.84 (0.72,0.98)	0.84	0.81 (0.71,0.93)	0.83
Prostate	30,006	0.95 (0.87,1.04)	0.72	0.95 (0.86,1.04)	0.71	0.97 (0.89,1.05)	0.82	0.97 (0.90,1.03)	0.74
**Women**									
†Total Cancers	63,732	1.03 (1.00,1.06)	0.63	1.04 (1.01,1.07)	0.55	1.04 (1.00,1.08)	0.85	1.05 (1.02,1.09)	0.83
OBR-cancers	36,509	1.06 (1.04,1.08)	0.12	1.07 (1.05,1.09)	0.15	1.10 (1.07,1.12)	0.74	1.10 (1.07,1.14)	0.82
NOR-cancers	24,499	0.95 (0.91,1.00)	0.72	0.97 (0.93,1.01)	0.68	0.92 (0.83,1.02)	0.91	0.94 (0.84,1.05)	0.90
NOR-cancers excluding lung	16,352	1.01 (0.98,1.04)	0.27	1.01 (0.96,1.07)	0.43	0.99 (0.94,1.05)	0.67	1.00 (0.94,1.07)	0.62
**Specific cancer sites**									
Colorectal	6,251	1.08 (1.02,1.15)	0.48	1.07 (1.02,1.12)	0.30	1.08 (1.04,1.12)	0.33	1.07 (1.04,1.11)	0.10
Pancreas	2,019	1.07 (0.94,1.22)	0.63	1.07 (0.95,1.21)	0.57	1.02 (0.96,1.08)	0.16	1.02 (0.98,1.06)	0.00
Kidney	1,270	1.19 (1.10,1.28)	0.26	1.17 (1.09,1.25)	0.05	1.29 (1.15,1.44)	0.85	1.28 (1.14,1.43)	0.84
Lung	8,114	0.90 (0.80,1.00)	0.76	0.92 (0.86,0.99)	0.49	0.86 (0.79,0.94)	0.75	0.88 (0.81,0.96)	0.73
Endometrial	3,931	1.24 (1.07,1.43)	0.95	1.24 (1.08,1.41)	0.92	1.33 (1.16,1.52)	0.94	1.36 (1.16,1.59)	0.95
Ovarian	2,717	1.01 (0.91,1.13)	0.50	1.02 (0.93,1.12)	0.40	0.99 (0.89,1.10)	0.70	1.01 (0.91,1.12)	0.69
Post-menopausal breast cancer	17,582	0.97 (0.94,1.00)	0.00	0.98 (0.95,1.01)	0.00	1.05 (1.00,1.10)	0.61	1.07 (1.01,1.13)	0.72

*Multivariable adjusted models: baseline age, ethnicity, alcohol, smoking, HRT.
† The sum of OBR and NOR cancer does not equal total cancers as non-melanoma skin cancers were excluded in the EPIC cohort analyses.
Abbreviations: OBR, obesity-related; NOR, non-obesity-related;CI, confidence interval; HR, hazard ratio; BMI, body mass index; MV, multivariable; SD, standard deviation.

Table S18: Hazard ratios of cancers per standard deviation overweight degree and duration.

Outcomes	Number of cancer events	Degree of Overweight (per SD)				Duration of Overweight (per SD)			
		Age-adjusted HR (95% CI)	I^2	MV-adjusted HR (95% CI)	I^2	Age-adjusted HR (95% CI)	I^2	MV-adjusted HR (95% CI)	I^2
**Men**									
†Total Cancers	85,341	1.01 (0.83,1.22)	0.98	1.01 (0.84,1.2)	0.98	1.01 (0.95,1.06)	0.78	1.00 (0.95,1.05)	0.00
OBR-cancers	12,959	1.08 (0.80,1.45)	0.94	1.08 (0.82,1.44)	0.94	1.01 (0.85,1.20)	0.85	0.98 (0.81,1.19)	0.00
NOR-cancers	64,743	0.97 (0.83,1.13)	0.97	0.97 (0.84,1.11)	0.96	1.01 (0.97,1.04)	0.59	1.00 (0.97,1.03)	0.00
NOR-cancers excluding lung and prostate	26,178	0.96 (0.80,1.16)	0.95	0.97 (0.80,1.16)	0.95	1.05 (0.97,1.13)	0.74	1.03 (0.95,1.12)	0.00
**Specific cancer sites**									
Colorectal	6,037	1.13 (0.77,1.66)	0.86	1.13 (0.79,1.63)	0.85	1.00 (0.88,1.14)	0.63	0.95 (0.79,1.15)	0.00
Pancreas	1,957	0.90 (0.47,1.72)	0.91	0.90 (0.47,1.73)	0.91	1.10 (0.91,1.32)	0.57	1.07 (0.87,1.31)	0.00
Kidney	1,967	1.01 (0.83,1.24)	0.6	1.01 (0.83,1.23)	0.57	1.08 (0.95,1.23)	0.32	0.97 (0.78,1.21)	0.00
Bladder	4,018	1.05 (0.93,1.18)	0.00	1.05 (0.92,1.20)	0.05	0.99 (0.96,1.03)	0.00	1.01 (0.93,1.10)	0.00
Lung	8,559	1.01 (0.53,1.93)	0.97	1.04 (0.58,1.87)	0.97	0.94 (0.78,1.14)	0.91	0.92 (0.76,1.12)	0.00
Prostate	30,006	0.93 (0.91,0.96)	0.00	0.93 (0.92,0.94)	0.00	0.99 (0.97,1.01)	0.00	1.00 (0.96,1.05)	0.00
**Women**									
†Total Cancers	63,732	1.00 (0.93,1.07)	0.91	1.00 (0.93,1.08)	0.92	1.02 (1.00,1.05)	0.66	1.03 (1.00,1.06)	0.72
OBR-cancers	36,509	1.00 (0.94,1.07)	0.86	1.01 (0.94,1.08)	0.87	1.02 (0.99,1.05)	0.39	1.02 (1.00,1.04)	0.00
NOR-cancers	24,499	1.01 (0.84,1.22)	0.92	1.02 (0.86,1.22)	0.91	1.04 (0.98,1.09)	0.75	1.05 (0.98,1.12)	0.85
NOR-cancers excluding lung	16,352	1.00 (0.90,1.12)	0.79	1.00 (0.90,1.12)	0.79	1.07 (1.00,1.14)	0.70	1.07 (1.00,1.15)	0.74
**Specific cancer sites**									
Colorectal	6,251	1.06 (0.97,1.15)	0.44	1.06 (0.99,1.14)	0.33	1.08 (0.99,1.18)	0.66	1.08 (1.00,1.18)	0.63
Pancreas	2,019	0.97 (0.66,1.43)	0.75	0.98 (0.67,1.44)	0.74	1.14 (0.99,1.31)	0.56	1.14 (0.98,1.32)	0.60
Kidney	1,270	1.03 (0.89,1.18)	0.50	1.02 (0.90,1.16)	0.33	1.16 (1.04,1.29)	0.09	1.15 (1.05,1.25)	0.00
Lung	8,114	0.96 (0.70,1.32)	0.92	0.97 (0.73,1.29)	0.90	0.99 (0.90,1.09)	0.76	1.02 (0.90,1.15)	0.86
Endometrial	3,931	1.12 (1.01,1.24)	0.87	1.11 (1.01,1.23)	0.86	1.05 (0.89,1.24)	0.93	1.05 (0.90,1.22)	0.91
Ovarian	2,717	1.03 (0.82,1.29)	0.69	1.03 (0.82,1.29)	0.70	0.99 (0.94,1.04)	0.00	0.99 (0.94,1.04)	0.00
Post-menopausal breast cancer	17,582	0.92 (0.90,0.94)	0.00	0.93 (0.90,0.95)	0.00	0.97 (0.93,1.01)	0.31	0.98 (0.94,1.02)	0.29

*Multivariable adjusted models: baseline age, ethnicity, alcohol, smoking, HRT.
† The sum of OBR and NOR cancer does not equal total cancers as non-melanoma skin cancers were excluded in the EPIC cohort analyses.
Degree of overweight is the cumulative sum of the number of BMI units ≥ 25 kg/m^2
Duration of overweight is the cumulative sum of the duration overweight (BMI ≥ 25 kg/m^2).
Abbreviations: OBR, obesity-related; NOR, non-obesity-related;CI, confidence interval; HR, hazard ratio; BMI, body mass index; MV, multivariable.

Table S19: Comparison of the overweight degree and duration per 10 units and per 10 years respectively, ABACus 2 Consortium.

Outcomes	Number of cancer events	Degree of Overweight (per 10 units)				Duration of Overweight (per 10 years)			
		Age-adjusted HR (95% CI)	I^2	MV-adjusted HR (95% CI)	I^2	Age-adjusted HR (95% CI)	I^2	MV-adjusted HR (95% CI)	I^2
**Men**									
†Total Cancers	85,341	1.04 (0.84,1.29)	0.98	1.04 (0.85,1.27)	0.97	1.00 (0.98,1.03)	0.77	1.00 (0.98,1.03)	0.77
OBR-cancers	12,959	1.14 (0.80,1.62)	0.95	1.14 (0.81,1.62)	0.95	1.01 (0.90,1.12)	0.85	1.01 (0.91,1.12)	0.84
NOR-cancers	64,743	0.98 (0.85,1.12)	0.95	0.98 (0.86,1.11)	0.95	1.01 (0.99,1.02)	0.66	1.01 (0.99,1.02)	0.66
NOR-cancers excluding lung and prostate	26,178	0.97 (0.81,1.17)	0.95	0.97 (0.81,1.17)	0.95	1.03 (0.98,1.08)	0.75	1.03 (0.98,1.07)	0.75
**Specific cancer sites**									
Colorectal	6,037	1.20 (0.74,1.97)	0.88	1.21 (0.75,1.93)	0.87	1.00 (0.92,1.09)	0.67	1.00 (0.92,1.09)	0.67
Pancreas	1,957	0.96 (0.49,1.90)	0.91	0.95 (0.48,1.90)	0.91	1.05 (0.95,1.17)	0.58	1.05 (0.95,1.17)	0.58
Kidney	1,967	1.03 (0.82,1.28)	0.69	1.02 (0.82,1.27)	0.67	1.04 (0.97,1.13)	0.35	1.05 (0.99,1.11)	0.23
Bladder	4,018	1.04 (0.90,1.20)	0.12	1.06 (0.90,1.25)	0.35	1.00 (0.98,1.02)	0.00	1.00 (0.98,1.02)	0.00
Lung	8,559	1.12 (0.56,2.22)	0.97	1.14 (0.60,2.15)	0.97	0.97 (0.86,1.08)	0.91	0.97 (0.85,1.10)	0.92
Prostate	30,006	0.93 (0.91,0.94)	0.00	0.93 (0.90,0.95)	0.00	1.00 (0.99,1.01)	0.00	0.99 (0.97,1.02)	0.48
**Women**									
†Total Cancers	63,732	1.01 (0.94,1.07)	0.92	1.01 (0.94,1.08)	0.93	1.01 (1.00,1.03)	0.65	1.02 (1.00,1.04)	0.78
OBR-cancers	36,509	1.01 (0.95,1.06)	0.87	1.01 (0.95,1.07)	0.88	1.01 (1.00,1.03)	0.38	1.01 (1.00,1.02)	0.04
NOR-cancers	24,499	1.04 (0.82,1.33)	0.93	1.04 (0.83,1.30)	0.91	1.02 (0.99,1.06)	0.77	1.03 (0.99,1.07)	0.85
NOR-cancers excluding lung	16,352	1.00 (0.91,1.11)	0.81	1.01 (0.91,1.11)	0.81	1.04 (1.01,1.08)	0.65	1.04 (1.00,1.09)	0.71
Colorectal	6,251	1.05 (0.94,1.16)	0.60	1.04 (0.95,1.15)	0.54	1.05 (1.00,1.11)	0.68	1.05 (1.00,1.10)	0.64
Pancreas	2,019	0.98 (0.71,1.37)	0.74	0.99 (0.71,1.39)	0.75	1.08 (0.99,1.17)	0.59	1.08 (0.98,1.18)	0.63
Kidney	1,270	1.03 (0.90,1.18)	0.54	1.02 (0.90,1.16)	0.43	1.09 (1.01,1.17)	0.25	1.09 (1.02,1.15)	0.01
Lung	8,114	1.01 (0.7,1.46)	0.91	1.02 (0.73,1.43)	0.89	0.99 (0.94,1.06)	0.77	1.01 (0.94,1.09)	0.86
Endometrial	3,931	1.12 (0.98,1.28)	0.92	1.11 (0.98,1.26)	0.91	1.03 (0.93,1.14)	0.93	1.03 (0.94,1.13)	0.92
Ovarian	2,717	1.04 (0.83,1.31)	0.71	1.05 (0.83,1.32)	0.72	0.99 (0.96,1.02)	0.00	0.99 (0.96,1.03)	0.00
Post-menopausal breast cancer	17,582	0.92 (0.90,0.95)	0.00	0.93 (0.90,0.96)	0.00	0.98 (0.96,1.00)	0.25	0.99 (0.96,1.01)	0.43

*Multivariable adjusted models: baseline age, ethnicity, alcohol, smoking, HRT.
† The sum of OBR and NOR cancer does not equal total cancers as non-melanoma skin cancers were excluded in the EPIC cohort analyses.
Degree of overweight is the cumulative sum of the number of BMI units ≥ 25 kg/m^2
Duration of overweight is the cumulative sum of the duration overweight (BMI ≥ 25 kg/m^2).
Abbreviations: OBR, obesity-related; NOR, non-obesity-related;CI, confidence interval; HR, hazard ratio; BMI, body mass index; MV, multivariable.

Table S20: Comparison of Harrell's C-statistic of metrics, ABACus 2 Consortium.

Harrell's C-statistic(95% CI)									
**Characteristic**	**Overweight-years**	**BMI**	**Difference in c-statistic between BMI and overweight-years**	**Overweight-years with BMI**	**Difference in c-statistic between overweight-years with BMI combined compared with overweight-years**	**Difference in c-statistic between overweight-years with BMI combined and BMI**	**Degree of overweight**	**Duration of overweight**	**Difference in c-statistic between duration and degree of overweight**
**Men**									
**Total Cancers**	0.601 (0.571, 0.629)	0.600 (0.571, 0.629)	-0.000 (-0.001, 0.000)	0.606 (0.574, 0.637)	0.000 (-0.000, 0.000)	0.000 (-0.000, 0.001)	0.601 (0.570, 0.630)	0.600 (0.570, 0.630)	-0.000 (-0.001, 0.000)
**OR-cancers**	0.607 (0.573, 0.640)	0.612 (0.579, 0.643)	0.005 (0.001, 0.008)	0.613 (0.579, 0.645)	0.005 (0.001, 0.009)	0.001 (-0.000, 0.002)	0.610 (0.574, 0.646)	0.608 (0.574, 0.641)	0.000 (-0.007, 0.008)
**NOR-cancers**	0.601 (0.569, 0.632)	0.601 (0.568, 0.634)	0.001 (-0.000, 0.001)	0.602 (0.567, 0.635)	0.000 (-0.000, 0.001)	0.000 (-0.000, 0.001)	0.601 (0.568, 0.633)	0.601 (0.567, 0.634)	0.000 (-0.000, 0.001)
**NOR-cancers excluding lung and prostate**	0.611 (0.539, 0.678)	0.610 (0.537, 0.677)	-0.001 (-0.004, 0.001)	0.615 (0.548, 0.678)	0.000 (-0.002, 0.002)	0.001 (-0.001, 0.003)	0.611 (0.541, 0.677)	0.611 (0.540, 0.677)	0.001 (-0.001, 0.004)
**Specific cancer sites**									
**Colorectal**	0.624 (0.584, 0.662)	0.624 (0.586, 0.662)	0.002 (-0.002, 0.006)	0.630 (0.593, 0.666)	0.003 (0.000, 0.006)	0.001 (-0.001, 0.002)	0.628 (0.584, 0.670)	0.622 (0.586, 0.656)	-0.002 (-0.004, 0.001)
**Pancreas**	0.609 (0.569, 0.647)	0.610 (0.566, 0.651)	0.001 (-0.002, 0.003)	0.611 (0.564, 0.657)	0.002 (-0.000, 0.005)	0.005 (-0.003, 0.013)	0.614 (0.566, 0.660)	0.612 (0.577, 0.646)	0.003 (-0.004, 0.011)
**Kidney**	0.591 (0.563, 0.618)	0.603 (0.584, 0.622)	0.007 (-0.004, 0.019)	0.603 (0.584, 0.622)	0.006 (-0.002, 0.015)	0.001 (-0.002, 0.005)	0.598 (0.561, 0.634)	0.599 (0.580, 0.619)	0.003 (-0.007, 0.014)
**Bladder**	0.702 (0.638, 0.759)	0.700 (0.634, 0.759)	-0.002 (-0.027, 0.023)	0.706 (0.640, 0.764)	0.004 (-0.013, 0.021)	0.006 (-0.012, 0.023)	0.703 (0.638, 0.762)	0.694 (0.628, 0.753)	-0.009 (-0.030, 0.011)
**Lung**	0.722 (0.694, 0.748)	0.726 (0.697, 0.754)	0.003 (-0.001, 0.008)	0.727 (0.698, 0.754)	0.004 (0.001, 0.008)	0.001 (-0.000, 0.002)	0.727 (0.691, 0.761)	0.727 (0.701, 0.751)	-0.000 (-0.012, 0.012)
**Prostate**	0.599 (0.571, 0.627)	0.599 (0.571, 0.626)	-0.000 (-0.000, 0.000)	0.600 (0.573, 0.626)	-0.000 (-0.000, 0.000)	-0.000 (-0.000, 0.000)	0.600 (0.572, 0.627)	0.599 (0.571, 0.627)	-0.000 (-0.001, 0.001)
**Women**									
**Total Cancers**	0.584 (0.551, 0.616)	0.585 (0.554, 0.617)	0.001 (-0.000, 0.001)	0.588 (0.556, 0.619)	0.001 (-0.000, 0.001)	0.000 (-0.000, 0.000)	0.585 (0.552, 0.617)	0.586 (0.554, 0.617)	-0.000 (-0.001, 0.001)
**OR-cancers**	0.560 (0.528, 0.592)	0.569 (0.542, 0.596)	0.007 (-0.000, 0.014)	0.577 (0.554, 0.599)	0.007 (0.001, 0.013)	0.000 (-0.000, 0.000)	0.567 (0.537, 0.596)	0.567 (0.538, 0.595)	-0.000 (-0.002, 0.001)
**NOR-cancers**	0.641 (0.581, 0.696)	0.641 (0.580, 0.699)	0.001 (-0.002, 0.004)	0.642 (0.579, 0.701)	0.002 (0.000, 0.004)	0.000 (-0.000, 0.001)	0.641 (0.580, 0.697)	0.641 (0.581, 0.697)	0.001 (0.000, 0.002)
**NOR-cancers excluding lung**	0.589 (0.543, 0.634)	0.590 (0.543, 0.635)	0.000 (-0.002, 0.003)	0.599 (0.555, 0.641)	0.002 (-0.001, 0.004)	0.000 (-0.000, 0.000)	0.589 (0.542, 0.635)	0.592 (0.545, 0.636)	0.002 (-0.003, 0.006)
**Specific cancer sites**									
**Colorectal**	0.623 (0.570, 0.672)	0.630 (0.593, 0.665)	-0.000 (-0.002, 0.002)	0.627 (0.587, 0.666)	-0.000 (-0.001, 0.001)	-0.000 (-0.000, 0.000)	0.628 (0.588, 0.667)	0.630 (0.592, 0.667)	-0.000 (-0.001, 0.001)
**Pancreas**	0.634 (0.583, 0.683)	0.634 (0.582, 0.683)	0.001 (-0.001, 0.003)	0.638 (0.584, 0.688)	0.000 (-0.001, 0.002)	-0.000 (-0.002, 0.002)	0.637 (0.586, 0.685)	0.633 (0.584, 0.679)	-0.001 (-0.005, 0.004)
**Kidney**	0.603 (0.583, 0.623)	0.626 (0.606, 0.646)	0.019 (0.005, 0.032)	0.628 (0.606, 0.650)	0.018 (0.004, 0.032)	-0.001 (-0.003, 0.001)	0.608 (0.584, 0.632)	0.621 (0.606, 0.636)	0.011 (-0.001, 0.024)
**Lung**	0.745 (0.713, 0.774)	0.747 (0.715, 0.776)	0.002 (-0.001, 0.006)	0.750 (0.725, 0.773)	0.002 (-0.001, 0.005)	0.000 (-0.000, 0.001)	0.746 (0.712, 0.778)	0.745 (0.716, 0.772)	-0.000 (-0.003, 0.003)
**Endometrial**	0.600 (0.564, 0.634)	0.627 (0.582, 0.669)	0.027 (0.006, 0.047)	0.628 (0.582, 0.671)	0.024 (0.007, 0.041)	-0.000 (-0.000, 0.000)	0.624 (0.569, 0.676)	0.614 (0.563, 0.662)	-0.008 (-0.016, 0.000)
**Ovarian**	0.586 (0.544, 0.626)	0.585 (0.545, 0.623)	0.000 (-0.004, 0.005)	0.587 (0.546, 0.627)	0.001 (-0.005, 0.006)	-0.001 (-0.003, 0.002)	0.586 (0.543, 0.627)	0.588 (0.549, 0.626)	0.001 (-0.005, 0.008)
**Post-menopausal breast cancer**	0.623 (0.570, 0.672)	0.630 (0.593, 0.665)	0.004 (0.002, 0.007)	0.627 (0.587, 0.666)	0.004 (0.002, 0.007)	0.000 (-0.001, 0.001)	0.628 (0.588, 0.667)	0.630 (0.592, 0.667)	-0.000 (-0.001, 0.001)

Key: Green – significant difference in C-statistic.

*All models were multivariable adjusted, including baseline age, ethnicity, alcohol, smoking, HRT.

Abbreviations: SE, standard error; OR, obesity-related;; NOR, non-obesity-related; BMI, body mass index.

a) Analysis of obese-years exposure

Table S21: Hazard ratio of cancers per standard deviation of obese-years and BMI, ABACus 2 Consortium.

Outcomes	Number of cancer events	Obese-years (per SD)				BMI (per SD)			
		Age-adjusted HR (95% CI)	I^2	MV-adjusted HR (95% CI)	I^2	Age-adjusted HR (95% CI)	I^2	MV-adjusted HR (95% CI)	I^2
**Men**									
†Total Cancers	85,341	1.00 (0.98,1.03)	0.8	1.00 (0.98,1.03)	0.79	1.01 (0.99,1.03)	0.34	1.01 (0.98,1.04)	0.67
OBR-cancers	12,959	1.06 (1.03,1.09)	0.64	1.06 (1.03,1.09)	0.64	1.16 (1.16,1.16)	0.00	1.16 (1.15,1.17)	0.00
NOR-cancers	64,743	0.98 (0.94,1.03)	0.85	0.98 (0.93,1.03)	0.88	0.96 (0.93,1.00)	0.79	0.96 (0.93,1.00)	0.71
NOR-cancers excluding lung and prostate	26,178	1.02 (0.97,1.07)	0.68	1.01 (0.97,1.06)	0.54	1.04 (1.01,1.07)	0.00	1.03 (0.99,1.07)	0.02
**Specific cancer sites**									
Colorectal	6,037	1.06 (1.03,1.10)	0.36	1.07 (1.03,1.11)	0.44	1.13 (1.09,1.18)	0.09	1.13 (1.09,1.18)	0.19
Pancreas	1,957	1.04 (1.00,1.07)	0.00	1.04 (1.00,1.08)	0.00	1.07 (1.02,1.12)	0.00	1.07 (1.02,1.12)	0.00
Kidney	1,967	1.07 (1.02,1.13)	0.08	1.07 (1.03,1.12)	0.00	1.25 (1.19,1.31)	0.00	1.24 (1.18,1.3)	0.00
Bladder	4,018	1.05 (1.00,1.09)	0.00	1.05 (1.00,1.10)	0.07	1.08 (1.02,1.15)	0.00	1.07 (1.01,1.14)	0.00
Lung	8,559	0.98 (0.9,1.06)	0.69	0.98 (0.90,1.07)	0.71	0.87 (0.78,0.97)	0.82	0.85 (0.77,0.93)	0.82
Prostate	30,006	0.96 (0.92,1.00)	0.48	0.96 (0.92,0.99)	0.43	0.97 (0.91,1.04)	0.85	0.97 (0.92,1.03)	0.78
**Women**									
†Total Cancers	63,732	1.02 (1.00,1.04)	0.58	1.03 (1.01,1.04)	0.24	1.05 (1.01,1.09)	0.84	1.06 (1.01,1.11)	0.86
OBR-cancers	36,509	1.04 (1.02,1.07)	0.6	1.05 (1.00,1.09)	0.69	1.11 (1.07,1.15)	0.74	1.12 (1.08,1.16)	0.81
NOR-cancers	24,499	0.97 (0.94,0.99)	0.00	0.98 (0.96,1.01)	0.31	0.91 (0.82,1.01)	0.91	0.93 (0.83,1.05)	0.91
NOR-cancers excluding lung	16,352	1.00 (0.99,1.02)	0.00	1.02 (1.00,1.04)	0.00	0.99 (0.92,1.06)	0.69	1.01 (0.93,1.09)	0.66
**Specific cancer sites**									
Colorectal	6,251	1.06 (1.03,1.10)	0.00	1.06 (1.03,1.09)	0.00	1.09 (1.04,1.14)	0.39	1.08 (1.03,1.12)	0.24
Pancreas	2,019	1.03 (0.88,1.20)	0.64	1.03 (0.88,1.19)	0.61	1.02 (0.95,1.09)	0.13	1.02 (0.97,1.07)	0.00
Kidney	1,270	1.12 (1.11,1.13)	0.00	1.11 (1.10,1.12)	0.00	1.33 (1.18,1.49)	0.83	1.31 (1.17,1.48)	0.83
Lung	8,114	0.91 (0.89,0.93)	0.00	0.93 (0.91,0.95)	0.00	0.85 (0.78,0.92)	0.72	0.87 (0.80,0.94)	0.67
Endometrial	3,931	1.18 (1.01,1.38)	0.8	1.18 (1.00,1.39)	0.75	1.37 (1.19,1.58)	0.93	1.40 (1.18,1.67)	0.95
Ovarian	2,717	1.00 (0.91,1.10)	0.53	1.01 (0.93,1.10)	0.5	0.99 (0.88,1.11)	0.73	1.01 (0.90,1.12)	0.67
Post-menopausal breast cancer	17,582	0.97 (0.95,1.00)	0.11	0.98 (0.96,1.01)	0.16	1.05 (1.01,1.10)	0.59	1.08 (1.02,1.14)	0.67

*Multivariable adjusted models: baseline age, ethnicity, alcohol, smoking, HRT.

† The sum of OBR and NOR cancer does not equal total cancers as non-melanoma skin cancers were excluded in the EPIC cohort analyses.

Abbreviations: OBR, obesity-related; NOR, non-obesity-related;CI, confidence interval; HR, hazard ratio; BMI, body mass index; MV, multivariable; SD, standard deviation.

Table S22: Hazard ratio of cancers per standard deviation 100 obese-years and 5-unit BMI, ABACus 2 Consortium.

Outcomes	Number of cancer events	Obese-years (per 100 kg-years/m^2)				BMI (per 5 units)			
		Age-adjusted HR (95% CI)	I^2	MV-adjusted HR (95% CI)	I^2	Age-adjusted HR (95% CI)	I^2	MV-adjusted HR (95% CI)	I^2
**Men**									
†Total Cancers	85,341	1.03 (0.90,1.18)	0.85	1.03 (0.90,1.18)	0.86	1.01 (0.99,1.03)	0.35	1.01 (0.98,1.05)	0.61
OBR-cancers	12,959	1.28 (1.23,1.32)	0.00	1.28 (1.22,1.34)	0.00	1.20 (1.18,1.22)	0.00	1.20 (1.19,1.21)	0.00
NOR-cancers	64,743	0.94 (0.79,1.12)	0.78	0.94 (0.78,1.12)	0.78	0.96 (0.92,1.01)	0.80	0.95 (0.91,0.99)	0.61
NOR-cancers excluding lung and prostate	26,178	1.07 (0.86,1.34)	0.71	1.07 (0.86,1.33)	0.71	1.05 (1.02,1.09)	0.00	1.04 (1.00,1.08)	0.00
**Specific cancer sites**									
Colorectal	6,037	1.27 (1.20,1.34)	0.00	1.27 (1.21,1.33)	0.00	1.17 (1.12,1.23)	0.18	1.17 (1.12,1.23)	0.08
Pancreas	1,957	1.13 (0.96,1.34)	0.00	1.13 (0.96,1.33)	0.00	1.09 (1.02,1.16)	0.00	1.09 (1.02,1.16)	0.00
Kidney	1,967	1.31 (1.14,1.52)	0.00	1.30 (1.14,1.48)	0.00	1.31 (1.23,1.4)	0.00	1.30 (1.22,1.39)	0.00
Bladder	4,018	1.17 (0.90,1.50)	0.52	1.16 (0.90,1.51)	0.51	1.09 (1.03,1.16)	0.00	1.07 (1.00,1.15)	0.00
Lung	8,559	0.94 (0.70,1.26)	0.64	0.94 (0.69,1.27)	0.68	0.84 (0.72,0.98)	0.84	0.81 (0.71,0.93)	0.83
Prostate	30,006	0.85 (0.79,0.92)	0.14	0.84 (0.78,0.91)	0.07	0.97 (0.89,1.05)	0.82	0.97 (0.9,1.03)	0.74
**Women**									
†Total Cancers	63,732	1.04 (1.00,1.09)	0.61	1.05 (1.02,1.09)	0.48	1.04 (1.00,1.08)	0.85	1.05 (1.02,1.09)	0.83
OBR-cancers	36,509	1.09 (1.03,1.14)	0.55	1.09 (1.05,1.14)	0.41	1.10 (1.07,1.12)	0.74	1.10 (1.07,1.14)	0.82
NOR-cancers	24,499	0.94 (0.89,1.00)	0.35	0.97 (0.92,1.03)	0.31	0.92 (0.83,1.02)	0.91	0.94 (0.84,1.05)	0.90
NOR-cancers excluding lung	16,352	1.00 (0.97,1.03)	0.00	1.04 (1.00,1.07)	0.00	0.99 (0.94,1.05)	0.67	1.00 (0.94,1.07)	0.62
**Specific cancer sites**									
Colorectal	6,251	1.10 (0.99,1.23)	0.61	1.10 (1.00,1.21)	0.50	1.08 (1.04,1.12)	0.33	1.07 (1.04,1.11)	0.10
Pancreas	2,019	1.05 (0.85,1.31)	0.62	1.05 (0.85,1.30)	0.59	1.02 (0.96,1.08)	0.16	1.02 (0.98,1.06)	0.00
Kidney	1,270	1.23 (1.11,1.35)	0.02	1.20 (1.10,1.31)	0.00	1.29 (1.15,1.44)	0.85	1.28 (1.14,1.43)	0.84
Lung	8,114	0.85 (0.75,0.96)	0.44	0.89 (0.82,0.98)	0.08	0.86 (0.79,0.94)	0.75	0.88 (0.81,0.96)	0.73
Endometrial	3,931	1.32 (1.09,1.61)	0.93	1.33 (1.11,1.58)	0.90	1.33 (1.16,1.52)	0.94	1.36 (1.16,1.59)	0.95
Ovarian	2,717	1.05 (0.88,1.26)	0.54	1.06 (0.90,1.25)	0.46	0.99 (0.89,1.10)	0.70	1.01 (0.91,1.12)	0.69
Post-menopausal breast cancer	17,582	0.96 (0.89,1.03)	0.30	0.97 (0.90,1.05)	0.33	1.05 (1.00,1.10)	0.61	1.07 (1.01,1.13)	0.72

*Multivariable adjusted models: baseline age, ethnicity, alcohol, smoking, HRT.
† The sum of OBR and NOR cancer does not equal total cancers as non-melanoma skin cancers were excluded in the EPIC cohort analyses.
Abbreviations: OBR, obesity-related; NOR, non-obesity-related; CI, confidence interval; HR, hazard ratio; BMI, body mass index; MV, multivariable; SD, standard deviation.

Table S23: Comparison of the obesity degree and duration per unit standard deviation, ABACus 2 Consortium.

Outcomes	Number of cancer events	Degree of Obese (per SD)				Duration of Obese (per SD)			
		Age-adjusted HR (95% CI)	I^2	MV-adjusted HR (95% CI)	I^2	Age-adjusted HR (95% CI)	I^2	MV-adjusted HR (95% CI)	I^2
**Men**									
†Total Cancers	85,341	1.01 (0.92,1.12)	0.93	1.01 (0.93,1.11)	0.91	1.02 (1.00,1.04)	0.39	1.02 (1.01,1.03)	0.00
OBR-cancers	12,959	1.05 (0.90,1.21)	0.77	1.04 (0.91,1.20)	0.76	1.07 (0.98,1.17)	0.79	1.08 (0.99,1.17)	0.77
NOR-cancers	64,743	0.99 (0.92,1.07)	0.85	0.99 (0.93,1.05)	0.84	1.00 (0.98,1.02)	0.06	1.01 (0.99,1.02)	0.00
NOR-cancers excluding lung and prostate	26,178	0.99 (0.91,1.07)	0.71	0.99 (0.92,1.07)	0.69	1.03 (1.00,1.07)	0.13	1.04 (0.99,1.08)	0.43
**Specific cancer sites**									
Colorectal	6,037	1.02 (0.93,1.13)	0.60	1.02 (0.94,1.11)	0.56	1.04 (0.97,1.12)	0.38	1.04 (0.97,1.12)	0.38
Pancreas	1,957	0.99 (0.84,1.17)	0.36	1.00 (0.84,1.19)	0.41	1.15 (0.94,1.4)	0.67	1.14 (0.95,1.39)	0.67
Kidney	1,967	0.99 (0.84,1.17)	0.50	1.00 (0.84,1.19)	0.52	1.03 (0.91,1.16)	0.31	1.04 (0.93,1.15)	0.20
Bladder	4,018	0.99 (0.88,1.12)	0.29	1.00 (0.88,1.14)	0.30	1.02 (0.96,1.09)	0.00	1.03 (0.97,1.09)	0.00
Lung	8,559	1.08 (0.87,1.34)	0.84	1.09 (0.9,1.33)	0.82	1.09 (0.99,1.21)	0.68	1.10 (1.03,1.18)	0.42
Prostate	30,006	0.96 (0.93,0.99)	0.06	0.96 (0.93,0.99)	0.00	0.96 (0.92,1.01)	0.49	0.96 (0.93,0.98)	0.17
**Women**									
†Total Cancers	63,732	1.01 (0.97,1.04)	0.80	1.01 (0.97,1.05)	0.80	1.02 (1,1.03)	0.00	1.03 (1.01,1.04)	0.04
OBR-cancers	36,509	1.01 (0.97,1.04)	0.69	1.01 (0.97,1.05)	0.72	1.02 (1.01,1.04)	0.00	1.03 (1.00,1.05)	0.23
NOR-cancers	24,499	1.02 (0.94,1.11)	0.84	1.03 (0.95,1.1)	0.78	1.03 (0.99,1.06)	0.34	1.04 (1.00,1.07)	0.22
NOR-cancers excluding lung	16,352	1.01 (0.96,1.07)	0.66	1.02 (0.97,1.08)	0.61	1.03 (1,1.06)	0.00	1.04 (1.02,1.06)	0.00
**Specific cancer sites**									
Colorectal	6,251	1.03 (0.97,1.09)	0.27	1.03 (0.98,1.09)	0.24	1.05 (1.01,1.08)	0.00	1.04 (1.01,1.08)	0.00
Pancreas	2,019	0.94 (0.78,1.12)	0.52	0.94 (0.79,1.13)	0.53	1.02 (0.89,1.18)	0.6	1.03 (0.89,1.18)	0.58
Kidney	1,270	0.98 (0.86,1.12)	0.58	0.98 (0.86,1.11)	0.50	1.1 (1.03,1.17)	0.00	1.08 (1.02,1.15)	0.00
Lung	8,114	0.99 (0.85,1.16)	0.82	1.00 (0.86,1.15)	0.79	1.01 (0.92,1.1)	0.71	1.03 (0.94,1.13)	0.73
Endometrial	3,931	1.09 (1.00,1.18)	0.85	1.09 (1.01,1.16)	0.79	1.08 (0.95,1.23)	0.88	1.08 (0.96,1.22)	0.84
Ovarian	2,717	1.05 (1.00,1.1)	0.00	1.06 (1.01,1.11)	0.00	1.08 (1.00,1.17)	0.15	1.09 (0.99,1.19)	0.33
Post-menopausal breast cancer	17,582	0.95 (0.91,0.98)	0.23	0.95 (0.91,0.99)	0.31	0.98 (0.96,1.00)	0.00	0.98 (0.96,1.00)	0.00

*Multivariable adjusted models: baseline age, ethnicity, alcohol, smoking, HRT.
† The sum of OBR and NOR cancer does not equal total cancers as non-melanoma skin cancers were excluded in the EPIC cohort analyses.
Degree of overweight is the cumulative sum of the number of BMI units ≥30 kg/m^2
Duration of overweight is the cumulative sum of the duration overweight (BMI ≥ 30 kg/m^2).
Abbreviations: OBR, obesity-related; NOR, non-obesity-related; CI, confidence interval; HR, hazard ratio; BMI, body mass index; MV, multivariable

Table S24: Comparison of the obesity degree and duration per 10 units and per 10 years respectively, ABACus 2 Consortium.

Outcomes	Number of cancer events	Degree of Obese (per 10 units)				Duration of Obese (per 10 years)			
		Age-adjusted HR (95% CI)	I^2	MV-adjusted HR (95% CI)	I^2	Age-adjusted HR (95% CI)	I^2	MV-adjusted HR (95% CI)	I^2
**Men**									
†Total Cancers	85,341	1.06 (0.85,1.32)	0.92	1.05 (0.87,1.28)	0.90	1.02 (1.00,1.03)	0.18	1.02 (1.00,1.04)	0.39
OBR-cancers	12,959	1.13 (0.8,1.6)	0.79	1.13 (0.81,1.58)	0.79	1.06 (0.99,1.14)	0.77	1.06 (0.99,1.14)	0.77
NOR-cancers	64,743	0.99 (0.84,1.16)	0.85	0.98 (0.86,1.12)	0.81	1.00 (0.99,1.02)	0.00	1.00 (0.99,1.01)	0.00
NOR-cancers excluding lung and prostate	26,178	0.97 (0.82,1.15)	0.70	0.98 (0.83,1.16)	0.67	1.03 (1.00,1.06)	0.00	1.03 (0.99,1.07)	0.37
**Specific cancer sites**									
Colorectal	6,037	1.07 (0.79,1.45)	0.61	1.07 (0.80,1.43)	0.60	1.04 (0.97,1.11)	0.48	1.04 (0.98,1.10)	0.35
Pancreas	1,957	0.97 (0.66,1.42)	0.35	0.97 (0.64,1.45)	0.38	1.12 (0.96,1.32)	0.68	1.12 (0.96,1.31)	0.67
Kidney	1,967	1.00 (0.68,1.45)	0.54	0.99 (0.69,1.42)	0.49	1.03 (0.93,1.13)	0.26	1.03 (0.93,1.13)	0.26
Bladder	4,018	0.95 (0.74,1.22)	0.23	0.97 (0.75,1.26)	0.26	1.01 (0.96,1.07)	0.00	1.02 (0.97,1.07)	0.00
Lung	8,559	1.28 (0.71,2.30)	0.87	1.32 (0.75,2.33)	0.87	1.07 (1.00,1.15)	0.54	1.09 (1.03,1.15)	0.32
Prostate	30,006	0.91 (0.84,0.99)	0.21	0.91 (0.87,0.95)	0.00	0.97 (0.94,0.99)	0.37	0.97 (0.94,0.99)	0.24
**Women**									
†Total Cancers	63,732	1.01 (0.96,1.07)	0.81	1.02 (0.96,1.08)	0.84	1.02 (1.00,1.03)	0.15	1.02 (1.00,1.04)	0.41
OBR-cancers	36,509	1.01 (0.96,1.07)	0.69	1.02 (0.96,1.08)	0.74	1.02 (1.00,1.03)	0.00	1.02 (1.01,1.04)	0.02
NOR-cancers	24,499	1.05 (0.88,1.25)	0.85	1.04 (0.89,1.21)	0.82	1.02 (0.99,1.06)	0.56	1.03 (1.00,1.06)	0.24
NOR-cancers excluding lung	16,352	1.02 (0.94,1.12)	0.7	1.03 (0.95,1.12)	0.66	1.03 (0.99,1.06)	0.33	1.03 (1.01,1.05)	0.00
**Specific cancer sites**									
Colorectal	6,251	1.04 (0.92,1.17)	0.50	1.04 (0.94,1.15)	0.37	1.04 (1.02,1.06)	0.00	1.04 (1.02,1.06)	0.00
Pancreas	2,019	0.90 (0.69,1.17)	0.46	0.90 (0.69,1.19)	0.48	1.02 (0.9,1.16)	0.63	1.02 (0.91,1.15)	0.6
Kidney	1,270	0.99 (0.8,1.23)	0.57	0.99 (0.81,1.21)	0.47	1.08 (1.01,1.15)	0.00	1.07 (1.01,1.13)	0.00
Lung	8,114	1.03 (0.74,1.45)	0.8	1.03 (0.76,1.39)	0.77	1.01 (0.93,1.09)	0.72	1.03 (0.95,1.12)	0.73
Endometrial	3,931	1.15 (1.00,1.32)	0.89	1.14 (1.01,1.29)	0.85	1.07 (0.94,1.21)	0.91	1.07 (0.96,1.2)	0.88
Ovarian	2,717	1.07 (0.98,1.16)	0.00	1.07 (0.99,1.16)	0.00	1.07 (0.99,1.15)	0.28	1.07 (1,1.16)	0.31
Post-menopausal breast cancer	17,582	0.91 (0.88,0.94)	0.00	0.91 (0.87,0.95)	0.00	0.98 (0.96,1.00)	0.00	0.98 (0.96,1.01)	0.00

*Multivariable adjusted models: baseline age, ethnicity, alcohol, smoking, HRT.

† The sum of OBR and NOR cancer does not equal total cancers as non-melanoma skin cancers were excluded in the EPIC cohort analyses.

Degree of overweight is the cumulative sum of the number of BMI units ≥ 30 kg/m^2

Duration of overweight is the cumulative sum of the duration overweight (BMI ≥ 30 kg/m^2).

Abbreviations: OBR, obesity-related; NOR, non-obesity-related;CI, confidence interval; HR, hazard ratio; BMI, body mass index; MV, multivariable.

Table S25: Comparison of Harrell's C-statistic of the metrics, ABACus 2 Consortium.

Characteristic	Obese-years	Baseline BMI	Difference in c-statistic between baseline BMI and obese-years	Obese - years with baseline BMI	Difference in c-statistic between obese-years with BMI combined compared with obese-years	Difference in c-statistic between obese-years with BMI combined and BMI	Degree of obesity	Duration of obesity	Difference in c-statistic between duration and degree of obesity
	Harrell's C-statistic(95% CI)								
**Men**									
Total Cancers	0.599 (0.567, 0.630)	0.600 (0.569, 0.630)	-0.000 (-0.001, 0.000)	0.600 (0.568, 0.630)	-0.000 (-0.000, 0.000)	0.000 (-0.000, 0.001)	0.599 (0.567, 0.631)	0.600 (0.569, 0.631)	-0.000 (-0.001, 0.000)
OR-cancers	0.606 (0.574, 0.638)	0.612 (0.579, 0.644)	0.006 (0.002, 0.009)	0.606 (0.563, 0.647)	0.006 (0.003, 0.010)	0.001 (-0.000, 0.002)	0.609 (0.576, 0.642)	0.613 (0.582, 0.642)	0.003 (-0.005, 0.011)
NOR-cancers	0.601 (0.567, 0.634)	0.601 (0.567, 0.634)	0.000 (-0.001, 0.001)	0.602 (0.559, 0.644)	0.001 (-0.000, 0.002)	0.000 (-0.000, 0.001)	0.601 (0.569, 0.633)	0.599 (0.562, 0.634)	0.000 (-0.000, 0.001)
NOR-cancers excluding lung and prostate	0.611 (0.538, 0.679)	0.610 (0.539, 0.677)	-0.001 (-0.003, 0.002)	0.609 (0.534, 0.679)	-0.001 (-0.003, 0.002)	0.000 (-0.001, 0.001)	0.610 (0.539, 0.677)	0.611 (0.538, 0.679)	0.001 (-0.001, 0.003)
**Specific cancer sites**									
Colorectal	0.623 (0.581, 0.662)	0.624 (0.586, 0.661)	0.003 (-0.001, 0.007)	0.626 (0.584, 0.666)	0.004 (0.000, 0.007)	0.001 (-0.001, 0.002)	0.628 (0.575, 0.678)	0.625 (0.584, 0.665)	-0.001 (-0.003, 0.002)
Pancreas	0.616 (0.555, 0.674)	0.620 (0.549, 0.687)	0.002 (-0.003, 0.007)	0.623 (0.556, 0.685)	0.004 (-0.003, 0.011)	0.006 (-0.006, 0.018)	0.619 (0.537, 0.695)	0.628 (0.569, 0.684)	0.005 (-0.004, 0.015)
Kidney	0.577 (0.549, 0.605)	0.598 (0.588, 0.608)	0.013 (-0.004, 0.030)	0.598 (0.588, 0.608)	0.014 (-0.003, 0.030)	0.001 (-0.003 , 0.006)	0.581 (0.553, 0.609)	0.591 (0.566, 0.615)	0.009 (0.001, 0.017)
Bladder	0.700 (0.635, 0.758)	0.700 (0.634, 0.759)	-0.000 (-0.025, 0.024)	0.706 (0.639, 0.765)	0.005 (-0.012, 0.023)	0.006 (-0.011, 0.022)	0.698 (0.633, 0.755)	0.697 (0.633, 0.754)	-0.001 (-0.013, 0.012)
Lung	0.721 (0.695, 0.745)	0.726 (0.698, 0.753)	0.004 (0.000, 0.007)	0.726 (0.699, 0.752)	0.004 (0.002, 0.006)	0.000 (-0.000, 0.001)	0.723 (0.695, 0.749)	0.721 (0.696, 0.744)	-0.000 (-0.002, 0.002)
Prostate	0.598 (0.570, 0.626)	0.599 (0.571, 0.627)	0.000 (-0.001, 0.002)	0.598 (0.568, 0.628)	0.000 (-0.001, 0.001)	-0.000 (-0.001, 0.000)	0.598 (0.567, 0.629)	0.600 (0.572, 0.627)	-0.000 (-0.001, 0.001)
**Women**									
Total Cancers	0.579 (0.557, 0.600)	0.581 (0.561, 0.601)	0.001 (-0.000, 0.001)	0.579 (0.554, 0.603)	0.001 (-0.000, 0.001)	0.000 (-0.000, 0.000)	0.582 (0.561, 0.603)	0.581 (0.561, 0.602)	-0.000 (-0.001, 0.001)
OR-cancers	0.559 (0.528, 0.590)	0.569 (0.542, 0.597)	0.007 (-0.000, 0.014)	0.567 (0.539, 0.594)	0.007 (0.001, 0.013)	0.000 (-0.000, 0.000)	0.564 (0.534, 0.593)	0.566 (0.538, 0.594)	-0.000 (-0.002, 0.001)
NOR-cancers	0.640 (0.581, 0.696)	0.642 (0.579, 0.699)	0.001 (-0.002, 0.004)	0.641 (0.578, 0.699)	0.002 (0.000, 0.004)	0.000 (-0.000, 0.001)	0.641 (0.582, 0.696)	0.641 (0.581, 0.696)	0.001 (0.000, 0.002)
NOR-cancers excluding lung	0.588 (0.542, 0.633)	0.590 (0.543, 0.636)	0.000 (-0.002, 0.003)	0.590 (0.542, 0.636)	0.002 (-0.001, 0.004)	0.000 (-0.000, 0.000)	0.589 (0.541, 0.635)	0.589 (0.543, 0.634)	0.002 (-0.003, 0.006)
**Specific cancer sites**									
Colorectal	0.622 (0.546, 0.693)	0.632 (0.577, 0.684)	-0.000 (-0.002, 0.002)	0.633 (0.575, 0.687)	-0.000 (-0.001, 0.001)	-0.000 (-0.000, 0.000)	0.627 (0.561, 0.688)	0.629 (0.566, 0.688)	-0.000 (-0.001, 0.001)
Pancreas	0.633 (0.584, 0.679)	0.634 (0.583, 0.683)	0.001 (-0.001, 0.003)	0.636 (0.585, 0.685)	0.000 (-0.001, 0.002)	-0.000 (-0.002, 0.002)	0.635 (0.584, 0.683)	0.632 (0.584, 0.678)	-0.001 (-0.005, 0.004)
Kidney	0.591 (0.561, 0.620)	0.626 (0.606, 0.646)	0.019 (0.005, 0.032)	0.645 (0.607, 0.681)	0.018 (0.004, 0.032)	-0.001 (-0.003, 0.001)	0.600 (0.572, 0.628)	0.614 (0.594, 0.633)	0.011 (-0.001, 0.024)
Lung	0.633 (0.584, 0.679)	0.634 (0.583, 0.683)	0.002 (-0.001, 0.006)	0.636 (0.585, 0.685)	0.002 (-0.001, 0.005)	0.000 (-0.000, 0.001)	0.635 (0.584, 0.683)	0.632 (0.584, 0.678)	-0.000 (-0.003, 0.003)
Endometrial	0.591 (0.561, 0.620)	0.626 (0.606, 0.646)	0.022 (0.011, 0.034)	0.645 (0.607, 0.681)	0.020 (0.011, 0.028)	0.000 (-0.000, 0.001)	0.600 (0.572, 0.628)	0.614 (0.594, 0.633)	-0.007 (-0.011, -0.002)
Ovarian	0.743 (0.713, 0.772)	0.746 (0.715, 0.775)	-0.001 (-0.005, 0.003)	0.748 (0.715, 0.778)	0.001 (-0.003, 0.004)	0.001 (-0.002, 0.004)	0.745 (0.713, 0.775)	0.744 (0.713, 0.772)	0.001 (-0.003, 0.006)
Post-menopausal breast cancer	0.595 (0.560, 0.629)	0.626 (0.581, 0.669)	0.004 (0.002, 0.007)	0.627 (0.580, 0.671)	0.005 (0.002, 0.007)	0.000 (-0.001, 0.001)	0.616 (0.571, 0.659)	0.613 (0.579, 0.645)	-0.000 (-0.001, 0.001)

Key: Green – significant difference in C-statistic.
*All models were multivariable adjusted, including baseline age, ethnicity, alcohol, smoking, HRT.
Abbreviations: SE, standard error; OR, obesity-related; NOR, non-obesity-related; BMI, body mass index.

b) Findings from analysis using predicted BMI from participants with at least 1 measured BMI readings

Analysis of overweight-years exposure

Table S26: Hazard ratio of cancers per standard deviation of overweight-years and BMI, ABACus 2 Consortium.

Outcomes	Number of cancer events	Overweight-years (per SD)				BMI (per SD)			
		Age-adjusted HR (95% CI)	I^2	MV-adjusted HR (95% CI)	I^2	Age-adjusted HR (95% CI)	I^2	MV-adjusted HR (95% CI)	I^2
**Men**									
†Total Cancers	130,704	1.02 (0.99,1.05)	0.75	1.02 (0.99,1.05)	0.75	1.02 (0.99,1.04)	0.73	1.01 (0.98,1.04)	0.72
OBR-cancers	23,060	1.15 (1.15,1.16)	0.00	1.15 (1.14,1.16)	0.00	1.17 (1.16,1.18)	0.00	1.17 (1.16,1.18)	0.00
NOR-cancers	72,342	0.97 (0.94,1.00)	0.74	0.97 (0.94,1.00)	0.67	0.96 (0.94,0.99)	0.60	0.96 (0.93,0.99)	0.73
NOR-cancers excluding lung and prostate	9,663	1.05 (1.01,1.09)	0.31	1.04 (1.00,1.08)	0.2	1.05 (1.02,1.08)	0.00	1.04 (1.01,1.07)	0.00
**Specific cancer sites**									
Colorectal	11,063	1.13 (1.08,1.18)	0.63	1.13 (1.08,1.18)	0.6	1.15 (1.11,1.19)	0.18	1.15 (1.11,1.19)	0.32
Pancreas	3,351	1.1 (1.05,1.15)	0.00	1.10 (1.05,1.15)	0.00	1.09 (1.03,1.15)	0.10	1.1 (1.04,1.16)	0.03
Kidney	3,398	1.2 (1.14,1.26)	0.13	1.19 (1.13,1.25)	0.18	1.24 (1.13,1.35)	0.44	1.23 (1.13,1.34)	0.42
Bladder	2,787	1.08 (0.99,1.18)	0.12	1.07 (0.97,1.18)	0.21	1.08 (1.01,1.16)	0.00	1.07 (0.99,1.15)	0.00
Lung	14,545	0.94 (0.89,0.99)	0.59	0.92 (0.87,0.98)	0.68	0.86 (0.75,0.99)	0.90	0.84 (0.75,0.94)	0.90
Prostate	48,116	0.96 (0.91,1.02)	0.77	0.96 (0.91,1.01)	0.75	0.97 (0.91,1.04)	0.89	0.97 (0.92,1.02)	0.80
**Women**									
†Total Cancers	98,690	1.04 (1.03,1.05)	0.00	1.05 (1.04,1.07)	0.53	1.05 (1.01,1.08)	0.77	1.06 (1.02,1.10)	0.80
OBR-cancers	55,712	1.09 (1.05,1.13)	0.86	1.10 (1.06,1.14)	0.87	1.11 (1.08,1.14)	0.76	1.12 (1.08,1.16)	0.86
NOR-cancers	26,718	0.94 (0.89,0.99)	0.87	0.96 (0.92,1.01)	0.82	0.91 (0.83,1.01)	0.94	0.94 (0.84,1.05)	0.93
NOR-cancers excluding lung	13,736	1.01 (0.96,1.05)	0.44	1.02 (0.99,1.05)	0.29	0.99 (0.9,1.09)	0.80	1.01 (0.91,1.13)	0.79
**Specific cancer sites**									
Colorectal	10,026	1.08 (1.05,1.11)	0.07	1.08 (1.05,1.10)	0.00	1.09 (1.05,1.14)	0.38	1.08 (1.05,1.12)	0.15
Pancreas	3,172	1.02 (0.95,1.10)	0.59	1.02 (0.96,1.08)	0.35	1.01 (0.96,1.07)	0.08	1.01 (0.98,1.05)	0.00
Kidney	2,069	1.23 (1.17,1.29)	0.11	1.22 (1.15,1.29)	0.36	1.31 (1.18,1.45)	0.84	1.30 (1.18,1.44)	0.84
Lung	12,894	0.87 (0.84,0.90)	0.27	0.90 (0.87,0.93)	0.16	0.84 (0.80,0.89)	0.66	0.87 (0.83,0.90)	0.41
Endometrial	6,138	1.30 (1.22,1.38)	0.82	1.31 (1.25,1.37)	0.62	1.36 (1.21,1.53)	0.93	1.4 (1.21,1.62)	0.94
Ovarian	4,092	1.02 (0.95,1.10)	0.68	1.03 (0.96,1.11)	0.60	1.00 (0.91,1.10)	0.77	1.02 (0.93,1.12)	0.72
Post-menopausal breast cancer	25,512	1.01 (1.00,1.03)	0.10	1.03 (1,1.06)	0.40	1.05 (1.01,1.09)	0.62	1.07 (1.02,1.12)	0.72

*Multivariable adjusted models: baseline age, ethnicity, alcohol, smoking, HRT.
† The sum of OBR and NOR cancer does not equal total cancers as non-melanoma skin cancers were excluded in the EPIC cohort analyses.
Abbreviations: OBR, obesity-related; NOR, non-obesity-related;CI, confidence interval; HR, hazard ratio; BMI, body mass index; MV, multivariable; SD, standard deviation.

Table S27: Hazard ratio of specific cancers per 100 overweight-years and per 5-unit baseline BMI, ABACus 2 Consortium.

Outcomes	Number of cancer events	Overweight-years (per 100 kg-years/m^2)				BMI (per 5 units)			
		Age-adjusted HR (95% CI)	I^2	MV-adjusted HR (95% CI)	I^2	Age-adjusted HR (95% CI)	I^2	MV-adjusted HR (95% CI)	I^2
**Men**									
†Total Cancers	130,704	1.03 (0.99,1.08)	0.83	1.03 (0.98,1.08)	0.86	1.02 (0.99,1.05)	0.67	1.01 (0.98,1.05)	0.68
OBR-cancers	23,060	1.21 (1.19,1.22)	0.00	1.22 (1.19,1.24)	0.00	1.2 (1.19,1.21)	0.00	1.2 (1.19,1.21)	0.00
NOR-cancers	72,342	0.95 (0.90,1.00)	0.58	0.94 (0.92,0.97)	0.33	0.95 (0.89,1.02)	0.86	0.95 (0.89,1.01)	0.71
NOR-cancers excluding lung and prostate	9,663	1.06 (0.98,1.15)	0.40	1.05 (0.97,1.15)	0.51	1.05 (1.01,1.1)	0.00	1.04 (0.98,1.11)	0.13
**Specific cancer sites**									
Colorectal	11,063	1.26 (0.85,1.86)	0.81	1.26 (0.86,1.83)	0.80	1.18 (1.10,1.26)	0.47	1.18 (1.10,1.26)	0.42
Pancreas	3,351	1.12 (1.01,1.25)	0.00	1.13 (1.03,1.23)	0.00	1.09 (0.97,1.23)	0.00	1.09 (0.95,1.25)	0.09
Kidney	3,398	1.27 (1.16,1.39)	0.08	1.26 (1.15,1.38)	0.09	1.26 (1.15,1.39)	0.35	1.26 (1.16,1.38)	0.21
Bladder	2,787	1.12 (0.91,1.39)	0.00	1.12 (0.88,1.43)	0.00	1.11 (1.00,1.23)	0.00	1.10 (0.96,1.27)	0.00
Lung	14,545	0.93 (0.81,1.07)	0.67	0.91 (0.81,1.04)	0.63	0.85 (0.64,1.13)	0.89	0.83 (0.66,1.05)	0.90
Prostate	48,116	0.95 (0.81,1.11)	0.79	0.93 (0.83,1.04)	0.68	0.96 (0.83,1.11)	0.86	0.95 (0.85,1.07)	0.72
**Women**									
†Total Cancers	98,690	1.05 (1.03,1.06)	0.62	1.06 (1.04,1.08)	0.64	1.04 (1.01,1.07)	0.83	1.05 (1.02,1.09)	0.85
OBR-cancers	55,712	1.10 (1.09,1.11)	0.00	1.11 (1.09,1.12)	0.00	1.10 (1.07,1.13)	0.84	1.11 (1.08,1.14)	0.84
NOR-cancers	26,718	0.93 (0.87,1.00)	0.9	0.95 (0.90,1.02)	0.87	0.91 (0.82,1.01)	0.94	0.94 (0.84,1.05)	0.94
NOR-cancers excluding lung	13,736	1.01 (0.96,1.06)	0.51	1.02 (1.00,1.05)	0.23	0.99 (0.90,1.10)	0.83	1.01 (0.91,1.12)	0.77
**Specific cancer sites**									
Colorectal	10,026	1.09 (1.07,1.11)	0.00	1.09 (1.07,1.10)	0.00	1.08 (1.05,1.11)	0.13	1.08 (1.05,1.10)	0.07
Pancreas	3,172	1.02 (0.94,1.10)	0.59	1.02 (0.95,1.09)	0.37	1.01 (0.96,1.06)	0.02	1.01 (0.98,1.04)	0.00
Kidney	2,069	1.24 (1.20,1.30)	0.00	1.24 (1.20,1.27)	0.00	1.28 (1.17,1.40)	0.84	1.27 (1.16,1.39)	0.85
Lung	12,894	0.86 (0.80,0.92)	0.84	0.89 (0.85,0.94)	0.66	0.85 (0.79,0.92)	0.79	0.88 (0.83,0.93)	0.69
Endometrial	6,138	1.37 (1.23,1.52)	0.96	1.37 (1.25,1.52)	0.94	1.34 (1.19,1.50)	0.95	1.36 (1.19,1.56)	0.96
Ovarian	4,092	1.03 (0.93,1.14)	0.69	1.05 (0.95,1.16)	0.68	1.00 (0.91,1.10)	0.75	1.02 (0.93,1.12)	0.70
Post-menopausal breast cancer	25,512	1.02 (0.99,1.05)	0.24	1.04 (0.99,1.09)	0.54	1.05 (1.01,1.10)	0.67	1.07 (1.02,1.12)	0.74

*Multivariable adjusted models: baseline age, ethnicity, alcohol, smoking, HRT.
† The sum of OBR and NOR cancer does not equal total cancers as non-melanoma skin cancers were excluded in the EPIC cohort analyses.
Abbreviations: OBR, obesity-related; NOR, non-obesity-related;CI, confidence interval; HR, hazard ratio; BMI, body mass index; MV, multivariable; SD, standard deviation.

Table S28: Hazard ratios of cancers per standard deviation overweight degree and duration, ABACus 2 Consortium.

Outcomes	Number of cancer events	Degree of Overweight (per SD)				Duration of Overweight (per SD)			
		Age-adjusted HR (95% CI)	I^2	MV-adjusted HR (95% CI)	I^2	Age-adjusted HR (95% CI)	I^2	MV-adjusted HR (95% CI)	I^2
**Men**									
†Total Cancers	130,704	1.03 (0.99,1.06)	0.51	1.03 (1,1.07)	0.49	1 (0.97,1.04)	0.59	1 (0.96,1.03)	0.00
OBR-cancers	23,060	1.07 (1.04,1.11)	0.00	1.08 (1.05,1.12)	0.00	1.04 (1,1.08)	0.17	1.02 (0.94,1.11)	0.00
NOR-cancers	72,342	1.00 (0.97,1.03)	0.19	1.00 (0.97,1.03)	0.15	0.99 (0.96,1.02)	0.44	0.99 (0.95,1.02)	0.00
NOR-cancers excluding lung and prostate	9,663	1.03 (0.96,1.11)	0.24	1.05 (0.97,1.13)	0.30	1.01 (0.91,1.14)	0.69	0.99 (0.88,1.11)	0.00
**Specific cancer sites**									
Colorectal	11,063	1.09 (0.96,1.24)	0.62	1.09 (0.97,1.23)	0.57	1.01 (0.98,1.05)	0.00	1.04 (0.96,1.12)	0.00
Pancreas	3,351	1.09 (0.98,1.21)	0.07	1.08 (0.97,1.21)	0.15	1.06 (0.98,1.14)	0.00	1.01 (0.87,1.16)	0.00
Kidney	3,398	1.02 (0.92,1.13)	0.00	1.02 (0.92,1.14)	0.00	1.10 (1,1.21)	0.24	0.96 (0.74,1.27)	0.00
Bladder	2,787	1.04 (0.98,1.1)	0.00	1.06 (1,1.12)	0.00	0.99 (0.87,1.13)	0.58	0.99 (0.86,1.13)	0.00
Lung	14,545	1.10 (0.99,1.22)	0.49	1.13 (1.08,1.19)	0.00	0.98 (0.93,1.04)	0.25	0.98 (0.89,1.08)	0.00
Prostate	48,116	0.96 (0.92,1.02)	0.51	0.96 (0.92,1)	0.29	0.98 (0.96,1.00)	0.00	0.99 (0.94,1.04)	0.00
**Women**									
†Total Cancers	98,690	1.03 (0.99,1.07)	0.71	1.04 (1.00,1.07)	0.68	1.02 (1.00,1.04)	0.43	1.03 (1.01,1.04)	0.17
OBR-cancers	55,712	1.03 (0.99,1.08)	0.76	1.04 (0.99,1.08)	0.82	1.03 (1.00,1.06)	0.69	1.03 (1.00,1.07)	0.76
NOR-cancers	26,718	1.00 (0.92,1.07)	0.79	1.01 (0.94,1.09)	0.78	0.99 (0.91,1.09)	0.86	1.01 (0.92,1.11)	0.87
NOR-cancers excluding lung	13,736	1.01 (0.93,1.10)	0.64	1.02 (0.93,1.11)	0.72	1.02 (0.93,1.11)	0.73	1.02 (0.94,1.11)	0.68
**Specific cancer sites**									
Colorectal	10,026	1.04 (1.02,1.07)	0.00	1.04 (1.02,1.07)	0.00	1.06 (1.03,1.10)	0.00	1.06 (1.02,1.10)	0.01
Pancreas	3,172	1.03 (0.93,1.13)	0.40	1.03 (0.93,1.14)	0.46	1.10 (1.01,1.21)	0.31	1.11 (1.02,1.20)	0.21
Kidney	2,069	1.06 (0.96,1.17)	0.40	1.07 (0.98,1.17)	0.30	1.16 (1.03,1.3)	0.5	1.15 (1.03,1.28)	0.4
Lung	12,894	0.96 (0.90,1.03)	0.56	0.99 (0.93,1.05)	0.50	0.96 (0.88,1.06)	0.78	0.99 (0.89,1.11)	0.83
Endometrial	6,138	1.15 (1.01,1.3)	0.86	1.14 (1.01,1.29)	0.85	1.11 (0.90,1.37)	0.95	1.10 (0.90,1.36)	0.95
Ovarian	4,092	1.09 (1.03,1.15)	0.00	1.09 (1.03,1.15)	0.00	1.02 (0.93,1.12)	0.42	1.02 (0.92,1.12)	0.47
Post-menopausal breast cancer	25,512	0.97 (0.91,1.04)	0.84	0.98 (0.92,1.05)	0.87	0.99 (0.94,1.03)	0.73	1.00 (0.95,1.05)	0.83

*Multivariable adjusted models: baseline age, ethnicity, alcohol, smoking, HRT.
† The sum of OBR and NOR cancer does not equal total cancers as non-melanoma skin cancers were excluded in the EPIC cohort analyses.
Degree of overweight is the cumulative sum of the number of BMI units ≥ 25 kg/m^2
Duration of overweight is the cumulative sum of the duration overweight (BMI ≥ 25 kg/m^2).
Abbreviations: OBR, obesity-related; NOR, non-obesity-related; CI, confidence interval; HR, hazard ratio; BMI, body mass index; MV, multivariable.

Table S29: Comparison of the overweight degree and duration per 10 units and per 10 years respectively, ABACus 2 Consortium.

Outcomes	Number of cancer events	Degree of Overweight (per 10 units)				Duration of Overweight (per 10 years)			
		Age-adjusted HR (95% CI)	I^2	MV-adjusted HR (95% CI)	I^2	Age-adjusted HR (95% CI)	I^2	MV-adjusted HR (95% CI)	I^2
**Men**									
†Total Cancers	130,704	1.00 (1.00,1.01)	0.94	1.00 (1.00,1.01)	0.94	1.00 (0.99,1.02)	0.29	1.00 (0.99,1.02)	0.28
OBR-cancers	23,060	1.01 (1.00,1.03)	0.82	1.01 (1.00,1.03)	0.82	1.04 (0.89,1.22)	0.91	1.04 (0.92,1.18)	0.87
NOR-cancers	72,342	1.00 (1.00,1.00)	0.00	1.00 (1.00,1.00)	0.00	0.99 (0.97,1.00)	0.00	0.98 (0.95,1.01)	0.58
NOR-cancers excluding lung and prostate	9,663	1.01 (0.98,1.03)	0.51	1.01 (0.98,1.03)	0.51	1.01 (0.92,1.12)	0.49	1.01 (0.93,1.11)	0.48
**Specific cancer sites**									
Colorectal	11,063	1.02 (0.99,1.05)	0.80	1.02 (0.99,1.05)	0.80	1.04 (0.95,1.14)	0.77	1.04 (0.95,1.14)	0.76
Pancreas	3,351	1.01 (0.99,1.04)	0.47	1.01 (0.99,1.04)	0.46	1.04 (0.98,1.11)	0.00	1.04 (0.99,1.11)	0.00
Kidney	3,398	1.02 (0.98,1.06)	0.72	1.02 (0.98,1.06)	0.72	1.08 (0.78,1.50)	0.71	1.08 (0.78,1.49)	0.69
Bladder	2,787	1.01 (1.01,1.02)	0.00	1.01 (0.99,1.02)	0.05	1.01 (0.93,1.11)	0.42	1.01 (0.91,1.12)	0.57
Lung	14,545	1.00 (0.97,1.03)	0.84	1.01 (0.95,1.07)	0.96	0.94 (0.84,1.06)	0.86	0.95 (0.81,1.12)	0.93
Prostate	48,116	1.00 (1.00,1.00)	0.00	1.00 (1.00,1.00)	0.00	0.99 (0.98,1.00)	0.00	0.99 (0.98,1.01)	0.00
**Women**									
†Total Cancers	98,690	1.00 (1.00,1.01)	0.95	1.00 (1.00,1.01)	0.95	1.01 (1.00,1.03)	0.16	1.02 (1.00,1.03)	0.55
OBR-cancers	55,712	1.00 (0.99,1.01)	0.92	1.00 (0.99,1.01)	0.92	1.02 (1.00,1.05)	0.66	1.03 (1.00,1.06)	0.81
NOR-cancers	26,718	1.00 (0.98,1.01)	0.86	1.00 (0.98,1.01)	0.86	0.99 (0.90,1.08)	0.90	0.99 (0.90,1.09)	0.90
NOR-cancers excluding lung	13,736	1.00 (0.98,1.02)	0.81	1.00 (0.98,1.02)	0.81	1.00 (0.92,1.08)	0.76	1.00 (0.93,1.08)	0.69
Colorectal	10,026	1.00 (0.99,1.01)	0.64	1.00 (0.99,1.01)	0.64	1.04 (1.02,1.07)	0.00	1.04 (1.02,1.06)	0.00
Pancreas	3,172	1.01 (0.99,1.03)	0.60	1.01 (0.99,1.03)	0.6	1.07 (0.97,1.18)	0.55	1.07 (0.98,1.17)	0.47
Kidney	2,069	1.01 (0.99,1.02)	0.51	1.01 (1.00,1.02)	0.49	1.11 (0.98,1.25)	0.71	1.11 (0.99,1.24)	0.68
Lung	12,894	1.00 (0.99,1.00)	0.00	1.00 (0.99,1.01)	0.76	0.97 (0.88,1.06)	0.83	0.98 (0.90,1.07)	0.83
Endometrial	6,138	1.02 (0.99,1.05)	0.95	1.02 (0.99,1.05)	0.94	1.11 (0.92,1.34)	0.97	1.11 (0.92,1.34)	0.97
Ovarian	4,092	1.01 (1.00,1.02)	0.00	1.01 (1.00,1.02)	0.58	1.03 (0.97,1.09)	0.27	1.03 (0.97,1.09)	0.27
Post-menopausal breast cancer	25,512	1.00 (0.99,1.01)	0.82	1.00 (0.98,1.02)	0.95	0.99 (0.94,1.05)	0.85	1.00 (0.95,1.06)	0.84

*Multivariable adjusted models: baseline age, ethnicity, alcohol, smoking, HRT.
† The sum of OBR and NOR cancer does not equal total cancers as non-melanoma skin cancers were excluded in the EPIC cohort analyses.
Degree of overweight is the cumulative sum of the number of BMI units ≥ 25 kg/m^2
Duration of overweight is the cumulative sum of the duration overweight (BMI ≥ 25 kg/m^2).
Abbreviations: OBR, obesity-related; NOR, non-obesity-related; CI, confidence interval; HR, hazard ratio; BMI, body mass index; MV, multivariable.

Table S30: Comparison of Harrell's C-statistic of the metrics, ABACus 2 Consortium

Characteristic	Harrell's C-statistic (95% CI)								
	**Overweight-years**	**BMI**	**Difference in c-statistic between BMI and overweight-years**	**Overweight-years with BMI**	**Difference in c-statistic: overweight-years with BMI combined compared with overweight-years**	**Difference in c-statistic between overweight-years with BMI combined and BMI**	**Degree of overweight**	**Duration of overweight**	**Difference in c-statistic between duration and degree of overweight**
**Men**									
Total Cancers	0.596 (0.559, 0.632)	0.596 (0.559, 0.631)	-0.001 (-0.004, 0.002)	0.592 (0.546, 0.637)	0.000 (-0.002, 0.003)	0.001 (-0.002, 0.004)	0.596 (0.560, 0.631)	0.596 (0.559, 0.631)	-0.001 (-0.004, 0.002)
OBR-cancers	0.619 (0.554, 0.680)	0.619 (0.555, 0.679)	0.000 (-0.004, 0.004)	0.619 (0.555, 0.680)	0.002 (-0.001, 0.005)	0.000 (-0.000, 0.001)	0.603 (0.583, 0.622)	0.617 (0.551, 0.678)	-0.001 (-0.004, 0.003)
NOR-cancers	0.609 (0.553, 0.663)	0.609 (0.551, 0.664)	0.001 (-0.003, 0.004)	0.617 (0.560, 0.672)	0.001 (-0.001, 0.002)	-0.000 (-0.003, 0.003)	0.609 (0.552, 0.663)	0.608 (0.550, 0.664)	0.000 (-0.003, 0.003)
NOR-cancers excluding lung and prostate	0.621 (0.545, 0.692)	0.621 (0.544, 0.692)	0.000 (-0.002, 0.002)	0.622 (0.545, 0.693)	-0.000 (-0.001, 0.001)	0.000 (-0.002, 0.002)	0.621 (0.544, 0.692)	0.621 (0.547, 0.690)	0.000 (-0.001, 0.001)
**Specific cancer sites**									
Colorectal	0.633 (0.583, 0.679)	0.627 (0.579, 0.673)	0.001 (-0.002, 0.003)	0.633 (0.593, 0.672)	0.001 (-0.002, 0.004)	0.001 (-0.001, 0.003)	0.630 (0.587, 0.670)	0.629 (0.585, 0.671)	-0.000 (-0.003, 0.003)
Pancreas	0.608 (0.549, 0.664)	0.608 (0.551, 0.662)	-0.001 (-0.003, 0.002)	0.613 (0.568, 0.656)	0.001 (-0.001, 0.004)	0.002 (-0.002, 0.006)	0.608 (0.548, 0.665)	0.607 (0.548, 0.663)	0.001 (-0.002, 0.003)
Kidney	0.588 (0.564, 0.612)	0.592 (0.564, 0.618)	0.003 (-0.003, 0.009)	0.594 (0.569, 0.617)	0.004 (-0.002, 0.009)	0.001 (-0.002, 0.004)	0.587 (0.565, 0.610)	0.590 (0.569, 0.611)	0.004 (-0.002, 0.010)
Bladder	0.675 (0.625, 0.722)	0.675 (0.624, 0.722)	0.000 (-0.002, 0.002)	0.679 (0.630, 0.725)	-0.000 (-0.002, 0.001)	-0.000 (-0.001, 0.001)	0.674 (0.622, 0.722)	0.674 (0.624, 0.720)	0.000 (-0.001, 0.001)
Lung	0.721 (0.692, 0.748)	0.725 (0.694, 0.755)	0.005 (0.000, 0.010)	0.726 (0.692, 0.758)	0.005 (0.001, 0.009)	0.001 (-0.001, 0.004)	0.721 (0.694, 0.747)	0.724 (0.698, 0.750)	0.001 (-0.002, 0.004)
Prostate	0.615 (0.561, 0.666)	0.607 (0.553, 0.658)	-0.001 (-0.005, 0.003)	0.608 (0.535, 0.676)	-0.000 (-0.004, 0.003)	0.000 (-0.003, 0.004)	0.606 (0.550, 0.661)	0.604 (0.547, 0.658)	-0.002 (-0.006, 0.002)
**Women**									
Total Cancers	0.580 (0.559, 0.600)	0.578 (0.556, 0.599)	0.000 (-0.004, 0.004)	0.575 (0.549, 0.601)	-0.001 (-0.005, 0.004)	-0.001 (-0.005, 0.004)	0.575 (0.556, 0.595)	0.575 (0.559, 0.591)	-0.001 (-0.005, 0.003)
OBR-cancers	0.561 (0.538, 0.584)	0.565 (0.543, 0.587)	0.003 (-0.001, 0.006)	0.568 (0.546, 0.590)	0.002 (0.000, 0.004)	0.000 (-0.004, 0.005)	0.562 (0.538, 0.585)	0.567 (0.547, 0.587)	-0.000 (-0.001, 0.000)
NOR-cancers	0.644 (0.592, 0.693)	0.645 (0.591, 0.695)	0.001 (-0.002, 0.005)	0.643 (0.590, 0.693)	0.002 (-0.002, 0.006)	0.001 (-0.001, 0.003)	0.644 (0.590, 0.694)	0.645 (0.591, 0.695)	0.001 (0.000, 0.002)
NOR-cancers excluding lung	0.597 (0.551, 0.642)	0.599 (0.552, 0.644)	0.000 (-0.003, 0.003)	0.602 (0.559, 0.643)	0.000 (-0.003, 0.003)	-0.000 (-0.003, 0.002)	0.598 (0.551, 0.642)	0.597 (0.550, 0.642)	-0.001 (-0.003, 0.001)
**Specific cancer sites**									
Colorectal	0.625 (0.586, 0.662)	0.628 (0.594, 0.660)	0.002 (-0.002, 0.007)	0.611 (0.556, 0.663)	0.002 (-0.002, 0.005)	-0.000 (-0.005, 0.004)	0.625 (0.587, 0.662)	0.626 (0.591, 0.660)	0.001 (-0.004, 0.007)
Pancreas	0.636 (0.588, 0.681)	0.635 (0.588, 0.681)	0.001 (-0.001, 0.004)	0.639 (0.590, 0.685)	0.002 (-0.001, 0.004)	0.000 (-0.001, 0.001)	0.637 (0.589, 0.681)	0.634 (0.588, 0.677)	-0.001 (-0.004, 0.002)
Kidney	0.609 (0.594, 0.625)	0.623 (0.604, 0.642)	0.012 (0.003, 0.021)	0.624 (0.599, 0.648)	0.010 (0.003, 0.018)	-0.001 (-0.003, 0.001)	0.610 (0.595, 0.625)	0.622 (0.604, 0.640)	0.011 (-0.000, 0.023)
Lung	0.740 (0.708, 0.770)	0.743 (0.709, 0.773)	0.003 (-0.000, 0.006)	0.742 (0.711, 0.771)	0.002 (-0.002, 0.007)	0.001 (-0.001, 0.002)	0.740 (0.710, 0.769)	0.742 (0.711, 0.771)	0.001 (-0.001, 0.002)
Endometrial	0.608 (0.561, 0.652)	0.614 (0.557, 0.668)	0.008 (-0.004, 0.020)	0.621 (0.565, 0.675)	0.011 (0.000, 0.022)	0.000 (-0.007, 0.007)	0.592 (0.562, 0.623)	0.588 (0.552, 0.622)	-0.006 (-0.019, 0.007)
Ovarian	0.558 (0.529, 0.587)	0.559 (0.532, 0.585)	0.001 (-0.003, 0.005)	0.561 (0.533, 0.589)	0.001 (-0.002, 0.005)	0.001 (-0.003, 0.004)	0.557 (0.527, 0.587)	0.559 (0.529, 0.588)	-0.000 (-0.005, 0.005)
Post-menopausal breast cancer	0.596 (0.512, 0.674)	0.599 (0.518, 0.675)	0.003 (0.000, 0.006)	0.593 (0.500, 0.679)	0.002 (-0.001, 0.006)	-0.001 (-0.002, 0.001)	0.596 (0.513, 0.674)	0.592 (0.513, 0.666)	-0.001 (-0.003, 0.001)

*All models were multivariable adjusted, including baseline age, ethnicity, alcohol, smoking, HRT.
Key: Green – significant difference in C-statistic.
Abbreviations: SE, standard error; OBR, obesity-related; NOR, non-obesity-related; BMI, body mass index; CI, confidence interval; MV, multivariable-adjusted.

b) Analysis of obese-years exposure

Table S31: Hazard ratios of cancers per standard deviation obese-years and BMI, ABACus 2 Consortium.

Outcomes	Number of cancer events	Obese-years (per SD)				BMI (per SD)			
		Age-adjusted HR (95% CI)	I^2	MV-adjusted HR (95% CI)	I^2	Age-adjusted HR (95% CI)	I^2	MV-adjusted HR (95% CI)	I^2
**Men**									
†Total Cancers	130,704	1.02 (0.99,1.05)	0.78	1.02 (0.98,1.06)	0.86	1.02 (0.99,1.04)	0.73	1.01 (0.98,1.04)	0.72
OBR-cancers	23,060	1.09 (1.08,1.10)	0.00	1.09 (1.08,1.10)	0.00	1.17 (1.16,1.18)	0.00	1.17 (1.16,1.18)	0.00
NOR-cancers	72,342	0.98 (0.94,1.03)	0.83	0.98 (0.94,1.02)	0.76	0.96 (0.94,0.99)	0.60	0.96 (0.93,0.99)	0.73
NOR-cancers excluding lung and prostate	9,663	1.03 (0.97,1.10)	0.71	1.03 (0.97,1.10)	0.70	1.05 (1.02,1.08)	0.00	1.04 (1.01,1.07)	0.00
**Specific cancer sites**									
Colorectal	11,063	1.10 (1.00,1.20)	0.69	1.10 (1.00,1.20)	0.68	1.15 (1.11,1.19)	0.18	1.15 (1.11,1.19)	0.32
Pancreas	3,351	1.06 (1.01,1.11)	0.05	1.06 (1.01,1.11)	0.04	1.09 (1.03,1.15)	0.10	1.10 (1.04,1.16)	0.03
Kidney	3,398	1.11 (1.08,1.14)	0.00	1.11 (1.08,1.15)	0.00	1.24 (1.13,1.35)	0.44	1.23 (1.13,1.34)	0.42
Bladder	2,787	1.06 (0.97,1.15)	0.42	1.06 (0.97,1.16)	0.43	1.08 (1.01,1.16)	0.00	1.07 (0.99,1.15)	0.00
Lung	14,545	0.98 (0.95,1.01)	0.11	0.97 (0.94,1.01)	0.14	0.86 (0.75,0.99)	0.9	0.84 (0.75,0.94)	0.90
Prostate	48,116	0.97 (0.93,1.01)	0.64	0.96 (0.92,1.00)	0.58	0.97 (0.91,1.04)	0.89	0.97 (0.92,1.02)	0.80
**Women**									
†Total Cancers	98,690	1.04 (1.03,1.05)	0.20	1.04 (1.02,1.06)	0.51	1.05 (1.01,1.08)	0.77	1.06 (1.02,1.10)	0.80
OBR-cancers	55,712	1.07 (1.03,1.10)	0.85	1.07 (1.04,1.10)	0.80	1.11 (1.08,1.14)	0.76	1.12 (1.08,1.16)	0.86
NOR-cancers	26,718	0.96 (0.92,1.00)	0.74	0.97 (0.93,1.02)	0.73	0.91 (0.83,1.01)	0.94	0.94 (0.84,1.05)	0.93
NOR-cancers excluding lung	13,736	1.01 (0.97,1.04)	0.25	1.02 (1.00,1.04)	0.00	0.99 (0.90,1.09)	0.80	1.01 (0.91,1.13)	0.79
**Specific cancer sites**									
Colorectal	10,026	1.06 (1.05,1.08)	0.00	1.06 (1.04,1.07)	0.00	1.09 (1.05,1.14)	0.38	1.08 (1.05,1.12)	0.15
Pancreas	3,172	1.01 (0.89,1.15)	0.63	1.00 (0.93,1.07)	0.42	1.01 (0.96,1.07)	0.08	1.01 (0.98,1.05)	0.00
Kidney	2,069	1.15 (1.09,1.21)	0.27	1.14 (1.08,1.20)	0.30	1.31 (1.18,1.45)	0.84	1.30 (1.18,1.44)	0.84
Lung	12,894	0.89 (0.85,0.93)	0.28	0.91 (0.88,0.94)	0.12	0.84 (0.80,0.89)	0.66	0.87 (0.83,0.90)	0.41
Endometrial	6,138	1.20 (1.14,1.27)	0.86	1.20 (1.14,1.27)	0.81	1.36 (1.21,1.53)	0.93	1.40 (1.21,1.62)	0.94
Ovarian	4,092	1.01 (0.97,1.05)	0.00	1.02 (0.97,1.07)	0.18	1.00 (0.91,1.1)	0.77	1.02 (0.93,1.12)	0.72
Post-menopausal breast cancer	25,512	1.01 (0.98,1.03)	0.30	1.01 (0.99,1.04)	0.36	1.05 (1.01,1.09)	0.62	1.07 (1.02,1.12)	0.72

*Multivariable adjusted models: baseline age, ethnicity, alcohol, smoking, HRT.

† The sum of OBR and NOR cancer does not equal total cancers as non-melanoma skin cancers were excluded in the EPIC cohort analyses.

Abbreviations: OBR, obesity-related; NOR, non-obesity-related; CI, confidence interval; HR, hazard ratio; BMI, body mass index; MV, multivariable; SD, standard deviation.

Table S32: Hazard ratios of cancers per 100 obese-years and per 5-unit BMI, ABACus 2 Consortium.

Outcomes	Number of cancer events	Obese-years (per 100 kg-years/m^2)				BMI (per 5 units)			
		Age-adjusted HR (95% CI)	I^2	MV-adjusted HR (95% CI)	I^2	Age-adjusted HR (95% CI)	I^2	MV-adjusted HR (95% CI)	I^2
**Men**									
†Total Cancers	130,704	1.09 (0.95,1.26)	0.84	1.09 (0.94,1.26)	0.85	1.02 (0.99,1.05)	0.67	1.01 (0.98,1.05)	0.68
OBR-cancers	23,060	1.37 (1.25,1.50)	0.29	1.37 (1.25,1.50)	0.25	1.20 (1.19,1.21)	0.00	1.20 (1.19,1.21)	0.00
NOR-cancers	72,342	0.90 (0.80,1.01)	0.47	0.89 (0.79,1.00)	0.47	0.95 (0.89,1.02)	0.86	0.95 (0.89,1.01)	0.71
NOR-cancers excluding lung and prostate	9,663	1.04 (0.93,1.17)	0.00	1.04 (0.91,1.19)	0.00	1.05 (1.01,1.1)	0.00	1.04 (0.98,1.11)	0.13
**Specific cancer sites**									
Colorectal	11,063	1.49 (0.72,3.10)	0.81	1.49 (0.73,3.03)	0.80	1.18 (1.10,1.26)	0.47	1.18 (1.10,1.26)	0.42
Pancreas	3,351	1.19 (0.88,1.61)	0.00	1.19 (0.85,1.67)	0.06	1.09 (0.97,1.23)	0.00	1.09 (0.95,1.25)	0.09
Kidney	3,398	1.42 (1.25,1.61)	0.00	1.41 (1.24,1.60)	0.00	1.26 (1.15,1.39)	0.35	1.26 (1.16,1.38)	0.21
Bladder	2,787	1.14 (0.77,1.67)	0.00	1.14 (0.67,1.92)	0.00	1.11 (1.00,1.23)	0.00	1.10 (0.96,1.27)	0.00
Lung	14,545	0.90 (0.71,1.13)	0.19	0.89 (0.70,1.12)	0.21	0.85 (0.64,1.13)	0.89	0.83 (0.66,1.05)	0.90
Prostate	48,116	0.90 (0.63,1.28)	0.69	0.85 (0.73,0.98)	0.54	0.96 (0.83,1.11)	0.86	0.95 (0.85,1.07)	0.72
**Women**									
†Total Cancers	98,690	1.08 (1.04,1.13)	0.78	1.10 (1.06,1.14)	0.62	1.04 (1.01,1.07)	0.83	1.05 (1.02,1.09)	0.85
OBR-cancers	55,712	1.16 (1.13,1.20)	0.35	1.17 (1.14,1.20)	0.17	1.10 (1.07,1.13)	0.84	1.11 (1.08,1.14)	0.84
NOR-cancers	26,718	0.90 (0.79,1.02)	0.83	0.94 (0.84,1.05)	0.78	0.91 (0.82,1.01)	0.94	0.94 (0.84,1.05)	0.94
NOR-cancers excluding lung	13,736	1.01 (0.95,1.07)	0.04	1.04 (1.00,1.08)	0.00	0.99 (0.90,1.10)	0.83	1.01 (0.91,1.12)	0.77
**Specific cancer sites**									
Colorectal	10,026	1.14 (1.09,1.19)	0.00	1.12 (1.07,1.18)	0.00	1.08 (1.05,1.11)	0.13	1.08 (1.05,1.10)	0.07
Pancreas	3,172	1.04 (0.79,1.37)	0.63	1.00 (0.83,1.20)	0.42	1.01 (0.96,1.06)	0.02	1.01 (0.98,1.04)	0.00
Kidney	2,069	1.33 (1.25,1.42)	0.00	1.32 (1.26,1.37)	0.00	1.28 (1.17,1.40)	0.84	1.27 (1.16,1.39)	0.85
Lung	12,894	0.76 (0.67,0.87)	0.70	0.81 (0.72,0.91)	0.50	0.85 (0.79,0.92)	0.79	0.88 (0.83,0.93)	0.69
Endometrial	6,138	1.57 (1.35,1.82)	0.95	1.58 (1.38,1.82)	0.92	1.34 (1.19,1.5)	0.95	1.36 (1.19,1.56)	0.96
Ovarian	4,092	1.01 (0.90,1.13)	0.20	1.03 (0.92,1.14)	0.21	1.00 (0.91,1.10)	0.75	1.02 (0.93,1.12)	0.70
Post-menopausal breast cancer	25,512	1.03 (0.95,1.11)	0.36	1.05 (0.96,1.15)	0.44	1.05 (1.01,1.1)	0.67	1.07 (1.02,1.12)	0.74

*Multivariable adjusted models: baseline age, ethnicity, alcohol, smoking, HRT.

† The sum of OBR and NOR cancer does not equal total cancers as non-melanoma skin cancers were excluded in the EPIC cohort analyses.

Abbreviations: OBR, obesity-related; NOR, non-obesity-related; CI, confidence interval; HR, hazard ratio; BMI, body mass index; MV, multivariable; SD, standard deviation.

Table S33: Comparison of the obesity degree and duration per unit standard deviation, ABACus 2 Consortium.

Outcomes	Number of cancer events	Degree of Obese (per SD)				Duration of Obese (per SD)			
		Age-adjusted HR (95% CI)	I^2	MV-adjusted HR (95% CI)	I^2	Age-adjusted HR (95% CI)	I^2	MV-adjusted HR (95% CI)	I^2
**Men**									
†Total Cancers	130,704	1.02 (0.98,1.06)	0.75	1.02 (0.99,1.04)	0.58	1.02 (1.00,1.04)	0.45	1.02 (0.99,1.05)	0.53
OBR-cancers	23,060	1.01 (0.98,1.04)	0.42	1.02 (0.99,1.05)	0.38	1.04 (1.01,1.07)	0.18	1.04 (1.01,1.07)	0.01
NOR-cancers	72,342	1.00 (0.97,1.04)	0.62	1.01 (0.98,1.04)	0.45	1.01 (0.97,1.04)	0.59	1.01 (0.98,1.03)	0.30
NOR-cancers excluding lung and prostate	9,663	1.01 (0.93,1.10)	0.71	1.02 (0.93,1.11)	0.71	1.01 (0.98,1.04)	0.00	1.02 (0.99,1.05)	0.00
**Specific cancer sites**									
Colorectal	11,063	1.01 (0.97,1.06)	0.54	1.02 (0.97,1.06)	0.51	1.04 (0.94,1.15)	0.58	1.03 (0.97,1.10)	0.56
Pancreas	3,351	1.01 (0.96,1.07)	0.00	1.01 (0.96,1.07)	0.00	1.06 (0.99,1.13)	0.00	1.06 (0.99,1.13)	0.00
Kidney	3,398	1.00 (0.94,1.06)	0.24	1.00 (0.94,1.06)	0.20	0.99 (0.89,1.11)	0.31	1.00 (0.91,1.10)	0.19
Bladder	2,787	1.02 (0.90,1.15)	0.77	1.03 (0.91,1.17)	0.77	1.00 (0.97,1.04)	0.00	1.01 (0.98,1.06)	0.00
Lung	14,545	1.07 (0.98,1.17)	0.74	1.09 (1.01,1.17)	0.62	1.08 (1.00,1.17)	0.50	1.10 (1.04,1.15)	0.19
Prostate	48,116	0.99 (0.97,1.01)	0.00	0.99 (0.97,1.00)	0.00	0.98 (0.95,1.01)	0.24	0.98 (0.96,1.01)	0.14
**Women**									
†Total Cancers	98,690	1.02 (1.00,1.04)	0.63	1.03 (1.01,1.04)	0.39	1.02 (1.00,1.05)	0.64	1.03 (1.00,1.05)	0.68
OBR-cancers	55,712	1.02 (0.99,1.05)	0.81	1.02 (0.99,1.05)	0.76	1.03 (0.99,1.07)	0.79	1.03 (0.99,1.07)	0.84
NOR-cancers	26,718	1.01 (0.97,1.04)	0.27	1.01 (0.99,1.04)	0.16	1.00 (0.95,1.04)	0.52	1.01 (0.97,1.05)	0.47
NOR-cancers excluding lung	13,736	1.02 (0.99,1.05)	0.00	1.02 (0.98,1.06)	0.10	1.01 (0.98,1.04)	0.00	1.02 (0.98,1.06)	0.19
**Specific cancer sites**									
Colorectal	10,026	1.02 (0.99,1.06)	0.18	1.02 (0.98,1.06)	0.30	1.03 (0.99,1.07)	0.05	1.03 (0.99,1.07)	0.16
Pancreas	3,172	0.98 (0.88,1.10)	0.52	0.98 (0.87,1.11)	0.54	1.00 (0.91,1.10)	0.46	1.00 (0.91,1.11)	0.51
Kidney	2,069	1.01 (0.93,1.10)	0.46	1.01 (0.94,1.10)	0.42	1.05 (0.96,1.15)	0.33	1.05 (0.96,1.14)	0.26
Lung	12,894	0.98 (0.93,1.03)	0.28	0.99 (0.96,1.03)	0.00	0.98 (0.94,1.03)	0.21	1.00 (0.97,1.04)	0.00
Endometrial	6,138	1.09 (1.01,1.17)	0.80	1.09 (1.02,1.17)	0.78	1.15 (1.03,1.28)	0.82	1.14 (1.02,1.28)	0.80
Ovarian	4,092	1.05 (0.99,1.10)	0.00	1.05 (1.00,1.10)	0.00	1.08 (1.04,1.13)	0.00	1.09 (1.04,1.13)	0.00
Post-menopausal breast cancer	25,512	0.98 (0.94,1.03)	0.67	0.98 (0.94,1.03)	0.76	0.99 (0.94,1.04)	0.70	1.00 (0.95,1.05)	0.76

*Multivariable adjusted models: baseline age, ethnicity, alcohol, smoking, HRT.
† The sum of OBR and NOR cancer does not equal total cancers as non-melanoma skin cancers were excluded in the EPIC cohort analyses.
Degree of overweight is the cumulative sum of the number of BMI units ≥30 kg/m^2
Duration of overweight is the cumulative sum of the duration overweight (BMI ≥30. kg/m^2).
Abbreviations: OBR, obesity-related; NOR, non-obesity-related; CI, confidence interval; HR, hazard ratio; BMI, body mass index; MV, multivariable.

Table S34: Comparison of the obesity degree and duration per 10 units and per 10 years respectively, ABACus 2 Consortium.

Outcomes	Number of cancer events	Degree of Obese (per 10 units)				Duration of Obese (per 10 years)			
		Age-adjusted HR (95% CI)	I^2	MV-adjusted HR (95% CI)	I^2	Age-adjusted HR (95% CI)	I^2	MV-adjusted HR (95% CI)	I^2
**Men**									
†Total Cancers	130,704	1.01 (0.99,1.03)	0.90	1.01 (0.99,1.03)	0.90	1.03 (0.98,1.09)	0.73	1.03 (0.99,1.08)	0.68
OBR-cancers	23,060	1.02 (0.97,1.09)	0.95	1.02 (0.97,1.09)	0.95	1.12 (0.94,1.34)	0.91	1.12 (0.95,1.32)	0.89
NOR-cancers	72,342	1.00 (0.99,1.01)	0.00	1.00 (0.99,1.01)	0.00	1.01 (0.96,1.06)	0.43	1.01 (0.93,1.08)	0.52
NOR-cancers excluding lung and prostate	9,663	1.01 (0.94,1.08)	0.86	1.01 (0.96,1.07)	0.79	1.04 (0.96,1.13)	0.00	1.05 (0.99,1.11)	0.00
**Specific cancer sites**									
Colorectal	11,063	1.02 (0.96,1.10)	0.87	1.02 (0.96,1.1)	0.87	1.13 (0.85,1.5)	0.83	1.12 (0.87,1.44)	0.78
Pancreas	3,351	1.01 (0.89,1.15)	0.80	1.01 (0.91,1.12)	0.71	1.10 (0.94,1.27)	0.15	1.09 (0.94,1.27)	0.09
Kidney	3,398	1.03 (0.95,1.12)	0.89	1.03 (0.95,1.12)	0.89	1.15 (0.81,1.63)	0.84	1.14 (0.81,1.61)	0.83
Bladder		1.01 (0.94,1.09)	0.83	1.01 (0.94,1.09)	0.82	1.02 (0.92,1.12)	0.00	1.02 (0.93,1.12)	0.00
Lung	14,545	1.01 (1.00,1.02)	0.00	1.02 (0.99,1.04)	0.18	1.06 (0.78,1.44)	0.76	1.07 (0.76,1.52)	0.85
Prostate	48,116	1.00 (0.99,1.01)	0.04	1.00 (0.99,1.01)	0.04	0.99 (0.96,1.02)	0.00	0.98 (0.96,1.00)	0.00
**Women**									
†Total Cancers	98,690	1.00 (1.00,1.01)	0.88	1.00 (1.00,1.01)	0.88	1.03 (0.99,1.06)	0.74	1.03 (0.99,1.07)	0.76
OBR-cancers	55,712	1.01 (0.99,1.02)	0.92	1.01 (0.99,1.02)	0.92	1.05 (0.99,1.11)	0.82	1.05 (0.98,1.12)	0.86
NOR-cancers	26,718	1.00 (0.99,1.02)	0.61	1.00 (0.98,1.02)	0.62	1.00 (0.92,1.08)	0.55	1.01 (0.94,1.09)	0.48
NOR-cancers excluding lung	13,736	1.00 (1.00,1.00)	0.00	1.00 (0.98,1.02)	0.52	1.02 (0.98,1.06)	0.00	1.03 (0.99,1.07)	0.00
**Specific cancer sites**									
Colorectal	10,026	1.00 (0.99,1.02)	0.47	1.00 (0.99,1.02)	0.46	1.03 (0.96,1.11)	0.48	1.03 (0.95,1.12)	0.52
Pancreas	3,172	1.00 (0.96,1.04)	0.63	1.00 (0.96,1.04)	0.63	1.01 (0.85,1.21)	0.56	1.03 (0.84,1.26)	0.61
Kidney	2,069	1.01 (0.99,1.04)	0.59	1.00 (0.99,1.02)	0.17	1.08 (0.92,1.27)	0.63	1.07 (0.92,1.25)	0.53
Lung	12,894	0.99 (0.98,1.01)	0.10	1.00 (1.00,1.00)	0.00	0.99 (0.92,1.06)	0.41	1.00 (0.95,1.05)	0.00
Endometrial	6,138	1.03 (0.99,1.06)	0.92	1.02 (0.99,1.06)	0.93	1.22 (0.93,1.59)	0.95	1.21 (0.93,1.58)	0.94
Ovarian	4,092	1.01 (1.00,1.02)	0.00	1.01 (1.00,1.02)	0.00	1.10 (0.98,1.23)	0.37	1.10 (0.97,1.24)	0.42
Post-menopausal breast cancer	25,512	1.00 (0.98,1.02)	0.77	1.00 (0.98,1.02)	0.78	1.01 (0.93,1.08)	0.68	1.02 (0.94,1.11)	0.77

*Multivariable adjusted models: baseline age, ethnicity, alcohol, smoking, HRT.
† The sum of OBR and NOR cancer does not equal total cancers as non-melanoma skin cancers were excluded in the EPIC cohort analyses.
Degree of overweight is the cumulative sum of the number of BMI units ≥ 30 kg/m^2
Duration of overweight is the cumulative sum of the duration overweight (BMI ≥ 30 kg/m^2).
Abbreviations: OBR, obesity-related; NOR, non-obesity-related;CI, confidence interval; HR, hazard ratio; BMI, body mass index; MV, multivariable.

Table S35: Comparison of Harrell's C-statistic of metrics, ABACus 2 Consortium.

Harrell's C-statistic(95% CI)									
**Characteristic**	**Obese-years**	**Baseline BMI**	**Difference in c-statistic between baseline BMI and obese-years**	**Obese - years with baseline BMI**	**Difference in c-statistic between obese-years with BMI combined compared with obese-years.**	**Difference in c-statistic between obese-years with BMI combined and BMI**	**Degree of obesity**	**Duration of obesity**	**Difference in c-statistic between duration and degree of obesity**
**Men**									
Total Cancers	0.599 (0.553, 0.643)	0.601 (0.549, 0.651)	-0.001 (-0.004, 0.002)	0.600 (0.536, 0.661)	0.000 (-0.002, 0.003)	0.002 (-0.001, 0.005)	0.600 (0.548, 0.649)	0.602 (0.551, 0.650)	-0.000 (-0.004, 0.004)
OR-cancers	0.607 (0.572, 0.641)	0.609 (0.573, 0.644)	0.003 (-0.001, 0.007)	0.607 (0.563, 0.649)	0.003 (-0.000, 0.006)	0.000 (-0.000, 0.001)	0.607 (0.572, 0.641)	0.610 (0.578, 0.642)	0.002 (0.000, 0.004)
NOR-cancers	0.606 (0.547, 0.663)	0.610 (0.554, 0.663)	0.000 (-0.003, 0.004)	0.611 (0.549, 0.669)	0.001 (-0.001, 0.003)	0.000 (-0.003, 0.004)	0.607 (0.546, 0.664)	0.641 (0.549, 0.723)	0.001 (-0.003, 0.004)
NOR-cancers excluding lung and prostate	0.621 (0.546, 0.691)	0.621 (0.545, 0.692)	0.000 (-0.002, 0.002)	0.625 (0.538, 0.704)	-0.000 (-0.001, 0.001)	-0.000 (-0.001, 0.001)	0.621 (0.545, 0.691)	0.621 (0.545, 0.691)	0.000 (-0.001, 0.001)
**Specific cancer sites**									
Colorectal	0.626 (0.578, 0.671)	0.626 (0.586, 0.665)	0.002 (-0.002, 0.006)	0.627 (0.581, 0.670)	0.002 (-0.000, 0.005)	0.000 (-0.002, 0.002)	0.628 (0.582, 0.672)	0.629 (0.580, 0.676)	0.000 (-0.002, 0.002)
Pancreas	0.607 (0.492, 0.712)	0.609 (0.487, 0.718)	0.000 (-0.001, 0.002)	0.617 (0.530, 0.697)	0.001 (-0.002, 0.005)	0.008 (-0.009, 0.025)	0.607 (0.490, 0.713)	0.606 (0.477, 0.721)	-0.000 (-0.005, 0.004)
Kidney	0.588 (0.553, 0.621)	0.592 (0.566, 0.617)	0.014 (0.001, 0.027)	0.596 (0.568, 0.624)	0.018 (0.010, 0.027)	0.000 (-0.002, 0.002)	0.575 (0.544, 0.606)	0.578 (0.549, 0.606)	0.002 (-0.005, 0.009)
Bladder	0.709 (0.603, 0.796)	0.709 (0.600, 0.797)	0.000 (-0.003, 0.003)	0.708 (0.613, 0.788)	-0.000 (-0.003, 0.002)	0.000 (-0.003, 0.003)	0.709 (0.602, 0.796)	0.709 (0.606, 0.794)	0.000 (-0.002, 0.003)
Lung	0.720 (0.694, 0.744)	0.725 (0.695, 0.754)	0.005 (0.000, 0.009)	0.723 (0.671, 0.770)	0.006 (0.002, 0.011)	0.001 (-0.001, 0.003)	0.720 (0.693, 0.746)	0.720 (0.693, 0.746)	-0.000 (-0.001, 0.001)
Prostate	0.616 (0.563, 0.666)	0.616 (0.559, 0.670)	-0.001 (-0.006, 0.005)	0.604 (0.544, 0.661)	-0.001 (-0.005, 0.004)	-0.000 (-0.004, 0.003)	0.615 (0.561, 0.667)	0.611 (0.556, 0.664)	-0.001 (-0.006, 0.004)
**Women**									
Total Cancers	0.574 (0.551, 0.596)	0.581 (0.560, 0.601)	0.000 (-0.000, 0.001)	0.568 (0.530, 0.606)	0.000 (-0.001, 0.002)	-0.000 (-0.000, 0.000)	0.577 (0.557, 0.598)	0.579 (0.556, 0.600)	0.000 (-0.000, 0.001)
OR-cancers	0.564 (0.534, 0.593)	0.569 (0.541, 0.596)	0.003 (0.001, 0.005)	0.574 (0.549, 0.599)	0.003 (0.001, 0.005)	0.000 (-0.004, 0.005)	0.560 (0.529, 0.591)	0.565 (0.537, 0.593)	0.001 (0.000, 0.002)
NOR-cancers	0.644 (0.590, 0.694)	0.645 (0.589, 0.697)	0.003 (-0.000, 0.006)	0.639 (0.581, 0.693)	0.002 (-0.002, 0.006)	0.001 (-0.000, 0.002)	0.643 (0.590, 0.693)	0.643 (0.591, 0.693)	0.000 (-0.000, 0.001)
NOR-cancers excluding lung	0.596 (0.546, 0.643)	0.597 (0.547, 0.645)	0.001 (-0.002, 0.004)	0.600 (0.553, 0.646)	0.001 (-0.002, 0.003)	0.000 (-0.000, 0.001)	0.596 (0.547, 0.643)	0.596 (0.547, 0.643)	-0.001 (-0.002, 0.001)
**Specific cancer sites**									
Colorectal	0.621 (0.555, 0.684)	0.626 (0.570, 0.679)	-0.000 (-0.002, 0.001)	0.623 (0.564, 0.679)	-0.000 (-0.000, 0.000)	0.000 (-0.001, 0.001)	0.621 (0.555, 0.683)	0.622 (0.555, 0.684)	0.001 (-0.001, 0.002)
Pancreas	0.636 (0.588, 0.681)	0.636 (0.589, 0.681)	0.001 (-0.001, 0.002)	0.637 (0.590, 0.682)	0.001 (-0.000, 0.001)	-0.000 (-0.001, 0.000)	0.637 (0.590, 0.681)	0.637 (0.589, 0.682)	0.000 (-0.001, 0.001)
Kidney	0.586 (0.555, 0.617)	0.612 (0.588, 0.635)	0.015 (0.003, 0.027)	0.615 (0.594, 0.635)	0.013 (0.002, 0.023)	-0.000 (-0.004, 0.003)	0.588 (0.559, 0.616)	0.590 (0.569, 0.612)	0.010 (0.001, 0.020)
Lung	0.743 (0.715, 0.769)	0.745 (0.717, 0.772)	0.003 (-0.001, 0.007)	0.739 (0.713, 0.763)	0.003 (-0.002, 0.007)	0.001 (-0.001, 0.002)	0.743 (0.716, 0.767)	0.744 (0.716, 0.770)	0.001 (-0.000, 0.002)
Endometrial	0.605 (0.572, 0.638)	0.625 (0.583, 0.665)	0.013 (0.000, 0.026)	0.623 (0.584, 0.660)	0.016 (0.004, 0.027)	0.000 (-0.000, 0.000)	0.602 (0.564, 0.639)	0.612 (0.578, 0.645)	-0.002 (-0.014, 0.010)
Ovarian	0.577 (0.531, 0.622)	0.575 (0.528, 0.621)	0.000 (-0.003, 0.004)	0.580 (0.532, 0.627)	0.000 (-0.003, 0.003	0.001 (0.000, 0.001)	0.575 (0.526, 0.623)	0.576 (0.527, 0.624)	0.002 (-0.001, 0.005)
Post-menopausal breast cancer	0.585 (0.513, 0.654)	0.589 (0.520, 0.654)	0.004 (0.001, 0.006)	0.589 (0.519, 0.657)	0.003 (0.001, 0.006)	-0.000 (-0.000, 0.000)	0.586 (0.513, 0.655)	0.586 (0.515, 0.653)	0.000 (-0.001, 0.001)

*All models were multivariable adjusted, including baseline age, ethnicity, alcohol, smoking, HRT.
Key: Green – significant difference in C-statistic.
Abbreviations: SE, standard error; OR, obesity-related; NOR, non-obesity-related; BMI, body mass index.
